# A Novel Integrated Microwave/UV/In Situ Fourier Transform Infrared Platform: Application to 1,5-Benzodiazepines Synthesis

**DOI:** 10.1021/acsomega.6c07314

**Published:** 2026-09-30

**Authors:** Alina Cherniienko, Kacper Kossakowski, Lucjusz Zaprutko, Roman Lesyk, Anna Pawełczyk

**Affiliations:** † Department and Chair of Organic Chemistry, 37807 Poznan University of Medical Sciences, 61-701 Poznan, Poland; ‡ Doctoral School of Poznan University of Medical Sciences, Poznan University of Medical Sciences, 61-701 Poznan, Poland; § Department of Pharmaceutical, Organic and Bioorganic Chemistry, Danylo Halytsky Lviv National Medical University, 79-010 Lviv, Ukraine; ∥ Department of Biotechnology and Cell Biology, University of Information Technology and Management in Rzeszow, 32-225 Rzeszow, Poland

## Abstract

The real-time monitoring and optimization of synthesis remain significantly limited by conventional “black-box” trial-and-error strategies. This study introduces an unprecedented instrumental triad: the novel integration of quantitative real-time, in situ attenuated total reflectance Fourier-transform infrared (ATR-FTIR) spectroscopy with simultaneous microwave (MW), and ultraviolet dual-activation. We deployed this multimodal platform to engineer an evidence-based, highly optimized synthesis of 1,5-benzodiazepines. Rather than relying on discrete offline sampling, continuous spectral tracking enabled rapid identification of ideal catalytic conditions, highlighting the green Lewis acid Sc­(OTf)_3_ (3 mol %) and molecular iodine (5 mol %) across diverse solvent environments. Crucially, the synergistic MW + UV dual-activation fundamentally outperformed single-mode thermal heating. In the model condensation of aromatic diamine and acetone, this physical synergy accelerated reaction completion to just 4.5 and 8.5 min, achieving isolated yields of 97 and 93% for Sc­(OTf)_3_ and I_2_, respectively. Furthermore, the incorporation of time-resolved ATR-FTIR coupled with standard addition protocols enabled precise quantitative tracking of functional group transformations and provided direct empirical evidence of catalyst–substrate coordination, as demonstrated by the dynamic spectral shift of the triflate ν_s_SO_3_ band. By mapping kinetic bottlenecks, solvent effects, and physical catalyst limitations, this novel multimodal approach offers a powerful, sustainable, and highly tunable paradigm for the rapid preparation of pharmacologically relevant nitrogen heterocycles. Moreover, this apparatus approach is characterized by high design flexibility, enabling its further modification and adaptation to diverse areas of scientific research.

## Graphical abstract

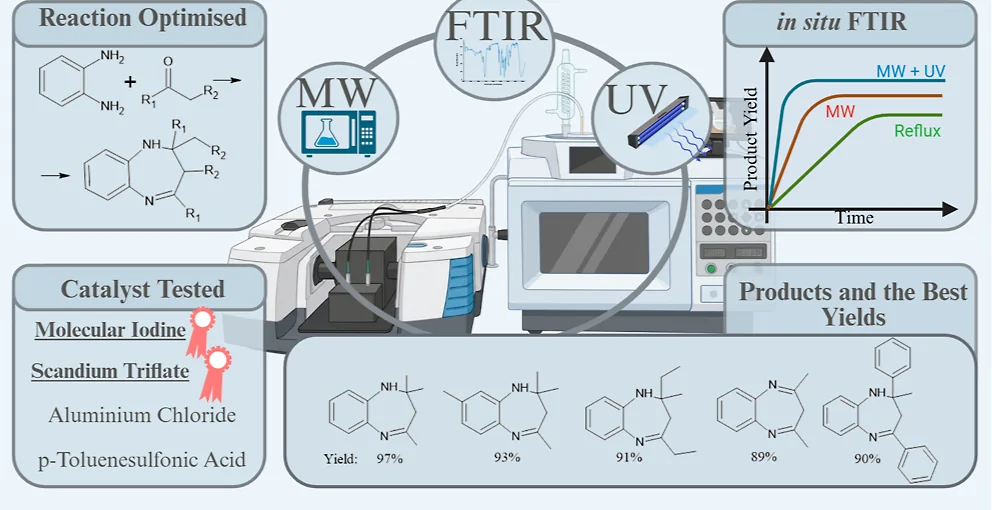

## 1 Introduction

The pursuit of efficient and rationally guided synthetic methodologies is a primary objective in modern organic chemistry.^1^ While the pharmaceutical and chemical sectors increasingly align with global trends (such as the UN Sustainable Development Goals^2^), translating these policies into laboratory practice requires moving beyond traditional trial-and-error experimentation. Currently, the synthesis of complex molecular scaffolds relies heavily on “black-box” methodologies characterized by energy-intensive, prolonged heating, destructive sampling, and offline characterization. These practices not only generate excessive waste but also fundamentally lack precise kinetic control (violating Green Chemistry Principle 6, which advocates the design of energy-efficient processes, and Principle 11, which mandates real-time, in-process analytical monitoring to prevent waste and pollution).^3^ As a consequence, critical short-lived intermediates and dynamic reaction pathways frequently remain inaccessible, compromising both the mechanistic understanding and process efficiency.

To overcome these limitations, this work introduces a pioneering multimodal synthetic-analytical platform that bridges the gap between high-performance catalysis and real-time process intensification. Central to this study is a custom-engineered instrumental setup (Figure 1), developed in-house, that, for the first time, creates an unprecedented triad: simultaneous microwave (MW) and UV dual activation continuously monitored by in situ time-resolved attenuated total reflectance Fourier-transform infrared (ATR-FTIR) spectroscopy. The integration of alternative energy sources, such as MW and ultraviolet (UV) irradiation, has emerged as a transformative strategy in classical synthesis. MW heating provides rapid, volumetric dielectric heating, significantly reducing reaction times and energy consumption compared to traditional techniques—directly addressing the need for energy efficiency.^4–6^ Concurrently, UV-mediated photoactivation provides a nonthermal pathway to excite molecular orbitals, enabling chemical transformations to proceed via lower-energy transitions that are often inaccessible through conventional heating.^7^ While MW and UV have been utilized individually, their simultaneous, synergistic dual-activation remains largely underexplored.^8^ Such dual-mode activation holds the potential to drastically accelerate chemical processes while maintaining high atom economy and minimizing side-product formation.^9–11^ Equally important to energy optimization is the rigorous control of the reaction process itself. The deployment of in situ, time-resolved analytical tools, such as Fourier transform infrared (FTIR) spectroscopy, provides a noninvasive, continuous feedback loop. In modern process engineering, real-time spectroscopic monitoring enables precise kinetic mapping and the immediate identification of reaction end points. This paradigm eliminates the reliance on destructive, offline sampling and minimizes reagent waste, serving as a critical component in the rational design of synthetic protocols.^12^

**Figure 1:**
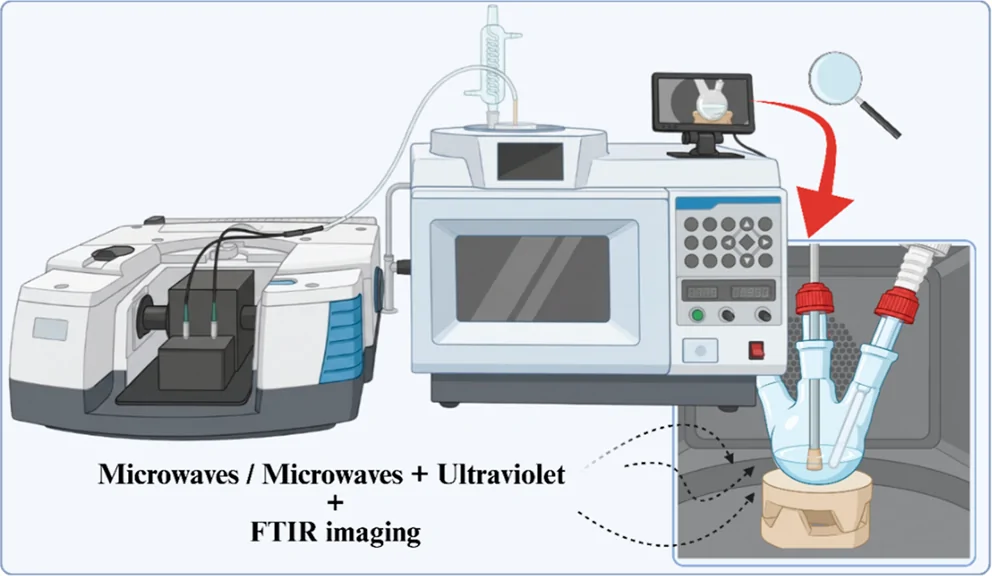
Synthetical-analytical system, consisting of an ATR-FTIR spectrometer connected by a fiber optic probe with a MW/UV (Created in BioRender. Pawełczyk, A. (2026) https://BioRender.com/xbc39b1).

To validate the efficiency of this multimodal synthetic-analytical platform, the 1,5-benzodiazepine (1,5-BD) scaffold serves as an ideal, pharmacologically vital target. As a privileged structure in medicinal chemistry, the 1,5-BD framework possesses a remarkably broad pharmacological spectrum (Figure 2).^13^ While the isomeric 1,4-benzodiazepines are primarily renowned for their central nervous system (CNS) activity, the 1,5-isomers exhibit a more diverse range of therapeutic potentials. This includes established CNS effects, as seen in the anticonvulsant clobazam, but extends significantly to potent antimicrobial, antiviral, and anti-inflammatory properties, exemplified by the anti-HIV drug nevirapine.^13^ Consequently, numerous synthetic methodologies have been developed to construct the 1,5-BD core, most classically via the cyclocondensation of *o*-phenylenediamines with ketones. Literature reports provide a wide array of catalytic systems that have been explored for this transformation, including homogeneous Lewis acids such as Er­(OTf)_3_,^14^ BiCl_3_,^15^ ZnCl_2_, YbCl_3_, and BF_3_·OEt_2_, as well as ionic liquids, heterogeneous solid acids (polyphosphoric acid on SiO_2_, sulfated zirconia, polymer-supported FeCl_3_, Amberlyst-15, and zeolites),^13^ and other catalytic systems, such as CH_3_COOH.^16^ However, despite this extensive literature, generating novel 1,5-BD libraries frequently encounters significant bottlenecks. Many of these established protocols still rely on harsh conditions, utilize corrosive reagents, require toxic, high-boiling solvents, or demand prolonged heating cycles to achieve acceptable conversions. Furthermore, a substantial portion of these methods lacks mechanistic transparency, heavily depending on trial-and-error optimization that fails to account for transient intermediates, real-time kinetics, or dynamic catalyst deactivation. This underscores an urgent need for an evidence-based methodology that can streamline the production of these nitrogen-containing heterocycles while strictly adhering to the principles of modern process engineering.

**Figure 2:**
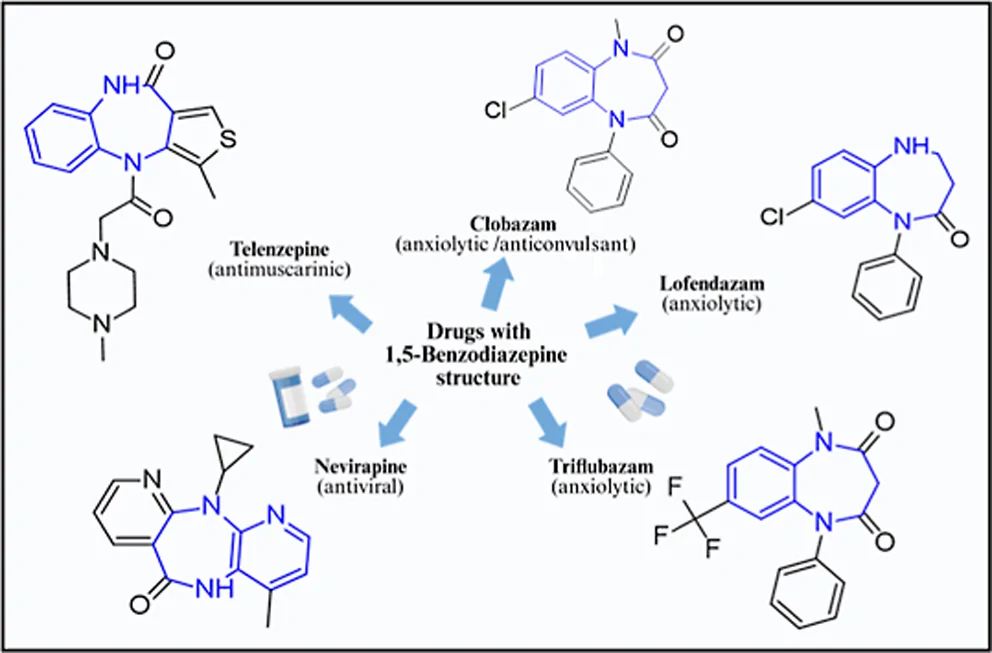
Examples of drugs containing 1,5-benzodiazepine scaffold (Created in BioRender. Pawełczyk, A. (2026) https://BioRender.com/3l8ctaa).

Utilizing our novel platform, we present an optimized, evidence-based protocol for 1,5-BD synthesis (Figure 3). To establish robust synthetic guidelines, we conducted a systematic evaluation of diverse catalytic systems and solvent environments, mapping their impact on the reaction kinetics and isolated yields. By leveraging the quantitative capabilities of time-resolved FTIR, supported by rigorous calibration and standard addition protocols, this study aims to provide deep mechanistic insights into the N-heterocyclization process. This synergistic approach offers a highly tunable paradigm for the rapid preparation of pharmacologically relevant heterocycles, providing a rational framework to balance the synthetic efficiency with sustainable process engineering.

**Figure 3:**
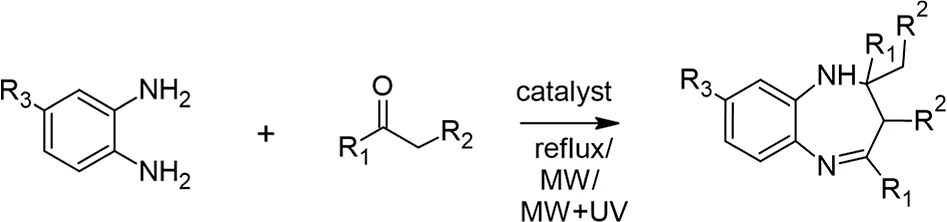
General synthesis of 1,5-benzodiazepine scaffolds under: (i) classical reflux conditions; (ii) MW-assisted conditions; (iii) MW + UV-simultaneous assisted conditions.

## 2 Experimental Section

### 2.1 Equipment

The research was conducted using a multifunctional chemical reactor, the UWave-1000 (SINEO Microwave Chemistry Technology, Shanghai, China), designed to perform chemical reactions under MW and/or ultraviolet (UV) conditions. This versatile system is equipped with a multimode MW generator (0–1000 W), an UV lamp (λ = 365 nm, 300 W), exhibiting an estimated effective optical power density of ∼3.6 W/cm^2^ at the reaction vessel interface (calculated based on standard radiometric efficiency and the liquid cross-sectional area), contact and noncontact thermometers, a magnetic stirrer, a cooling system, and a reaction visualization module. The UV-assisted reactions were performed in quartz glass containers (50 mL), ensuring optimal UV transmittance into the reaction mixture. All syntheses were carried out in the temperature-control mode at the solvent’s reflux temperature, where MW power was automatically adjusted via variable-frequency regulation according to the recorded temperature and reaction time. The heating rate was continuously monitored and adjusted through feedback control. The system operated in a nonpulsed, continuous MW heating mode. Importantly, all experiments were conducted in an open-vessel configuration at atmospheric pressure with a standard reflux condenser. This ensures that the observed kinetic enhancements stem directly from the applied MW heating or from synergistic MW + UV dual-activation and optimized catalyst–solvent interactions, rather than from pressure-induced effects.

Reaction monitoring and product analysis were performed using an ATR-FTIR Nicolet iS50 Tri-Detector Gold Flex Spectrometer (Thermo Scientific, Waltham, MA). The spectrometer was equipped with a diamond-tip ATR polycrystalline (AgCl/AgBr) fiber-optic probe ATR-G-D-6-35-150-Lab by Art Photonics and Fiber Probe Coupler FPC-6 M (all mirror design with beam height position 88 mm), enabling integration with the multifactor reactor to create a noninvasive, real-time analytical setup (Figure 1). The time-resolved spectra were comprising 64 scans, resolution 4.00 cm^–1^, gain 8.0, scanning speed 1.8988, and aperture 150.00. This instrument provides an interferometer speed of 65 scans/s and generates an effective optical path length of 1.5–3 μm, an angle of incidence of 45°, and a number of internal reflections of 2. The height of the signal kinetic profile relative to the baseline was used to monitor substrate and product concentrations. Spectral data were acquired and processed using Omnic software (version 9.12.928). For all experiments, the spectral range from 1900 to 650 cm^–1^ was selected for analysis. This specific window was chosen due to the inherent optical limitations of the diamond-tipped fiber-optic probe. Diamond exhibits strong intrinsic absorption between 2300 and 1900 cm^–1^, which severely attenuates the IR signal. Conveniently, the selected 1900–650 cm^–1^ range safely avoids this interference region while perfectly encompassing all critical functional group vibrations required to track the reaction progress, including the carbonyl (C=O) and imine (C=N) stretching frequencies.

### 2.2 Solvents and Chemicals

All solvents and reagents were obtained from Aldrich (Saint Louis, MO), Fluka (Buchs, Switzerland), Chempur (Piekary Śląskie, Poland), and POCh S.A. (Gliwice, Poland) and were of the highest available purity.

### 2.3 Synthetic Procedures

The study involved the condensation of ketones (Table 1) and aromatic diamines, *o*-phenylenediamine (*o*-PDA) and 4-methyl-*o*-phenylenediamine (4-Me-*o*-PDA) to yield 1,5-benzodiazepines (1,5-BD). For acetone (Ace) and ethyl methyl ketone (MEK), the compounds served both as solvents and substrates (10 mL each). For all other ketones, an additional solvent (methanol (MeOH) or acetonitrile (MeCN)) was used. Catalysts were chosen based on a literature search for common procedures and different types of catalysts: scandium­(III) triflate (Sc­(OTf)_3_)—a green Lewis acid catalyst^17^ known for its high activity, stability, and effectiveness,^18,19^ molecular iodine—mild Lewis, halogen-based, electrophilic, inexpensive, nontoxic, readily available catalyst,^20,21^ aluminum chloride (AlCl_3_)—strong Lewis acid catalyst^22^ and *p*-toluenesulfonic acid (*p*-TSA)—Brønsted acid frequently applied in heterocycle synthesis as an alternative to conventional inorganic acids, such as sulfuric acid, and Lewis acids.^23–25^ In addition, Montmorillonite K10 (MM K10),^26^ a solid acid clay catalyst with both Brønsted and Lewis acidic sites, was examined due to its widespread use in environmentally benign, heterogeneous catalytic transformations.

**Table 1:**
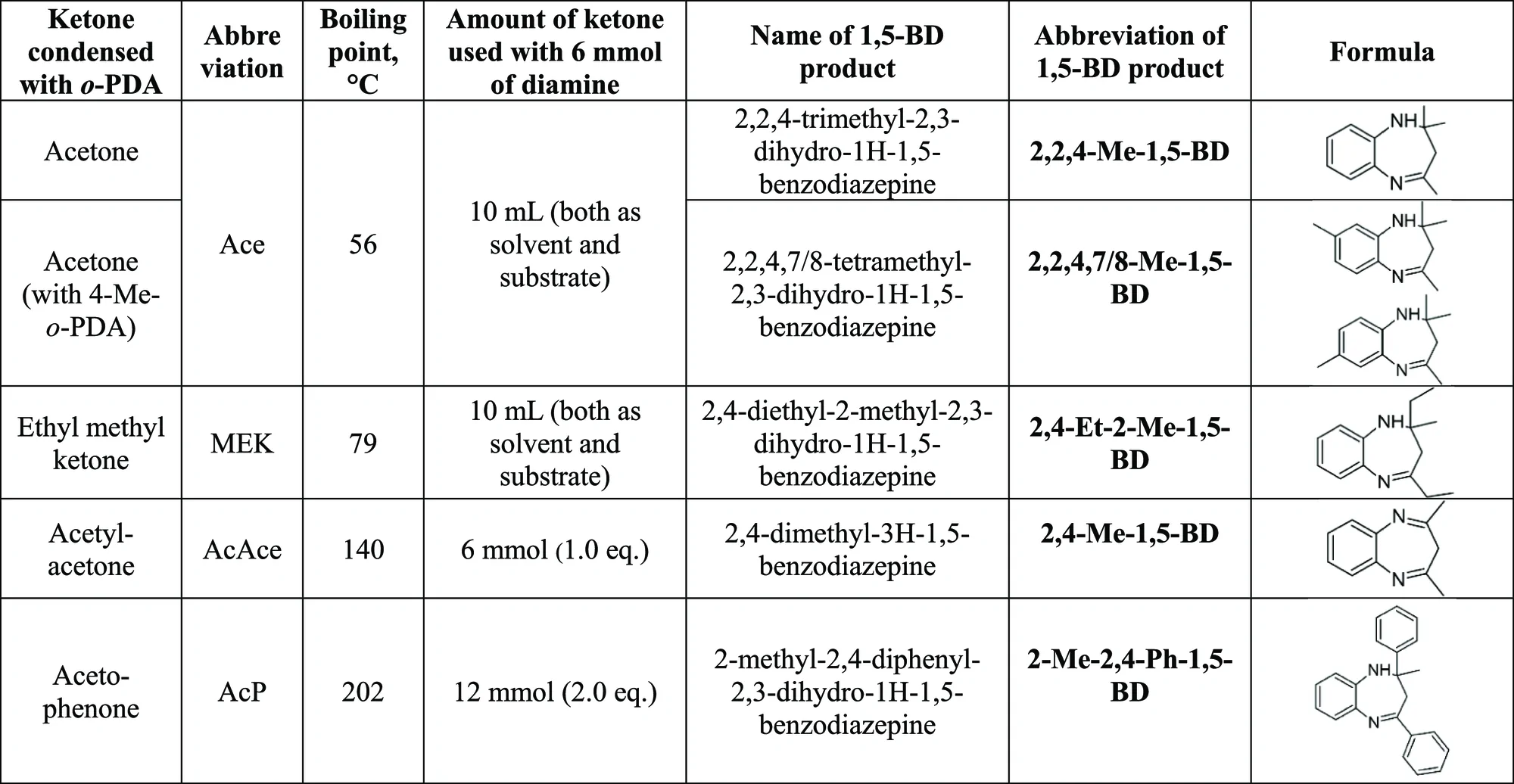
Ketones Used in the 1,5-Benzodiazepines (1,5-BD) Synthesis

#### 2.3.1 MW-Assisted and MW + UV-Assisted Method

Before the reactants were added, the background atmospheric spectrum was recorded. A reaction flask was charged with the selected ketone (and solvent (MeOH or MeCN) in case of AcAce and AcP) (amount specified in Table 1), together with a magnetic stirring bar, and IR recording was initiated. After 2 min, the catalyst dissolved in 1 mL of selected solvent was added (Sc­(OTf)_3_ 3 mol %, I_2_ 5 mol %, AlCl_3_ 10 mol %, or *p*-TSA 10 mol %, MM K10 20%), followed by the introduction of 6 mmol (0.649 g, 1.0 equiv) of aromatic diamine at the fifth minute, also dissolved in the 1 mL of the solvent. The reaction mixture was then heated by MW irradiation at 200 W (or coupled with UV irradiation at 365 nm) to the reflux temperature of the ketone (or solvent in case of AcAce and AcP), proceeding under continuous irradiation until full conversion (completion confirmed by *in situ* FTIR monitoring and double-checked after MW irradiation finish by thin-layer chromatography (TLC) analysis).

#### 2.3.2 Conventional Method

The procedure was identical to the MW-assisted method up to the addition of reagents. The reaction mixture was heated in a Heat-On Block System (Radleys, U.K.) at the ketone’s reflux temperature (or the solvent’s, in the case of AcAce and AcP). The reaction was continued until full conversion, monitored by *in situ* FTIR spectroscopy and verified by TLC after heating was complete.

Upon completion of the reaction, the solution was filtered to remove insoluble catalysts (e.g., MM K10) when applicable, and the solvent was removed under reduced pressure. The resulting crude residue was dissolved in dichloromethane and washed sequentially with distilled water and brine. For reactions catalyzed by I_2_, the organic layer was additionally washed with a 10% aqueous sodium thiosulfate solution to quench residual iodine. The organic phase was then separated, dried over anhydrous MgSO_4_, filtered, and concentrated. Finally, the products were purified by column chromatography on silica gel 60 (63–200 μm, Merck) using a chloroform/methanol mobile phase (30:1, v/v for most ketones; 99:1, v/v for the AcP derivative). The purified product was characterized by *ex situ* FTIR, NMR, and mass spectrometry (MS) methods to confirm its structure and purity. The ^1^H and ^13^C NMR spectra were recorded using a Varian VNMR-S 400 MHz spectrometer (Agilent Technologies, Santa Clara, CA) operating at 400 and 100 MHz, respectively, with tetramethylsilane as the internal standard and CDCl_3_ as the solvent. The MS spectra were obtained using a Bruker Compact QTOF spectrometer (Bruker Corporation, Billerica, MA). The obtained data were in full agreement with literature reports^18,27–29^ and are presented in the Supporting Information. Reaction progress was additionally monitored by TLC on DC-Alufolien Kieselgel 60 F_254_ plates (Merck, Darmstadt, Germany).

### 2.4 Analysis Setup Procedures

During the experimental procedure, the reaction progress was monitored in real time by IR spectroscopy, with time-resolved measurements (16–64 scans) conducted under the following conditions: MW 200 W, MW 200 W + UV, and conventional reflux. Before all experiments, a background was obtained. Additionally, the final conversion and completion of the reaction were confirmed by TLC. The intensity profiles of selected characteristic peaks were meticulously recorded and archived for analysis. The reaction’s end point was determined by observing stabilization and peak intensities. Each experiment was repeated at least three times for every solvent-condition combination, and the results were evaluated for reproducibility.

The quantitative study was performed in two steps:

#### 2.4.1 Creating the Calibration Curve

A calibration experiment was performed to establish a quantitative relationship between the *in situ* FTIR signal intensity and the concentration of the 1,5-BD product. The experimental setup consisted of a reaction vessel equipped with a magnetic stirring bar and an FTIR instrument (Nicolet iS50 Tri-Detector, Thermo Scientific) with a fiber optic probe. The vessel temperature was maintained by using a Heat-On block. The FTIR probe was immersed directly into the solvent to ensure a reproducible optical path length.

To generate the curve, successive portions of the target product (e.g., **2,2,4-Me-1,5-BD**) were added to the vessel in increments, each dissolved in the same volume of solvent. After each addition, the system was allowed to equilibrate to ensure complete dissolution and a homogeneous distribution before spectral acquisition. The specific absorption band (e.g., 756 cm^–1^ for **2,2,4-Me-1,5-BD**) was selected as the analytical marker.

The measured absorbance intensities at this wavenumber were plotted against the calculated molar concentrations, demonstrating a strictly linear relationship (Figure 4). The resulting calibration function was imported into Thermo Scientific TQ Analyst software (version 9.12.116, Thermo Fisher Scientific Inc.) to enable quantitative estimation of product concentration using the Simple Beer’s Law analysis tool. To further validate the accuracy of this external calibration model within the actual reaction matrix, a standard-addition protocol was performed at the end of the reaction, confirming the reliability of the quantitative tracking.

**Figure 4:**
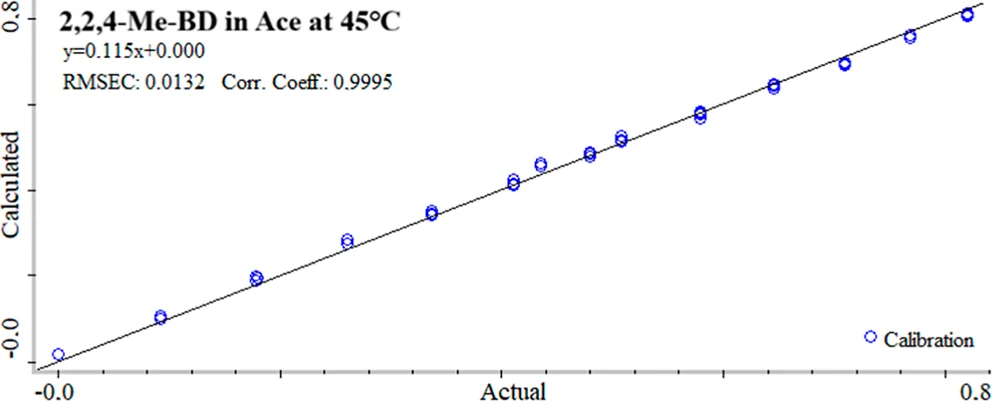
Example of calibration curve based on the height of peak 756 cm^–1^ of **2,2,4-Me-1,5-BD** in Ace at 45 °C with correlation coefficient 0.9995.

#### 2.4.2 Quantitative Analysis via Standard Addition Method

To ensure robust quantitative tracking within the dynamic reaction matrix, a standard addition method^30–32^ was employed using the purified reaction product as the standard. We followed the analytical framework proposed by Hutchinson et al.,^30^ but with a slight difference: instead of using the product’s peak signal, the peak’s height was considered a better option in this analysis. This approach relies on the principle of mass balance—the total amount of the product present after the standard addition ([P]_SA_·*V*_SA_) must equal the sum of the product formed at the end of the reaction ([P]_End_·*V*_End_) and the pure standard added *n*_SA_:

$${\left[P\right]}_{SA}\cdot V_{SA}={\left[P\right]}_{End}\cdot V_{End}+n_{SA}$$

Verification of the linear relationship between the analyte concentration and the corresponding FTIR signal was conducted according to a previously established calibration procedure. Given that the FTIR signal is proportional to concentration ([P]_*t*_ = *k*_P_·signal), substitution allows determination of the proportionality constant *k*_P_ in terms of known quantities

$$k_{P}=n_{SA}/\left\{\left(V_{SA}\cdot {signal}_{SA}\right)-\left(V_{End}\cdot {signal}_{End}\right)\right\}$$

The product concentration at any time point can then be calculated as

$${\left[P\right]}_{t}=n_{SA}/\left\{\left(V_{SA}\cdot {signal}_{SA}\right)-\left(V_{End}\cdot {signal}_{End}\right)\right\}\cdot {signal}_{t}$$

In these equations:

- signal_End_ and signal_SA_—the FTIR signals’ intensities at the end of the reaction and after the standard addition, respectively,
- *n*_SA_—the amount of product added as a standard,
- *V*_End_ and *V*_SA_—the reaction volumes before and after standard addition, respectively,
- [P]_End_ and [P]_SA_—the product concentrations at the end of the reaction and after standard addition, respectively.

FTIR spectra were recorded continuously until the reaction reached completion, as indicated by stabilization of the spectral profiles. Subsequently, the pure target product was introduced in two sequential portions (e.g., 1 mmol in 0.3 mL or 0.5 mmol in 0.2 mL of solvent). After each addition, the system was allowed 5–10 min to ensure complete dissolution and thorough mixing under continuous FTIR monitoring. The dynamic product concentration throughout the entire reaction profile was then retroactively calculated using the derived equations.

While the external calibration and standard addition methods described above were validated under homogeneous, interference-free conditions, two potential sources of quantification error merit explicit discussion. First, high concentrations of ionic species such as Sc^3+^ (as its triflate salt) or triiodide can, in principle, alter the bulk refractive index and dielectric response of the reaction medium, which in ATR-based measurements can manifest as a shift or slope in the recorded baseline independent of any genuine chemical change. To minimize this risk, all quantification bands used in this work were deliberately selected in spectral regions free of overlap with catalyst-associated absorptions, and standard baseline correction was applied throughout. Second, concentration drift—commonly arising from the volatilization of solvents or high-boiling substrates—was physically suppressed by the deployment of high-integrity reactor seals coupled with an active reflux condensation system. Continuous real-time spectral profiling of the solvent characteristic peaks confirmed negligible mass loss over the reaction timeline, validating the assumption of a constant-volume system.

## 3 Results and Discussion

The primary objective of this study was to design and validate a novel, multimodal synthetic-analytical platform that integrates real-time, in situ ATR-FTIR spectroscopy with simultaneous MW and ultraviolet (UV) dual activation. Rather than simply optimizing established protocols, this study aims to dismantle the empirical “black box” paradigm of heterocyclic synthesis by dynamically mapping kinetic responses to specific conditions and directly observing active catalyst coordination. Utilizing the 1,5-benzodiazepine (1,5-BD) scaffold as a robust pharmacological model, this unprecedented approach establishes an evidence-based, methodologically reproducible framework of reaction conditions for future research and practical applications.

### 3.1 Model Reaction and Catalyst Screening

In the model reaction between acetone (Ace) and *o*-PDA (Figure 5), clear differences in the catalytic performance were observed. Among the catalysts evaluated, Sc­(OTf)_3_ and I_2_ provided the most favorable outcomes, offering the shortest reaction times and the highest isolated yields under both MW-, MW + UV-assisted, and conventional reflux conditions. Despite their vastly different electronic profiles, both accelerate the condensation sequence by acting as Lewis acids to activate the ketone carbonyl toward nucleophilic attack. Exemplary heatmap and 3D map representing progress of the reactions with Sc­(OTf)_3_ at 200 W are presented in Figure 6. In the case of Ace and MEK, where the ketone boiling point is below 100 °C, and it can be easily recovered, we decided to use the ketone both as the substrate and as the solvent. This decision was made after comparing reaction yields and times when using acetone in MeCN versus Ace alone. The experiments demonstrated that employing Ace only resulted in significantly higher yields and shorter reaction times. Similar observations have been reported and substantiated in the scientific literature (82% yield in MeCN vs neat 98% in reflux in 3 h with Er­(OTF)_3_ as the catalyst^14^).

**Figure 5:**
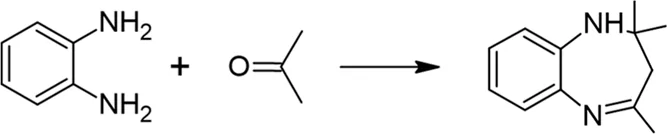
Reaction between acetone and *o*-PDA resulting in 2,2,4-trimethyl-2,3-dihydro-1*H*-1,5-benzodiazepine synthesis.

**Figure 6:**
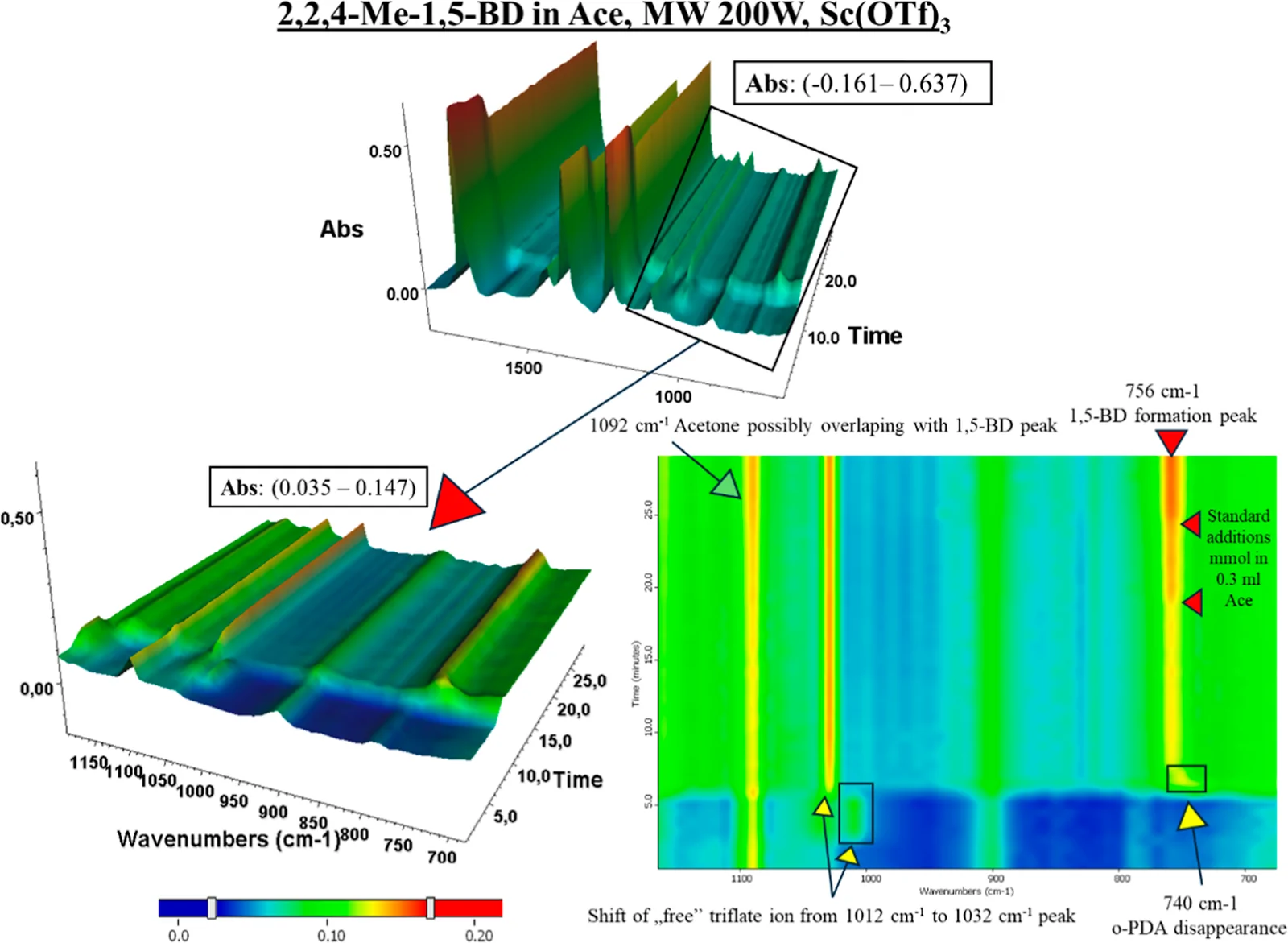
Exemplary corresponding FTIR heatmap and 3D map representing progress of the reaction with Sc­(OTf)_3_ at 200 W of MW.

AlCl_3_ and *p*-TSA enabled almost full substrate conversion; however, their performance was comparatively less efficient, reflected in longer reaction times, moderately reduced yields, and minor byproducts. While AlCl_3_ acts as a strong Lewis acid, its hard nature makes it prone to irreversible coordination with the basic *o*-PDA nucleophile, effectively trapping the catalyst and severely slowing turnover. Conversely, the strong Brønsted acid *p*-TSA activates the carbonyl via direct protonation but simultaneously protonates the diamine—temporarily neutralizing its nucleophilicity—while aggressively driving the competitive self-aldol condensation of acetone. MM K10 demonstrated catalytic performance that was notably less effective in this particular system, achieving incomplete conversion within a substantially prolonged reaction time frame. It is not likely that this is attributable to any genuine deficit in its acid site characteristics; pyridine-FTIR spectra for K10 routinely indicate both Bronsted (∼1545 cm^–1^) and Lewis (∼1454 cm^–1^) acidity of comparable magnitude to more active clay catalysts.^33–35^ The more probable limitation concerns substrate accessibility rather than acid site character. The majority of K10s acidic surface resides within its interlamellar galleries and internal porosity, meaning reactive substrates must diffuse into a confined solid matrix to reach active sites—a transport step absent for the fully mobile, molecularly dispersed Sc­(OTf)_3_ system. Therefore, it is likely mass transfer, and not inherently insufficient acid strength, which accounts for MM K10s relatively lower efficacy compared to that observed from heterogeneous catalysis broadly. As far as the monitoring aspect is concerned, the suspension of solid clay particles further compromised in situ FTIR tracking through two distinct physical mechanisms: bulk optical scattering and interfacial sensor blinding. First, the acid-activated MMT K10 particles form micron-scale tactoid aggregates. Because this particle size is on the same order of magnitude as mid-IR wavelengths (2.5–25 μm), the suspension induces intense Mie scattering.^36,37^ This scattering is severely compounded by the optical contrast between the solid clay (refractive index ∼1.5–1.6) and the liquid reaction medium (refractive index ∼1.33–1.4), causing significant baseline distortion and signal attenuation. Second, control experiments isolating MM K10 in nonevaporative MeCN (50 °C) revealed a severe physical sensor-blinding effect (Figure S34). Because the evanescent wave of the ATR crystal penetrates only 0.5–2.0 μm into the medium,^38^ the progressive gravitational settling and adhesion of the dense clay particles onto the diamond sensor physically displaced the liquid phase from the sampling volume. Time-resolved spectral monitoring demonstrated a continuous, massive accumulation of the intense Si–O–Si stretching vibration (1041 cm^–1^) associated with the clay, accompanied by the simultaneous attenuation of the liquid solvent bands (Figure S35). Ultimately, this irreversible interfacial fouling overwhelms the evanescent field with the solid’s own spectral signature, rendering accurate quantitative acquisition of the liquid-phase reaction kinetics impossible.

These findings are summarized in Table 2, which presents reaction times and isolated yields for each catalytic system. The superior catalytic performance of Sc­(OTf)_3_ can be attributed to its classification as a “hard” Lewis acid with pronounced oxophilicity. This characteristic allows the scandium center to coordinate effectively with the carbonyl oxygen of acetone, thereby increasing the electrophilicity of the carbonyl carbon more effectively than AlCl_3_. Similarly, the high efficacy of I_2_ likely stems from its role as a mild, halogen-based electrophilic catalyst that effectively activates the carbonyl group through halogen bonding, facilitating rapid initial condensation while avoiding the side reactions or rapid deactivation sometimes seen with stronger, moisture-sensitive Lewis acids. Graphic explanation of data processing (Figure 7) and corresponding FTIR time–intensity profiles (Figures 8 and 9) illustrate the impact of catalyst type and activation mode, revealing that MW irradiation consistently accelerates substrate consumption relative to conventional reflux, particularly in the presence of highly active Lewis acids. To verify that the physical immersion of the ATR-FTIR probe did not artificially perturb the reaction thermodynamics—such as by disrupting the electromagnetic MW field or scattering the UV irradiation—baseline model reactions were also performed in standard sealed MW vessels without the probe. The final isolated yields were identical to those achieved within the fully integrated multifactor reactor, confirming that the physical presence of the analytical hardware does not alter the intrinsic reaction efficiency or outcome.

**Table 2:** Comparison of 2,2,4-Trimethyl-2,3-dihydro-1*H*-1,5-benzodiazepine Synthesis from Acetone and *o*-PDA Synthesis in Different Conditions and Catalysts

catalyst	reaction in MW 200 W			reaction in reflux			
	reaction time (min)	isolated yield		reaction time (min)		isolated yield	
				mg	%^a^	mg	%^a^
Sc(OTf)_3_	6.5	1073	95		11	960	85
I_2_	11	1016	90		22	915	81
AlCl_3_	20	971	86		40	910	81
*p*-TSA	15	847	75		35	813	72

a To ensure operational reproducibility of primary model cyclocondensations on the subgram scale, reactions were performed in independent experimental triplicates; the standard deviations for these isolated yields were consistently determined to be ≤±4%, confirming high mechanical and kinetic reliability across the evaluated conditions.

**Figure 7:**
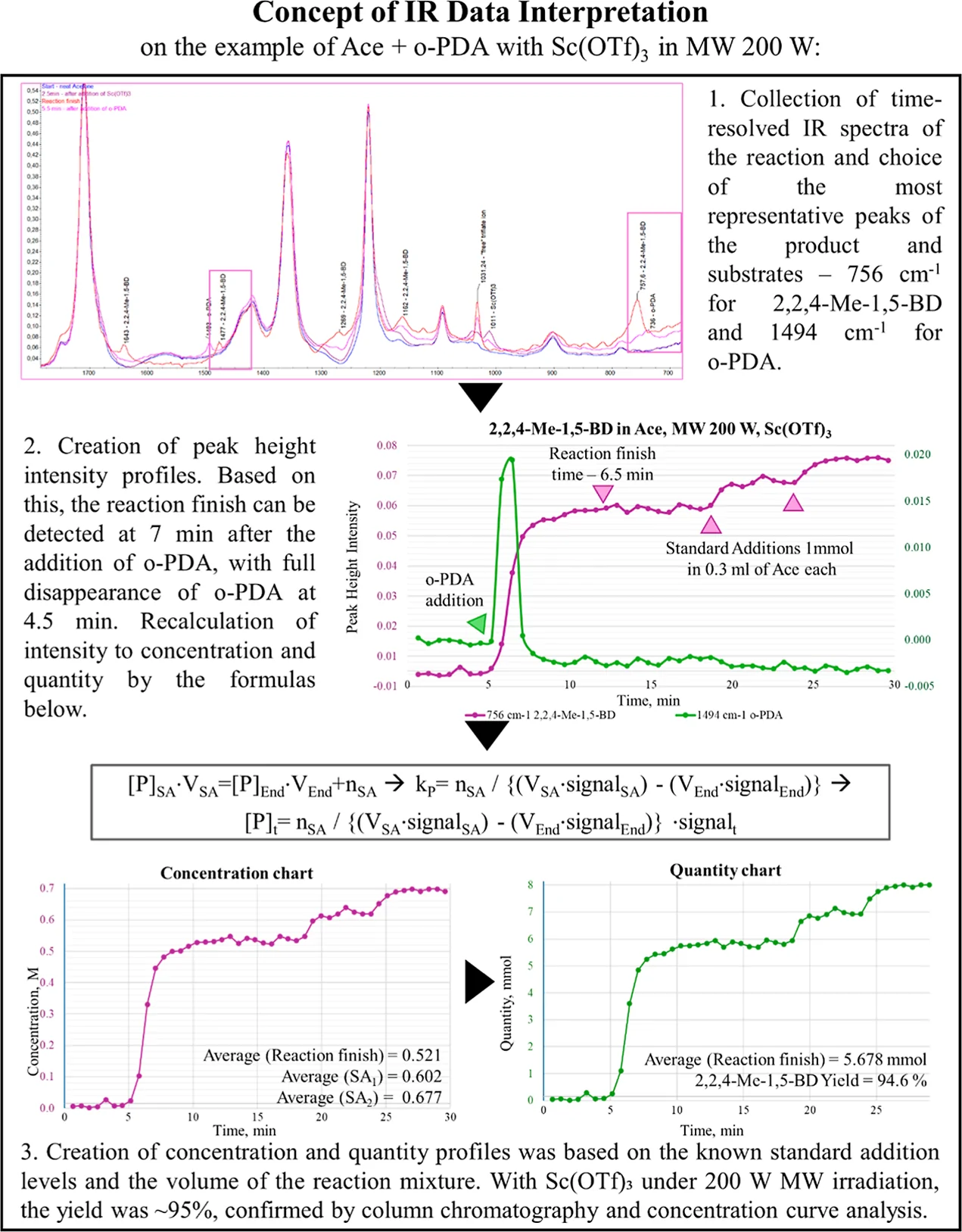
Concept of IR data interpretation: from raw spectra to product yield.

**Figure 8:**
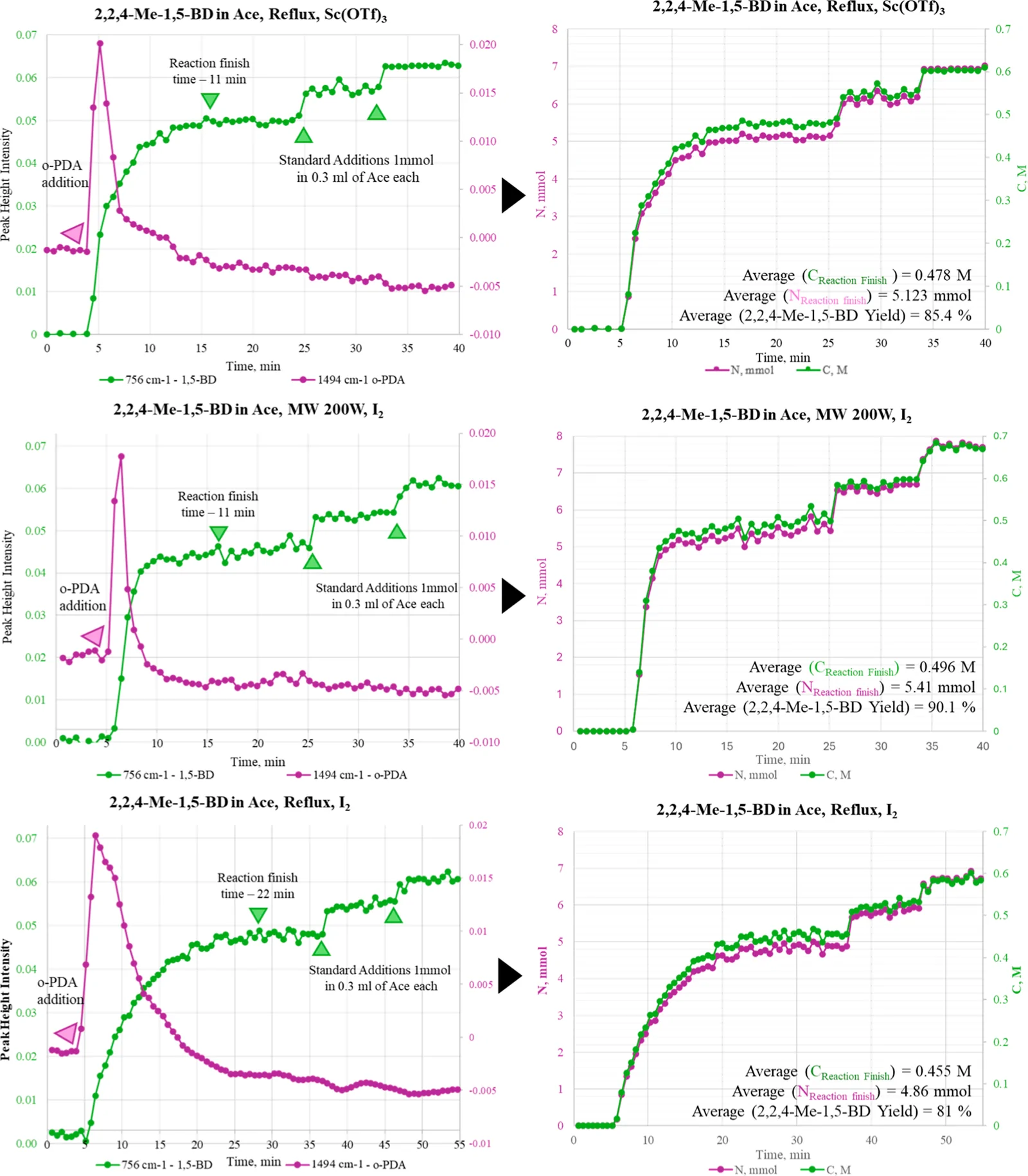
Corresponding FTIR time–intensity profiles and calculated concentration (*C*, M) and quantity (*N*, mmol) of 2,2,4-trimethyl-2,3-dihydro-1*H*-1,5-benzodiazepine synthesis from acetone and *o*-PDA under different conditions (MW and classical reflux) and catalysts (Sc­(OTf)_3_ and I_2_)—continuation of the concept from Figure 7.

**Figure 9:**
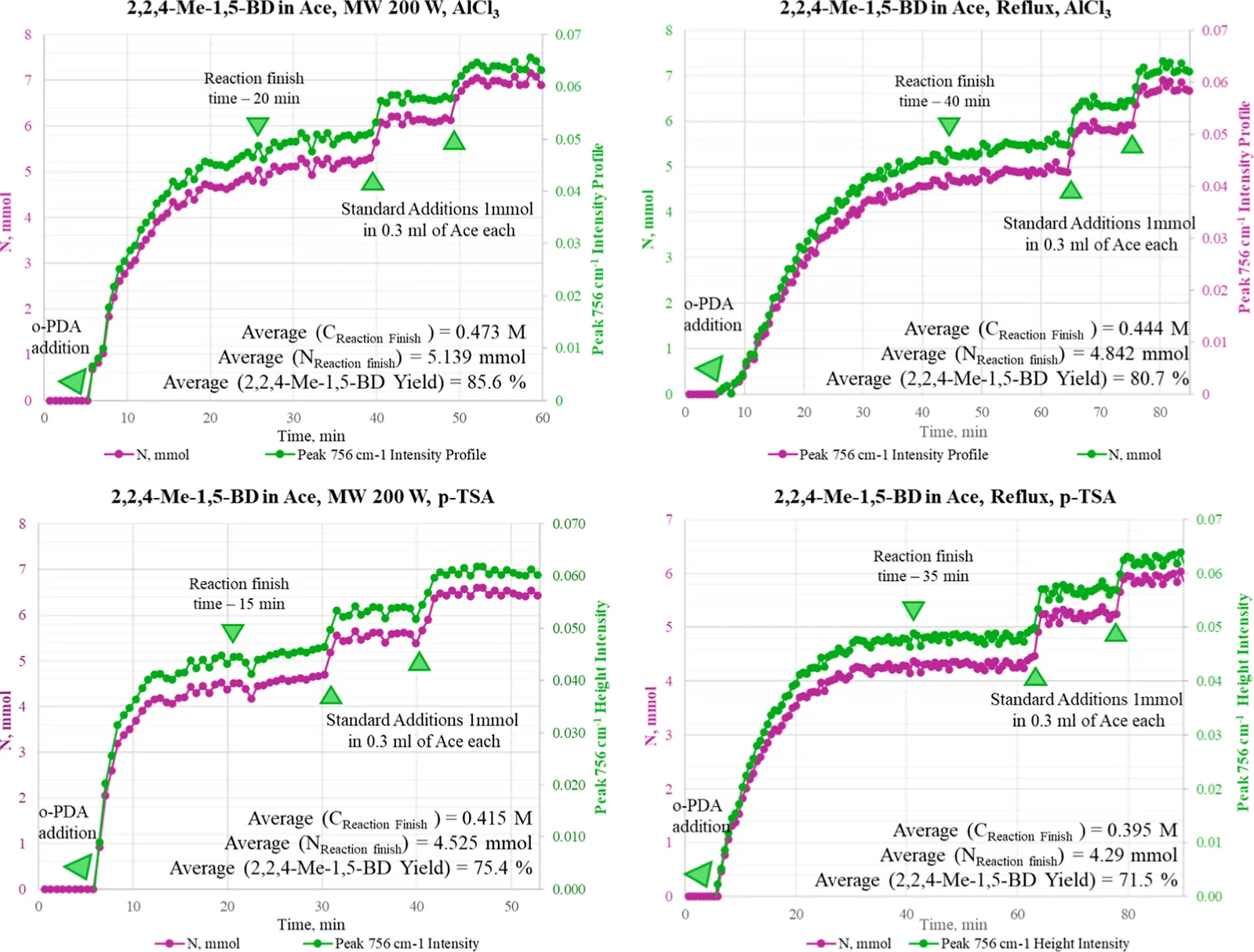
Corresponding FTIR time–intensity profiles and calculated quantity (*N*, mmol) of 2,2,4-trimethyl-2,3-dihydro-1*H*-1,5-benzodiazepine synthesis from acetone and *o*-PDA under different conditions and catalysts (AlCl_3_ and *p*-TSA).

The IR band at 756 cm^–1^ (baseline 834.5–725.5 cm^–1^) was selected for quantitative analysis of the **2,2,4-Me-1,5-BD** compound, corresponding to the aromatic C–H out-of-plane bending vibration characteristic of its benzene ring substitution pattern. Additional *in situ* IR features attributable to **2,2,4-Me-1,5-BD** were observed at 1640, 1477, 1268, 1124, and 817 cm^–1^. In contrast, the bands at 738 and 1494 cm^–1^ served as diagnostic markers of *o*-PDA consumption in the reaction mixture. During catalysis with Sc­(OTf)_3_, a moderately intense band at 1012 cm^–1^ was initially present. Upon introduction of *o*-PDA at the 5 min mark, this band shifted markedly to a sharp and intense signal at 1032 cm^–1^. This transformation is consistent with a change in the nondegenerate ν_s_ SO_3_ stretching mode associated with the “free” triflate ion (Figure 6), as previously described in the literature.^39–41^ Critically, this spectral transformation serves as a direct, empirical indicator of catalyst engagement. The shift confirms the transition of the triflate ion from a “free” state to a metal-complexed or actively coordinating state upon the addition of the substrate. By capturing this change dynamically, in situ FTIR data moves beyond simply tracking product formation; it provides real-time evidence of the catalyst actively altering its coordination sphere to interact with the amine substrate. This underscores the significant methodological value of this approach, enabling direct observation of the catalytic cycle in action.

### 3.2 Extension to β-Diketones and Solvent Effects

To rigorously stress-test the analytical versatility of the created platform, the substrate scope was extended from simple aliphatic ketones to 1,3-diketones, utilizing acetylacetone (AcAce) and *o*-PDA to synthesize 2,4-dimethyl-3*H*-1,5-benzodiazepine (**2,4-Me-1,5-BD**). This extension was deliberately designed to maintain a constant synthetic target—the 1,5-BD scaffold, while introducing drastic variations in both the physical matrix and mechanistic complexity. Physically, transitioning to a higher-boiling diketone necessitated the introduction of external solvents, which gives new analytical variables such as potential concentration drift, altered volatilization profiles, and diluted collision frequencies. Mechanistically, whereas the neat Ace transformation proceeds via a highly complex, multistep cascade involving aldol condensation, conjugate addition, and subsequent imine formation, the AcAce system undergoes a direct, highly efficient double-imine condensation. By successfully monitoring both of these fundamentally divergent N-heterocyclization pathways in real-time, we empirically demonstrate that the optimized catalytic system and the multimodal *in situ* analytical platform are highly robust and capable of profiling mechanistically distinct pathways across varying physical states (Figure 10). Unlike the model reaction with acetone, acetylacetone possesses a significantly higher boiling point (140 °C), making its use as both a neat substrate and solvent impractical for product isolation and recovery. Consequently, this transformation necessitated the introduction of an external solvent. Based on the superior performance observed in the model reaction, only the highly active Sc­(OTf)_3_ and the cost-effective I_2_ were selected for this and the next phases of the study. The choice of the solvent was guided by established literature. MeCN was selected due to its widespread reported efficacy in stabilizing polar intermediates in 1,5-BD syntheses.^14,19,42^ However, mindful of MeCN’s environmental and toxicological profile, MeOH was evaluated in parallel as a greener, protic alternative with a more favorable environmental impact.^43^ The comparative results of these reactions under both conventional reflux and MW irradiation are summarized in Table 3.

**Figure 10:**
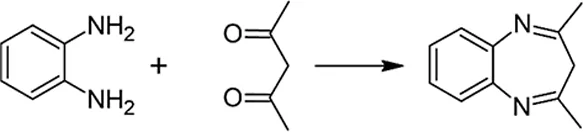
Reaction between acetylacetone and *o*-PDA results in the synthesis of 2,4-dimethyl-3*H*-1,5-benzodiazepine.

**Table 3:** Comparison of 2,4-Dimethyl-3*H*-1,5-benzodiazepine Synthesis from Acetylacetone and *o*-PDA under Different Conditions and with Different Catalysts and Solvents

solvent	reaction condition	Sc(OTf)_3_			I_2_		
		reaction time (min)	isolated yield		reaction time (min)	isolated yield	
			mg	%^a^		mg	%^a^
MeCN	MW 200 W	20	885	86	25	835	81
	Reflux	43	816	79	50	761	74
MeOH	MW 200 W	24	836	81	27	794	77
	Reflux	45	765	74	55	712	69

a To ensure operational reproducibility of primary model cyclocondensations on the subgram scale, reactions were performed in independent experimental triplicates; the standard deviations for these isolated yields were consistently determined to be ≤±6%, confirming high mechanical and kinetic reliability across the evaluated conditions.

The reaction kinetics were dynamically captured using *in situ* FTIR by tracking specific diagnostic bands. Substrate depletion was monitored by the disappearance of the AcAce enol band at 1622 cm^–1^, along with the disappearance of *o*-PDA markers at 1504 cm^–1^ (C=C skeletal stretching vibration) and 1281 cm^–1^ (aromatic C–N stretching vibration). Concurrently, product formation was confirmed by the emergence of a sharp C=N imine stretch at 1636 cm^–1^, along with additional characteristic peaks at 1267 cm^–1^ (C–N/C–C skeletal vibration) and 760 cm^–1^ (aromatic C–H out-of-plane bending vibration). These time–intensity profiles provided a high-resolution kinetic fingerprint of the reaction, directly substantiating the observed catalyst efficiencies (Figures 11 and 12).

**Figure 11:**
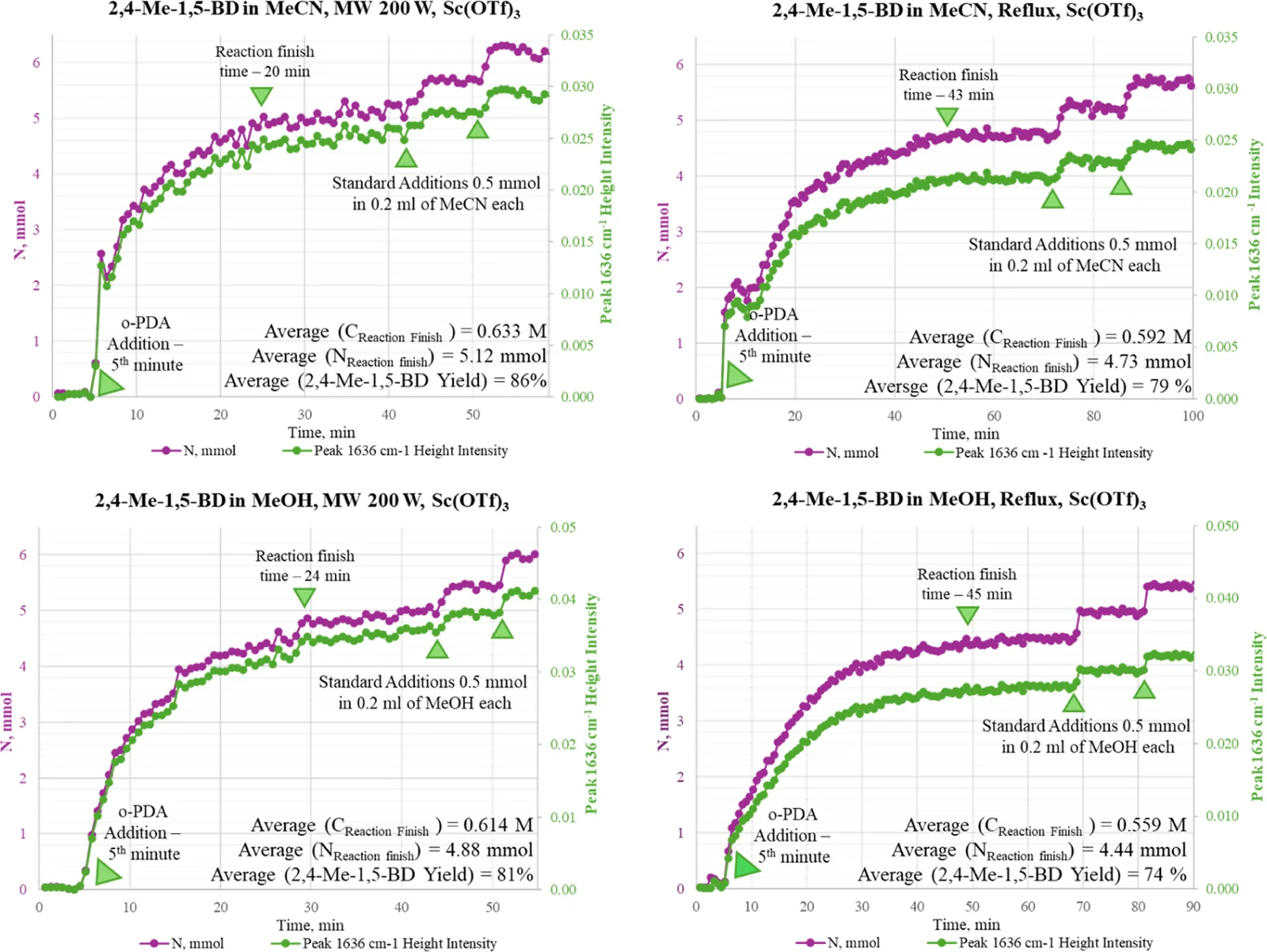
Corresponding FTIR time–intensity profiles and calculated quantity (*N*, mmol) of 2,4-dimethyl-3*H*-1,5-benzodiazepine synthesis from acetylacetone and *o*-PDA in different conditions and solvents with Sc­(OTf)_3_.

**Figure 12:**
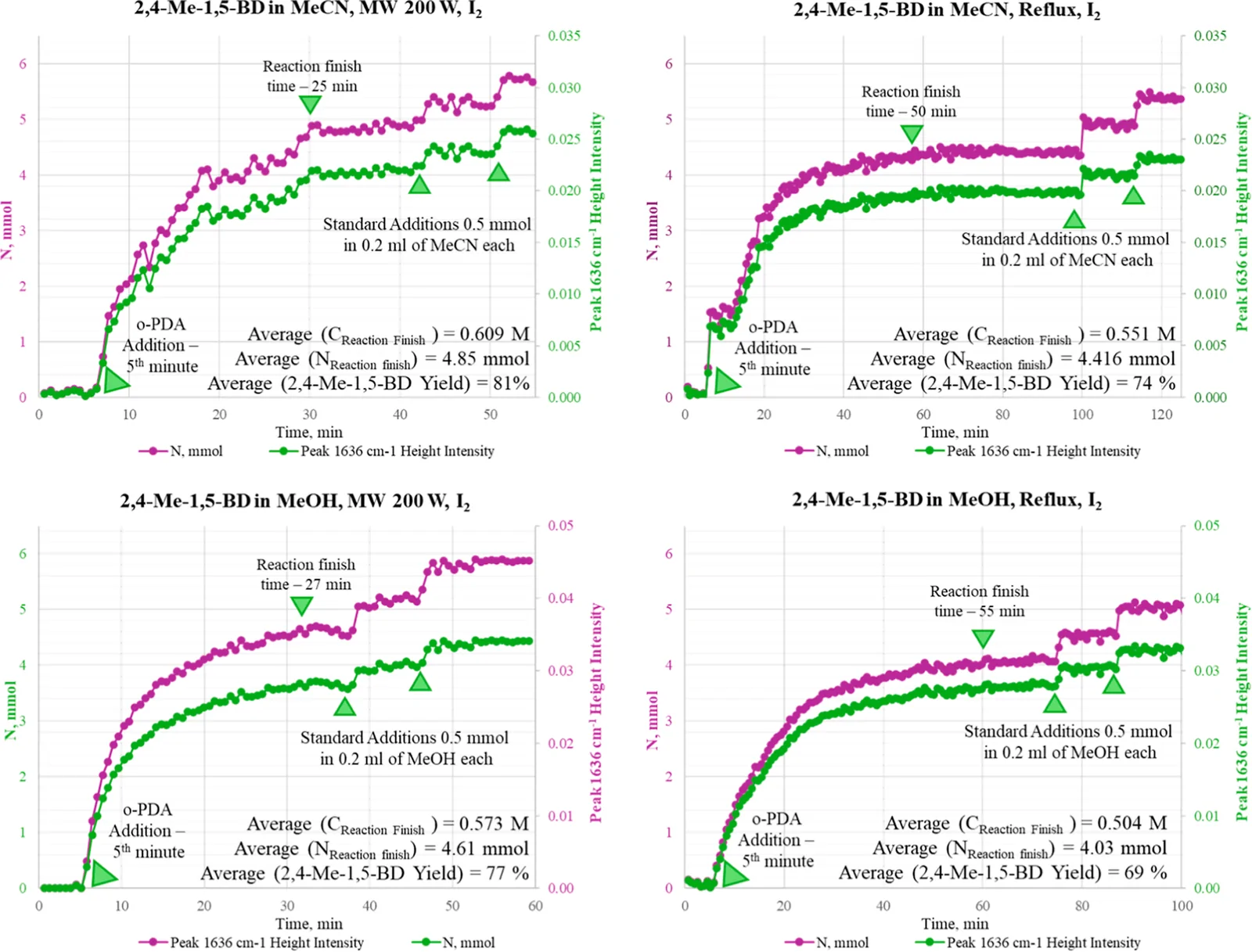
Corresponding FTIR time–intensity profiles and calculated quantity (*N*, mmol) of 2,4-dimethyl-3*H*-1,5-benzodiazepine synthesis from acetylacetone and *o*-PDA in different conditions and solvents with I_2_.

Consistent with the findings from the acetone model, also in the case of acetylacetone, Sc­(OTf)_3_ demonstrated a slight but distinct advantage over I_2_ across all tested conditions, achieving the highest yield of 86% in MeCN under MW irradiation in 20 min. While MeCN generally provided higher ultimate yields, MeOH proved to be a viable green alternative, yielding respectable conversions with comparable reaction times (e.g., 27 min 77% for I_2_ in MeOH at reflux vs 25 min 81% in MeCN). MW irradiation once again proved to be essential for process optimization, cutting reaction times by approximately half across all solvent and catalyst combinations.

As detailed in Table 3, both Sc­(OTf)_3_ and I_2_ consistently exhibited superior catalytic efficiency in MeCN compared to MeOH. This solvent-dependent kinetic behavior can be attributed to the divergent ways in which polar aprotic and polar protic solvents interact with the reaction components:

- Competitive inhibition in methanol: as a polar protic solvent, MeOH acts as a strong hydrogen-bond donor, which heavily solvates the nucleophilic lone pairs of *o*-PDA and substantially blunts its nucleophilicity. Crucially, this hydrogen-bonding environment disrupts the intramolecular hydrogen bond of acetylacetone, shifting its resting equilibrium away from the reactive enol tautomer and toward the less-reactive diketo form.
- Transition-state stabilization in MeCN: conversely, the polar aprotic nature of MeCN leaves the amine nucleophile fully active by avoiding strong hydrogen bonding. Furthermore, it preserves the intramolecular hydrogen bond of acetylacetone, maintaining a high concentration of the favored highly reactive enol tautomer. Rather than simply exhibiting “weaker coordination” to the catalysts, MeCN actively enhances electrophilicity—effectively stabilizing the charged hemiaminal intermediates and polar transition states during cyclization, MeCN systematically lowers the activation energy and accelerates the reaction velocity.

It is noteworthy that the overall reaction times for the AcAce system are noticeably longer than those observed for the neat Ace condensation. This kinetic deceleration is entirely attributed to the macroscopic reaction conditions rather than intrinsic mechanistic barriers. First, the introduction of a solvent inherently dilutes the reaction mixture; unlike the neat Ace reaction, where the ketone concentration was at a theoretical maximum, the solvated environment reduces the overall collision frequency. Second, the use of strictly equimolar amounts of substrates limits the thermodynamic drive that a massive excess of one reagent provides (pseudo-first-order kinetics). While the 1:1 intramolecular cyclization of the β-diketone is inherently more efficient than the 1:2 intermolecular cascade required by acetone, this mechanistic advantage is heavily outweighed by the kinetic penalties of dilution and strict stoichiometry.

### 3.3 Optimization of Catalyst Loading and Physical Limitations

Leveraging the continuous kinetic feedback of the *in situ* ATR-FTIR probe, the operational boundaries and physical limitations of Sc­(OTf)_3_ catalyst loading were systematically evaluated. A loading of 3 mol % emerged as the optimal threshold, providing the best balance between reaction velocity and system homogeneity. At lower catalyst concentrations (<3 mol %), the transformation proceeded at a markedly slower rate. Conversely, increasing the catalyst loading to 5 mol % and beyond did not yield any further improvements in reaction time or final conversion. Critically, at loadings of 5 mol % and higher, an unexpected physical limitation was encountered. The reaction mixture formed a distinct white precipitate, significantly impairing mass transfer and mixing efficiency. Furthermore, this precipitate strongly adhered to the ATR crystal of the IR probe, effectively blinding the sensor and rendering in situ FTIR monitoring impossible due to severe signal attenuation and scattering. We hypothesize that this behavior originates from the hydrolytic sensitivity of the Sc^3+^ center under combined MW irradiation and increasing byproduct concentrations, rather than simple solubility saturation of the triflate salt. Because triflate is a weakly coordinating anion, Sc^3+^ in solution remains predominantly solvated by water and the reaction solvent, causing its behavior to closely track that reported for the free Sc^3+^(aq) ion. Scandium possesses an unusually low p*K*_a_ (4.3) compared to larger lanthanides (e.g., La^3+^, p*K*_a_ 8.5) due to its small ionic radius and high charge density.^44^ As catalyst loading increases, the water generated stoichiometrically by the cyclocondensation raises the local water activity around the Sc^3+^ center past the threshold for the deprotonation of a coordinated water molecule. This olation step generates a bridging hydroxo ligand, producing dimeric [Sc_2_(μ-OH)_2_(H_2_O)_*n*_]^4+^ species.^45^ Continued olation and oxolation of this dimer yields progressively larger polynuclear Sc-oxo-hydroxo clusters that precipitate as amorphous scandium hydroxide (Sc­(OH)_3_) and oxyhydroxides. This hydrolytic susceptibility is consistent with established practice in scandium coordination chemistry, where aqueous Sc^3+^ complexation reactions are routinely conducted under mildly acidic buffered conditions (e.g., pH ≈ 5) specifically to suppress competing Sc­(OH)_3_/oxyhydroxide precipitation before chelation is complete.^46^ The strong adhesion of these species to the glass and the IR crystal is a serious limitation for the presented methodology. Thus, maintaining a 3 mol % loading is imperative not only for the catalytic efficiency but also to preserve the homogeneity required for reliable real-time spectroscopic monitoring.

In parallel with the optimization of the scandium system, the loading of molecular iodine (I_2_) was also evaluated. Consistent with observations from our previous studies, a 5 mol % loading of I_2_ emerged as the definitive optimal concentration. When the catalyst loading was increased to 10 mol %, the overall reaction efficiency notably deteriorated, characterized by moderately reduced isolated yields and a pronounced darkening of the reaction mixture. This decline in the performance at higher iodine concentrations stems from iodine’s dual reactivity profile. While it functions effectively as a mild Lewis acid—activating the carbonyl carbon via halogen bonding—it is also a well-known oxidant. The starting material, *o*-PDA, is notoriously sensitive to oxidative environments. At an elevated loading of 10 mol %, the increased I_2_ concentration begins to promote competing oxidative side reactions. This can lead to the formation of unreactive, halogen-bonded charge-transfer complexes that sequester the diamine. Consequently, excessive iodine actively consumes the starting material via these degradation pathways, outcompeting the desired condensation cycle and confirming 5 mol % as the kinetic and thermodynamic sweet spot.

### 3.4 Substrate Scope and Sustainability Trade-offs

To rigorously validate the novel platform, the methodology was intentionally deployed across a list of alternative 1,5-BD scaffolds (Table 4). Rather than pursuing an exhaustive, traditional, wide synthetic library, this proof-of-concept study utilized a deliberate selection of substrates engineered to stress-test the instrumental triad against distinct physical and electronic variables. By incorporating low and elevated boiling temperatures of ketones, varying aliphatic and aromatic, symmetrical and unsymmetrical ketone and diamine profiles, the platform’s capacity to dynamically monitor structurally diverse and mechanistically complex transformations was thoroughly evaluated.

**Table 4:** Comparison of Different 1,5-Benzodiazepine Scaffold Syntheses under Different Conditions and with Different Catalysts

catalyst	diamine	ketone	solvent^a^	reaction in MW 200 W			reaction in reflux		
				reaction time (min)	isolated yield		reaction time (min)	isolated yield	
					mg	%		mg	%
Sc(OTf)_3_/3 mol %	*o*-PDA	Ace	Ace	6.5	1073	95	11	960	85
		MEK	MEK	10	1181	91	30	1077	83
		AcAce	MeCN	20	885	86	43	816	79
			MeOH	24	836	81	45	765	74
		AcP	MeCN	25	1684	90	45	1553	83
	4-Me-*o*-PDA	Ace	Ace	6.5	1126	93	11	1018	84
I_2_/5 mol %	*o*-PDA	Ace	Ace	11	1016	90	22	915	81
		MEK	MEK	14	1128	87	40	1023	79
		AcAce	MeCN	25	835	81	50	761	74
			MeOH	27	794	77	55	712	69
		AcP	MeOH	45	1572	84	65	1347	72
	4-Me-*o*-PDA	Ace	Ace	11	1078	89	22	957	79

a For reactions involving acetone (Ace) and ethyl MEK, the ketones served both as substrates and solvents, with 10 mL of the respective ketone used together with 6 mmol *o*-PDA. For all other ketones, reactions were carried out using 12 mmol ketone and 6 mmol *o*-PDA in 8 mL of MeCN or methanol (MeOH) as the solvent.

Real-time spectral monitoring (with full kinetic profiles available in Supporting Information) confirmed that Sc­(OTf)_3_ consistently provided the shortest reaction times and highest yields across all the examined substrates, effectively overcoming the steric and electronic barriers. I_2_ exhibited only a slightly diminished performance in comparison. A parallel trend was observed in solvent selection: while MeCN consistently delivered superior kinetic profiles due to optimal transition-state stabilization, MeOH remained a highly viable, albeit slightly slower alternative. Ultimately, these modest differences in the efficiency must be weighed against the substantial disparities in the cost and environmental footprint. Sc­(OTf)_3_, while representing the pinnacle of catalytic activity and reliability in this study, is considerably more expensive and requires specialized handling and disposal because of its transition-metal content. By contrast, I_2_ is inexpensive, broadly accessible, and associated with a significantly lower ecological impact. When paired with MeOH, the I_2_ system perfectly aligns with the principles of green chemistry and resource-efficient synthesis.

The continuous analytical feedback from our platform underscores that the optimal synthetic protocol cannot be defined solely on the basis of traditional reaction metrics such as isolated yield or reaction time. Instead, the decision should be guided by the specific priorities of the researcher—whether maximal synthetic efficiency (Sc­(OTf)_3_ in MeCN), economic accessibility, and environmental sustainability (I_2_ in MeOH), or operational simplicity is paramount for a given application. Consequently, both catalytic systems and solvent choices remain highly viable, complementary options within our proposed evidence-based guidelines, enabling chemists to tailor the synthetic strategy to their specific constraints.

### 3.5 Testing of the MW + UV Synergistic Effect

To empirically validate the synergistic potential of multimodal physical activation, the in situ FTIR platform was deployed to continuously monitor the Sc­(OTf)_3_ and I_2_ catalytic systems under simultaneous MW and ultraviolet (UV) irradiation. Ultraviolet irradiation drives chemical transformations through a fundamentally different mechanism than dielectric or thermal heating; while the MW field provides rapid volumetric heating to stabilize highly polar transition states, UV light supplies sufficient energy to promote electrons into higher energy orbitals. By coupling these techniques, a powerful physical synergy emerges.^10,11,47,48^ Rather than relying on theoretical assumptions regarding these reaction pathways, our real-time kinetic tracking provided direct, quantitative evidence of this phenomenon, such as a reduced time of reaction and increased yield. It should be noted that sole UV irradiation without concurrent reflux/MW heating was excluded from comparative kinetic profiling. Under ambient thermal conditions, the solid starting materials failed to achieve complete dissolution. The resulting heterogeneous slurry physically scatters the IR beam at the crystal interface, blinding the sensor and rendering quantitative in situ ATR-FTIR acquisition impossible. Furthermore, preliminary screening confirmed that photochemical activation alone provides insufficient energy to effectively overcome the primary activation barrier of this cyclocondensation, resulting in unsatisfactory yields and reaction times. Therefore, reflux, MW, and MW + UV remain the only analytically viable modes for a reliable kinetic comparison.

To quantify this synergistic enhancement, the dual-activation approach was first applied to the model condensation of Ace and *o*-PDA (Table 5, Figure 13). Under simultaneous MW + UV conditions, both catalytic systems showed significant improvements in the reaction rate and overall efficiency. In the presence of Sc­(OTf)_3,_ complete conversion was achieved in an unprecedented 4.5 min, yielding 97% of the isolated 1,5-BD product (an improvement from 6.5 min and 95% under MW alone). The I_2_-catalyzed system demonstrated parallel enhancement, reaching completion in 8.5 min with 93% yield (improved from 11 min and 90% yield under MW alone). The pronounced kinetic acceleration observed under combined MW + UV irradiation strongly suggests a cooperative activation mechanism. The differing magnitude of enhancement between the two catalysts is consistent with distinct underlying photophysical origins rather than a single shared mechanism. For Sc­(OTf)_3_, coordination of the Lewis acidic Sc^3+^ center to the carbonyl oxygen lowers the energy of the π* orbital, red-shifting both the n → π* and π → π* absorption bands toward the near-UV region accessible at 365 nm, consistent with established Lewis-acid-activated carbonyl photochemistry.^49^ This excitation is proposed to further polarize the C=O bond, transiently enhancing electrophilic character at the carbonyl carbon and providing an additional, electronically driven contribution to lowering the activation energy for nucleophilic attack by *o*-PDA, alongside the purely thermal contribution supplied by MW heating. For I_2_, the more probable photoactive species is not the ketone itself—aliphatic ketones such as acetone show negligible absorption near 365 nm, but rather a charge-transfer complex formed between I_2_ (acceptor) and the electron-rich diamine *o*-PDA (donor), consistent with the well-established behavior of iodine-amine electron donor–acceptor complexes.^50^

**Table 5:** Comparison of Different 1,5-Benzodiazepine Scaffold Syntheses under Different Conditions and with Different Catalysts

ketone condensed with *o*-phenylenediamine	solvent	catalyst	reaction in MW 200 W			reaction in MW 200 W + UV 365 nm		
			reaction time (min)	isolated yield		reaction time (min)	isolated yield	
				mg	%		mg	%
acetone	acetone	Sc(OTf)_3_	6.5	1073	95	4.5	1094	97
	acetone	I_2_	11	1016	90	8.5	1050	93
acetylacetone	MeCN	Sc(OTf)_3_	20	885	86	18	914	89
	MeOH	I_2_	22	794	77	19	813	79

**Figure 13:**
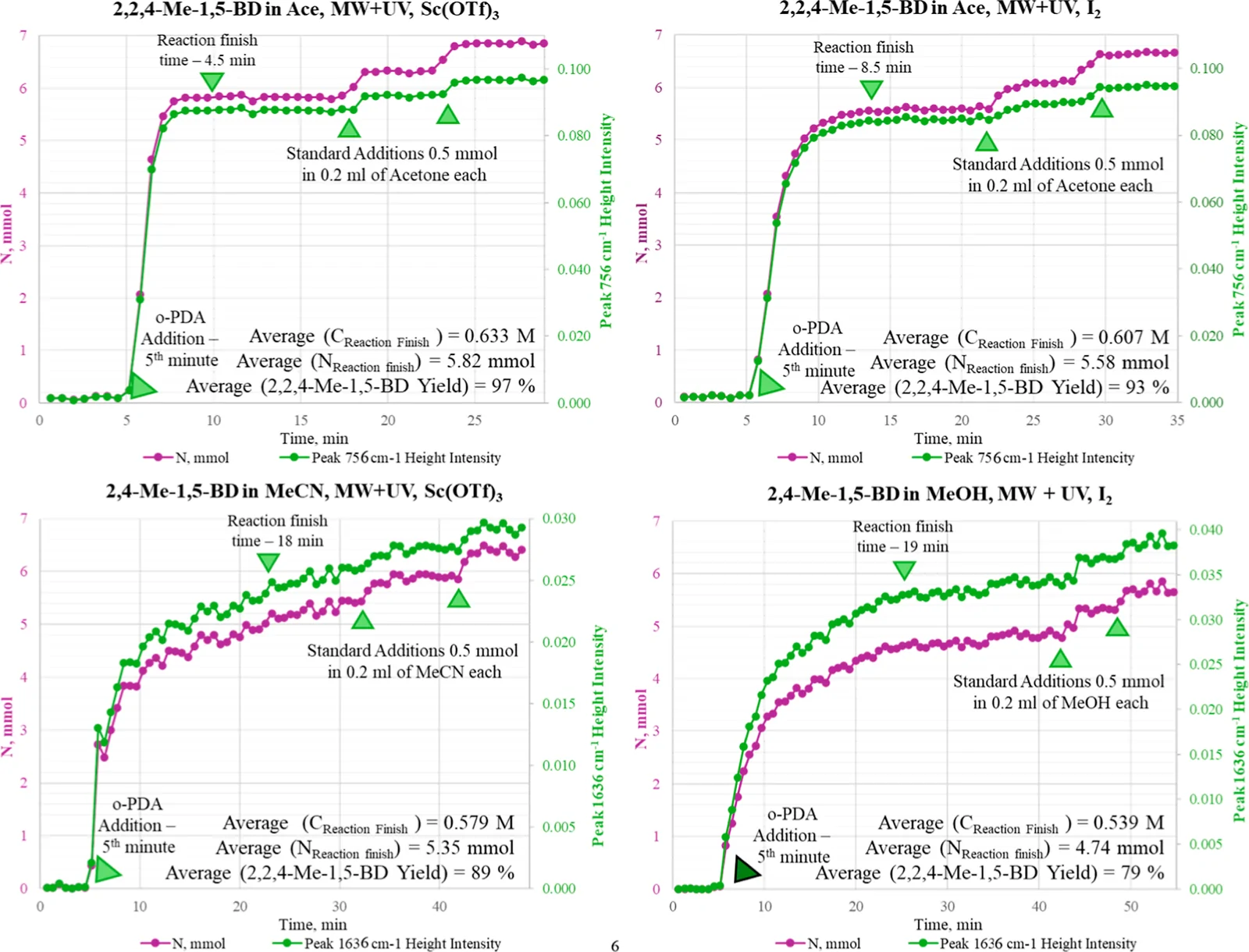
Corresponding FTIR time–intensity profiles and calculated quantity (*N*, mmol) of 2,2,4-trimethyl-2,3-dihydro-1*H*-1,5-benzodiazepine and 2,4-dimethyl-3*H*-1,5-benzodiazepine synthesis in the MW + UV synergistic mode and with Sc­(OTf)_3_ and I_2_.

To determine if this multimodal activation could similarly work with β-diketones, the acetylacetone and *o*-PDA reaction was subsequently evaluated under identical MW + UV conditions (Table 5, Figure 13). As anticipated, the synergistic MW + UV activation yielded measurable improvements for the acetylacetone system, albeit to a lesser extent than observed in the model acetone condensation. For the Sc­(OTf)_3_-catalyzed reaction in MeCN, the reaction time decreased from 20 to 18 min, with a modest yield enhancement from 86 to 89%. Similarly, the I_2_-catalyzed system in methanol reduced reaction time from 22 to 19 min, resulting in a slight increase in yield from 77 to 79%. While the dual-activation strategy successfully accelerated the transformation, this attenuated synergistic effect highlights the specific thermodynamic and physical barriers of the β-diketone system. Unlike the neat acetone model—which relies on a massive thermodynamic excess of solvent-as-reactant to force a complex intermolecular cascade—the acetylacetone reaction is an inherently efficient 1:1 cyclization that instead faces distinct ground-state hurdles. Furthermore, the introduction of an external solvent inherently dilutes the reactive species and reduces collision frequency in both systems, contributing to the overall slower absolute reaction times observed for the acetylacetone condensation regardless of the activation mode.

Nevertheless, the consistent reduction in reaction times across all tested substrates confirms that MW + UV dual activation remains a highly robust, universally applicable tool for optimizing complex syntheses, especially those leading to heterocyclic systems.

## 4 Conclusion

This study introduces a pioneering synthetic-analytical platform that fundamentally advances the paradigm of novel organic synthesis. By successfully integrating simultaneous MW and ultraviolet (UV) irradiation with real-time in situ ATR-FTIR spectroscopy, we have engineered an unprecedented, multimodal tool for the optimized synthesis of 1,5-benzodiazepines. This synergistic MW + UV dual-activation not only significantly accelerated reaction kinetics and improved isolated yields compared to conventional thermal methods, but it also maximized energy efficiency—a core tenet of modern synthesis.

Crucially, the novel application of continuous FTIR monitoring in this high-energy environment provided dynamic mechanistic insights, including capturing the active coordination state of the Sc­(OTf)_3_ catalyst. Furthermore, it identified strict operational boundaries, preventing reagent waste and side reactions by pinpointing ATR probe fouling at >3 mol % scandium loading and oxidative diamine degradation at >5 mol % iodine loading. Guided by these high-resolution, real-time analytics, two complementary evidence-based protocols were established, both emphasizing resource efficiency. For maximum synthetic velocity, Sc­(OTf)_3_ (3 mol %) in neat ketone or MeCN proved superior. Under single-mode MW irradiation, the model condensation of acetone yielded 95% in just 6.5 min; remarkably, simultaneous MW + UV irradiation further accelerated this process, delivering an exceptional 97% yield in only 4.5 min, thereby drastically reducing the overall energy consumption. Conversely, when prioritizing maximum sustainability and cost efficiency, molecular iodine (5 mol %) in neat ketone or methanol emerged as a highly effective, eco-friendly alternative. This greener catalytic route achieved an impressive 93% yield in just 8.5 min under dual activation.

Ultimately, this multimodal approach—coupling synergistic physical activation (MW + UV) with real-time spectroscopic feedback—transcends traditional, resource-heavy trial-and-error optimization. It provides a highly efficient and rational framework for the synthesis of pharmacologically relevant nitrogen-heterocycles. Furthermore, the modular architecture of this platform allows for seamless integration with additional analytical probes and operating parameters, establishing a highly adaptable foundation for broader applications. Moving forward, we plan to incorporate this powerful technique to optimize the synthesis of other pharmaceutically valuable and structurally more complex scaffolds.

## Supplementary Material



## Notes

Ex situ FTIR spectra for all compounds; NMR spectra for all compounds; mass spectra for all compounds; in situ FTIR spectra of compounds; kinetic profiles of the synthesis of compounds; photograph of the experimental setup; and time-resolved in situ ATR-FTIR spectral overlays demonstrating the progressive accumulation of Montmorillonite K10 (PDF)

## References

[1] Zimmerman J. B., Anastas P. T., Erythropel H. C., Leitner W. (2020). Designing for a Green
Chemistry Future. Science.

[2] Martinengo B., Diamanti E., Uliassi E., Bolognesi M. L. (2025). Medicinal
Chemistry: A Key Driver in Achieving the Global Sustainable Development
Goals. J. Med. Chem..

[3] Anastas, P. T. ; Warner, J. C. Green Chemistry: Theory and Practice; Oxford University Press, 2000. 10.1093/oso/9780198506980.001.0001.

[4] Kappe C. O. (2004). Controlled
Microwave Heating in Modern Organic Synthesis. Angew. Chem., Int. Ed..

[5] Ahmed S. A., Kamel M. S., Aboelez M. O., Ma X., Al-Karmalawy A. A., Mousa S. A. S., Shokr E. Kh., Abdel-Ghany H., Belal A., El Hamd M. A., Al Shehri Z. S., El Aleem Ali Ali El-Remaily M. A. (2022). Thieno­[2,3-b]­Thiophene Derivatives
as Potential EGFRWT and EGFRT790M Inhibitors with Antioxidant Activities:
Microwave-Assisted Synthesis and Quantitative In Vitro and In Silico
Studies. ACS Omega.

[6] El
Remaily M. A. E. A. A. A., Mohamed S. K. (2014). Eco-Friendly Synthesis
of Guanidinyltetrazole Compounds and 5-Substituted 1H-Tetrazoles in
Water under Microwave Irradiation. Tetrahedron.

[7] Hoffmann N. (2008). Photochemical
Reactions as Key Steps in Organic Synthesis. Chem. Rev..

[8] Horikoshi S., Serpone N. (2009). Photochemistry with
Microwaves. J. Photochem. Photobiol., C.

[9] Li M., Huang S., Zhang H., Wang L., Zhong S. (2020). DyPO4·1.5H2O
Microcrystals: Microwave/Ultrasound/ Ultraviolet Light- Assisted Synthesis,
Characterization and Formation Mechanism. Curr.
Microwave Chem..

[10] Genuino H. C., Hamal D. B., Fu Y.-J., Suib S. L. (2012). Synergetic Effects
of Ultraviolet and Microwave Radiation for Enhanced Activity of TiO_2_ Nanoparticles in Degrading Organic Dyes Using a Continuous-Flow
Reactor. J. Phys. Chem. C.

[11] Wang Y.-G., Jiang W.-J., Shen J., Wang W., Niu Y.-X., Zhao W., Wei X.-Y. (2021). Detoxification Modification of Coal-Tar
Pitch by Ultraviolet & Microwave Radiation-Enhanced Chemical Reaction
and Toxicity Evaluation by Chemical Index and Cytotoxicity Assay in
Vitro. J. Hazard. Mater..

[12] Cherniienko A., Lesyk R., Zaprutko L., Pawełczyk A. (2024). IR-EcoSpectra:
Exploring Sustainable Ex Situ and in Situ FTIR Applications for Green
Chemical and Pharmaceutical Analysis. J. Pharm.
Anal..

[13] Teli S., Teli P., Soni S., Sahiba N., Agarwal S. (2023). Synthetic
Aspects of 1,4- and 1,5-Benzodiazepines Using *o* -Phenylenediamine:
A Study of Past Quinquennial. RSC Adv..

[14] Nardi M., Cozza A., Maiuolo L., Oliverio M., Procopio A. (2011). 1,5-Benzoheteroazepines
through Eco-Friendly General Condensation Reactions. Tetrahedron Lett..

[15] Sun J., Li S., Meng M., Xue Q., Chen X., Qin Q., Liu X., Liu Q. (2023). Divergent Synthesis of 1,5-Benzodiazepines
and Benzimidazoles
via a BiCl3-Catalyzed One-Pot Condensation–Cyclization Process. Chem. Heterocycl. Compd..

[16] Pozarentzi M., Stephanidou-Stephanatou J., Tsoleridis C. A. (2002). An Efficient
Method for the Synthesis of 1,5-Benzodiazepine Derivatives under Microwave
Irradiation without Solvent. Tetrahedron Lett..

[17] Bayat M., Gheidari D. (2022). Green Lewis Acid Catalysis
in Organic Reactions. ChemistrySelect.

[18] De S. K., Gibbs R. A. (2005). Scandium­(III) Triflate
as an Efficient and Reusable
Catalyst for Synthesis of 1,5-Benzodiazepine Derivatives. Tetrahedron Lett..

[19] Hussain M. K., Ansari M. I., Kant R., Hajela K. (2014). Tandem C-2
Functionalization–Intramolecular
Azide–Alkyne 1,3-Dipolar Cycloaddition Reaction: A Convenient
Route to Highly Diversified 9 *H* -Benzo­[ *b* ]­Pyrrolo­[1,2- *g* ] [1,2,3]­Triazolo­[1,5- *d* ] [1,4]­Diazepines. Org. Lett..

[20] Peerzade N. A., Jadhav S. Y., Varpe B. D., Kulkarni A. A., Bhosale R. B. (2022). Green Synthesis,
Molecular Docking, *In Silico* ADME and Biological
Evaluation of Methoxy Substituted 1,5-Benzodiazepines as Potential
Antioxidant, Anti-Inflammatory, and Antidiabetic Agents. Polycyclic Aromat. Compd..

[21] Parveen P., Ptil V. A., Baseet M. A., Ahmed S. K. (2011). Mechanostic
Synthesis
of 1,5-Benzodiazepines Using Molecular Iodine. Int. J. Ind. Chem..

[22] More U. B., Kharat R. S., Mahulikar R. P. (2011). An Efficient Synthesis of 1,5-Benzodiazepines
Using AlCl3 Under Solvent Free Condition. Asian
J. Chem..

[23] Wang S., Hu L., Cheng S., Wang L. (2015). Synthesis of 1,5-Benzodiazepine Derivatives
Using p-Toluenesulfonic Acid as Catalyst. Asian
J. Chem..

[24] Pasha M. A., Jayashanka V. P. (2006). An Expeditious
Synthesis of 1,5-Benzodiazepine Derivatives
Catalysed by p-Toluenesulfonic Acid. J. Pharmacol.
Toxicol..

[25] Han C., Nie L., Han X., Zhang Y., Sun K., Shi L., Cui G., Meng W. (2021). One-Pot Three-Component Synthesis of Novel 1,5-Benzodiazepine
Derivatives and Their *Anti* -BVDV (Bovine Viral Diarrhea
Virus) Activity. Chin. J. Org. Chem..

[26] Shaikh I. N., Baseer M. A., Ahmed D. B., Farooq Adil S., Khan M., Alwarthan A. (2020). Microwave-Assisted Green Synthesis
of 1,5 Benzodiazepines Using Cu­(II)-Clay Nanocatalyst. J. King Saud Univ., Sci..

[27] 2,2,4-Trimethyl-2,3-Dihydro-1H-1,5-Benzodiazepine Compound with Spectra: NMR, FTIR, Raman, and MS (GC), 2025. https://spectrabase.com/compound/1xLNc4YortY. (accesed 28 10, 2025).

[28] 2,4-Dimethyl-1H-Benzo­[b]­[1,4]­Diazepine Compound with Spectra: 1 NMR, and 2 FTIR, 2025. https://spectrabase.com/spectrum/25ysrqsNw1s. (accesed 28 10, 2025).

[29] 2,4-Diphenyl-2-Methyl-2,3-Dihydro-1H-[1,5]-Benzodiazepine Compound with Spectra: 2 NMR, and 5 MS, 2025. https://spectrabase.com/compound/1JqjyWkQU0z. (accesed 28 10, 2025).

[30] Hutchinson G., Welsh C. D. M., Burés J. (2021). Use of Standard Addition to Quantify
In Situ FTIR Reaction Data. J. Org. Chem..

[31] Sato Y., Liu J., Kukor A. J., Culhane J. C., Tucker J. L., Kucera D. J., Cochran B. M., Hein J. E. (2021). Real-Time Monitoring of Solid–Liquid
Slurries: Optimized Synthesis of Tetrabenazine. J. Org. Chem..

[32] Sagmeister P., Melnizky L., Williams J. D., Kappe C. O. (2024). Simultaneous Reaction-
and Analytical Model Building Using Dynamic Flow Experiments to Accelerate
Process Development. Chem. Sci..

[33] Flessner U., Jones D. J., Rozière J., Zajac J., Storaro L., Lenarda M., Pavan M., Jiménez-López A., Rodríguez-Castellón E., Trombetta M., Busca G. (2001). A Study of the Surface Acidity of
Acid-Treated Montmorillonite Clay
Catalysts. J. Mol. Catal. A: Chem..

[34] Reddy C. R., Bhat Y. S., Nagendrappa G., Jai Prakash B. S. (2009). Brønsted
and Lewis Acidity of Modified Montmorillonite Clay Catalysts Determined
by FT-IR Spectroscopy. Catal. Today.

[35] Akçay M. (2005). The Surface
Acidity and Characterization of Fe-Montmorillonite Probed by in Situ
FT-IR Spectroscopy of Adsorbed Pyridine. Appl.
Catal., A.

[36] Othman A. M., Elsayed A. A., Sabry Y. M., Khalil D., Bourouina T. (2023). Detection
of Sub-20 Μm Microplastic Particles by Attenuated Total Reflection
Fourier Transform Infrared Spectroscopy and Comparison with Raman
Spectroscopy. ACS Omega.

[37] Bassan P., Kohler A., Martens H., Lee J., Byrne H. J., Dumas P., Gazi E., Brown M., Clarke N., Gardner P. (2010). Resonant Mie Scattering (RMieS) Correction
of Infrared
Spectra from Highly Scattering Biological Samples. Analyst.

[38] Harrick N. J. (1960). Surface
Chemistry From Spectral Analysis Of Totally Internally Reflected Radiation. J. Phys. Chem..

[39] Suthanthiraraj S. A., Kumar R., Paul B. J. (2009). FT-IR Spectroscopic Investigation
of Ionic Interactions in PPG 4000: AgCF3SO3 Polymer Electrolyte. Spectrochim. Acta, Part A.

[40] Piccinelli F., Melchior A., Speghini A., Monari M., Tolazzi M., Bettinelli M. (2013). Europium (III) Complexes with New
N-Donor Ligand: A
Comparative Study in Solid State and Solution. Polyhedron.

[41] Nunes S. C., De Zea Bermudez V., Cybinska J., Sá Ferreira R.
A., Carlos L. D., Legendziewicz J., Silva M. M., Smith M. J., Ostrovskii D. (2008). Structure
and Photoluminescence of Di-Amidosil Nanohybrids
Incorporating Europium Triflate. J. Alloys Compd..

[42] Bandgar B. P., Bettigeri S. V., Joshi N. S. (2004). Molecular Iodine Catalyzed Highly
Rapid Synthesis of 1,5-Benzodiazepine Derivatives Under Mild Conditions. Synth. Commun..

[43] Henderson R. K., Jiménez-González C., Constable D. J. C., Alston S. R., Inglis G. G. A., Fisher G., Sherwood J., Binks S. P., Curzons A. D. (2011). Expanding GSK’s Solvent Selection
Guide – Embedding Sustainability into Solvent Selection Starting
at Medicinal Chemistry. Green Chem..

[44] Cotton S. A., Raithby P. R., Schiffers S., Teat S. J., Warren J. E. (2022). The Synthesis
and Structure of a Scandium Nitrate Hydroxy-Bridged Dimeric Complex
Supported by Bipyridyl Ligands. Molecules.

[45] Lindqvist-Reis P., Persson I., Sandström M. (2006). The Hydration
of the Scandium­(iii)
Ion in Aqueous Solution and Crystalline Hydrates Studied by XAFS Spectroscopy,
Large-Angle X-Ray Scattering and Crystallography. Dalton Trans..

[46] Aldrich K. E., Popov I. A., Root H. D., Batista E. R., Greer S. M., Kozimor S. A., Lilley L. M., Livshits M. Y., Mocko V., Janicke M. T., Scott B. L., Stein B. W., Yang P. S. (2022). Solid-State,
Solution, and Theoretical Characterization of an “in-Cage”
Scandium-NOTA Complex. Dalton Trans..

[47] Elgohary A. M., Rafat E.-H., Alhunayhin S. M., Al-Ghamdi Y. O., El-Banna M. G., Rizk S. A. (2025). Microwave (MW) and
Ultrasound-Ultraviolet
(US/UV) Energy-Assisted Green Synthesis of Spiro Heterocycles for
High-Yield Efficiency and Agrochemical Applications. Results Chem..

[48] Xia H., Ahmad M. A., Guo J., Yang Y. (2023). Unraveling the Roles
of MW/UV/TiO2 Photocatalysis Technologies for Organic Wastewater Treatment. Catalysts.

[49] Du J., Skubi K. L., Schultz D. M., Yoon T. P. (2014). A Dual-Catalysis
Approach to Enantioselective [2 + 2] Photocycloadditions Using Visible
Light. Science.

[50] Yada H., Tanaka J., Nagakura S. (1960). Charge-Transfer Complexes
between
Iodine and Various Aliphatic Amines. Bull. Chem.
Soc. Jpn..

