# Supplementary material for: A Novel Integrated Microwave/UV/In Situ Fourier Transform Infrared Platform: Application to 1,5-Benzodiazepines Synthesis
Source: ACS Omega. 2026 Sep 30;11(40):61646–61. doi: 10.1021/acsomega.6c07314 (PMC13670338; doi:10.1021/acsomega.6c07314)

Supplementary Material

# A Novel Integrated MW/UV/in situ FTIR Platform: Application to 1,5-Benzodiazepines Synthesis

Alina Cherniienko [a, b*], Kacper Kossakowski [a,b], Lucjusz Zaprutko [a], Roman Lesyk [c,d], Anna Pawełczyk [a]

[a] *Poznan University of Medical Sciences, Department and Chair of Organic Chemistry, Poznan, Poland, https://ror.org/02zbb2597*
[b] *Poznan University of Medical Sciences, Doctoral School of Poznan University of Medical Sciences, Poznan, Poland, https://ror.org/02zbb2597*
[c] *Danylo Halytsky Lviv National Medical University, Department of Pharmaceutical, Organic and Bioorganic Chemistry, Lviv, Ukraine*
[d] *University of Information Technology and Management in Rzeszow, Department of Biotechnology and Cell Biology, Rzeszow, Poland*

[*] Corresponding author.
*E-mail address*: alina.cherniienko@student.ump.edu.pl (A. Cherniienko)

**List of contents:**


1. *Ex situ* ATR-FTIR/NMR/MS spectra of 1,5-benzodiazepine derivatives synthesized…………………………......**2**
2. *In situ* FTIR of the synthesis process of 1,5-benzodiazepine derivatives synthesized……………………….……..**14**
3. Kinetic profiles of 2,4-Didiethyl-2-methyl-2,3-dihydro-1H-1,5-benzodiazepine, 2,2,4,8-Tetramethyl-2,3-dihydro-1H-1,5-benzodiazepine and 2-Methyl-2,4-diphenyl-2,3-dihydro-1H-1,5-benzodiazepine…………..**17**
4. Experimental setup: details, solvent evaporation tests, Montmorillonite K10 fouling FTIR imaging……...…**18**


## 1. *Ex situ* ATR-FTIR/NMR/MS spectra of products:

### 1.1. 2,2,4-trimethyl-2,3-dihydro-1H-1,5-benzodiazepine

**Figure S1**. *Ex situ* FTIR spectrum of 2,2,4-trimethyl-2,3-dihydro-1H-1,5-benzodiazepine:

ATR-FTIR $cm^{-1}$: 3292 (m), 2965 (w), 1633 (m), 1474 (s), 1223 (m), 767 (s).

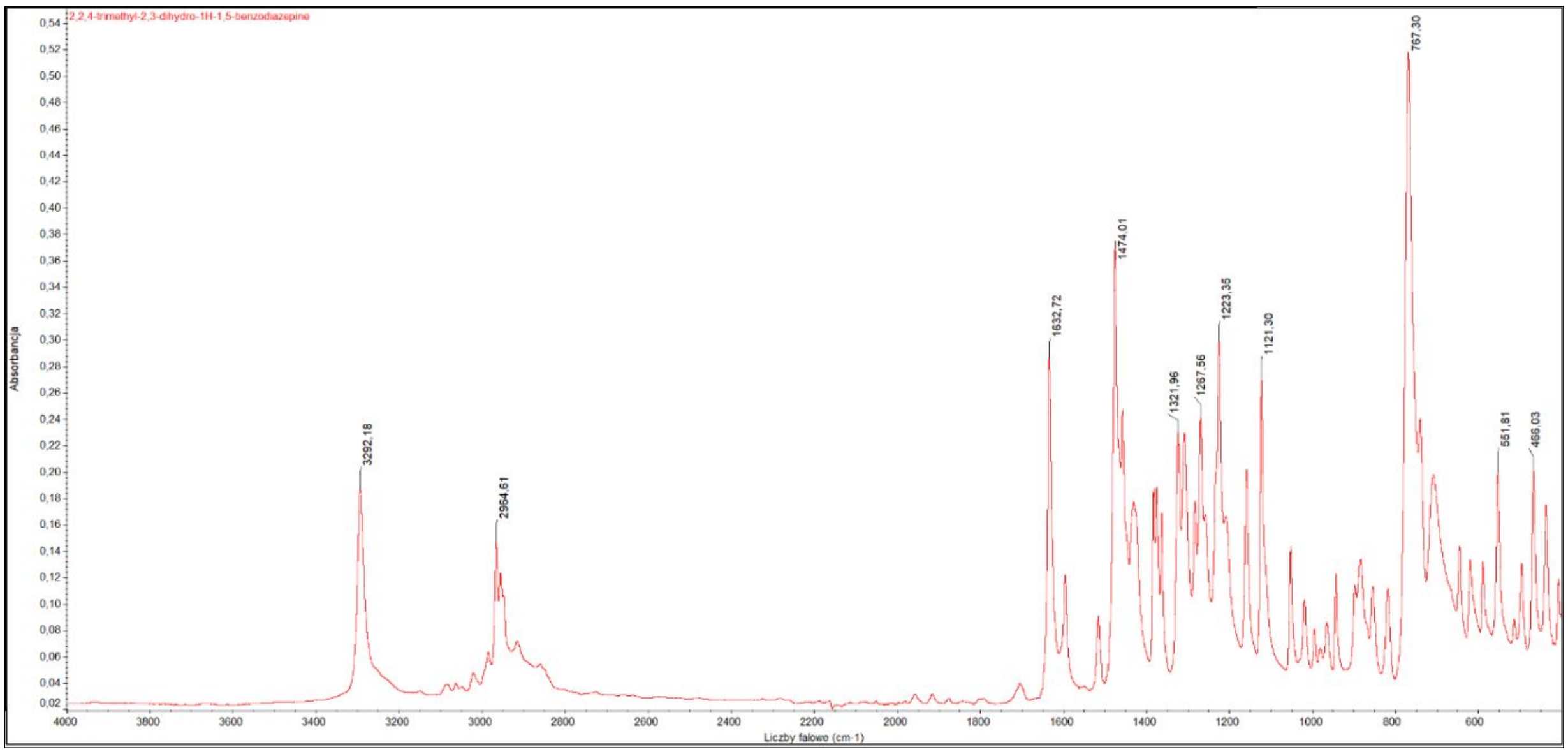


**Table S1.** 2,2,4-trimethyl-2,3-dihydro-1H-1,5-benzodiazepine *ex situ* FTIR spectrum peaks description

| Wavenumber (cm⁻¹) | Intensity / Shape | Assignment | Remarks / Interpretation |
|---|---|---|---|
| 3292 | sharp, strong | N–H stretching | In 1,5-benzodiazepines (1,5-BD) containing an N–H group, this absorption appears at 3300–3250 cm⁻¹. The sharpness suggests a largely non-hydrogen-bonded N–H stretch (consistent with an intramolecularly constrained or sterically hindered environment). |
| 2964 | sharp, medium, wide shoulders | aliphatic C–H stretching | The absorption near 2960 cm⁻¹ (with twin peaks or shoulders) arises from overlapping asymmetric (~2960 cm⁻¹) and symmetric (~2870–2940 cm⁻¹) C–H stretching modes of methyl and methylene groups. Such features are typical for aliphatic systems with several methyl groups attached to nitrogen or tertiary carbon atoms. |
| 1632 | sharp, strong | C=N (imine/Schiff-type) stretching | Bands in the 1600–1650 cm⁻¹ region are typical of C=N stretching or conjugated ring vibrations in 1,5-BD systems. |
| 1474 | sharp, strong | C-H bending (methyl deformation) and/or aromatic C=C in-plane skeletal vibration | Bands near 1470–1460 cm⁻¹ are commonly due to methyl asymmetric bending ($\delta$_as $CH_3$) or methylene scissoring; in aromatic/heteroaromatic systems, a mixed C–C (ring) skeletal vibration can also contribute. The sharpness favors a fairly localized deformation mode (e.g., methyl scissoring from the 2,2,4-trimethyl substituents). |
| 1380, 1360 | medium, sharp | gem-dimethyl doublet | Symmetric bending vibration (the "umbrella" mode) of two methyl groups attached to the same carbon at position 2. |
| 1372 | medium, sharp | isolated methyl on position 4 | |

| | | | |
|---|---|---|---|
| 1267 | sharp, with broad shoulders | aromatic C–N stretching and in-plane N–H deformation | The 1250–1350 cm⁻¹ range corresponds to aromatic C–N stretches and in-plane N–H bending. A contribution from ring C–C stretching cannot be excluded. The presence of “broad shoulders” suggests multiple overlapping modes: maybe C–N stretching of the heterocyclic ring + aromatic C–H in-plane bending. For many 1,5-BD, the C–N stretch is in ~1250-1300 cm⁻¹. |
| 1223 | sharp, strong | C–N stretching and aromatic C–H in-plane bending | The 1200–1250 cm⁻¹ region commonly hosts C–N stretching components and in-plane CH bending in substituted aromatics. Multiple alkyl and heteroatom substituents produce several closely spaced bands in this zone, so 1223 cm⁻¹ is plausibly a mixed mode. |
| 1121 | sharp, strong | C–N stretch (aliphatic/arylic C–N) and ring skeletal (C–C/C–N) vibrations | Bands in the 1100–1050 cm⁻¹ region are characteristic of C–N stretches and ring breathing/rocking modes. A strong, relatively sharp feature at ~1120 cm⁻¹ is consistent with an identifiable C–N component. |
| 767 | sharp, strong | aromatic C–H out-of-plane bending (benzene ring substitution pattern) | Bands between 770-700 cm⁻¹ indicate aromatic C–H out-of-plane bends. A strong feature at ~767 cm⁻¹ is diagnostic of ortho- or adjacent-substituted benzene rings. Given that the 1,5-BD has a benzene ring fused in the heterocycle, this is consistent with the aromatic substitution pattern. |

**Figure S2-3**. NMR spectrum of 2,2,4-trimethyl-2,3-dihydro-1H-1,5-benzodiazepine:

**$^1$H-NMR ($CDCl_3$, 400 MHz):** $\delta$ 7.17 – 7.08 (m, 1H), 7.03 – 6.93 (m, 2H), 6.77 – 6.68 (m, 1H), 2.96 (s, 1H), 2.36 (s, 3H), 2.22 (s, 2H), 1.34 (s, 6H).

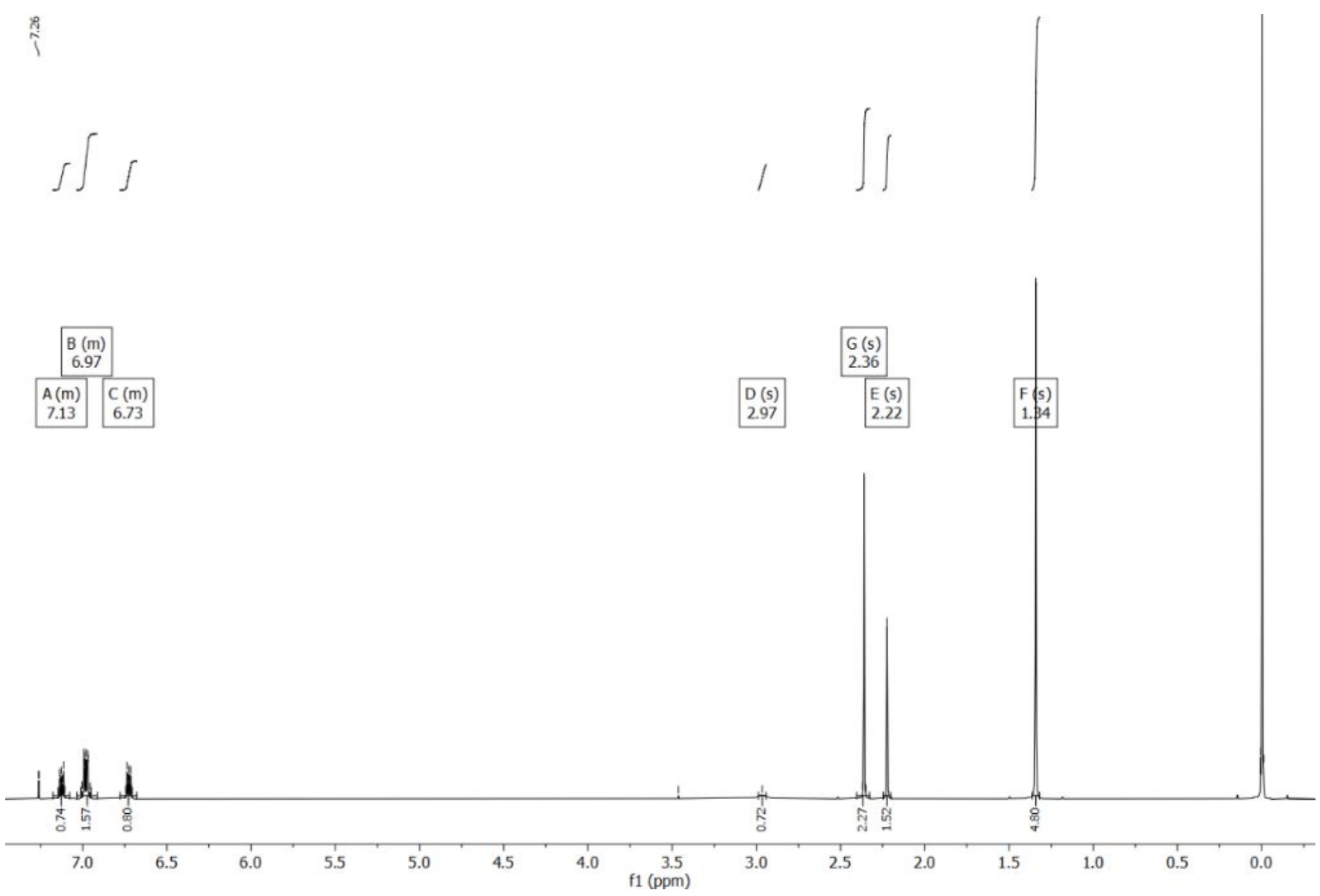


**$^{13}$C-NMR ($CDCl_3$, 101 MHz):** $\delta$ 172.52 (s, 1C), 140.95 (s, 1C), 138.04 (s, 1C), 126.99 (s, 1C), 125.64 (s, 1C), 122.25 (s, 1C), 121.88 (s, 1C), 68.54 (s, 1C), 45.24 (s, 1C), 30.64 (s, 1C), 30.02 (s, 2C).

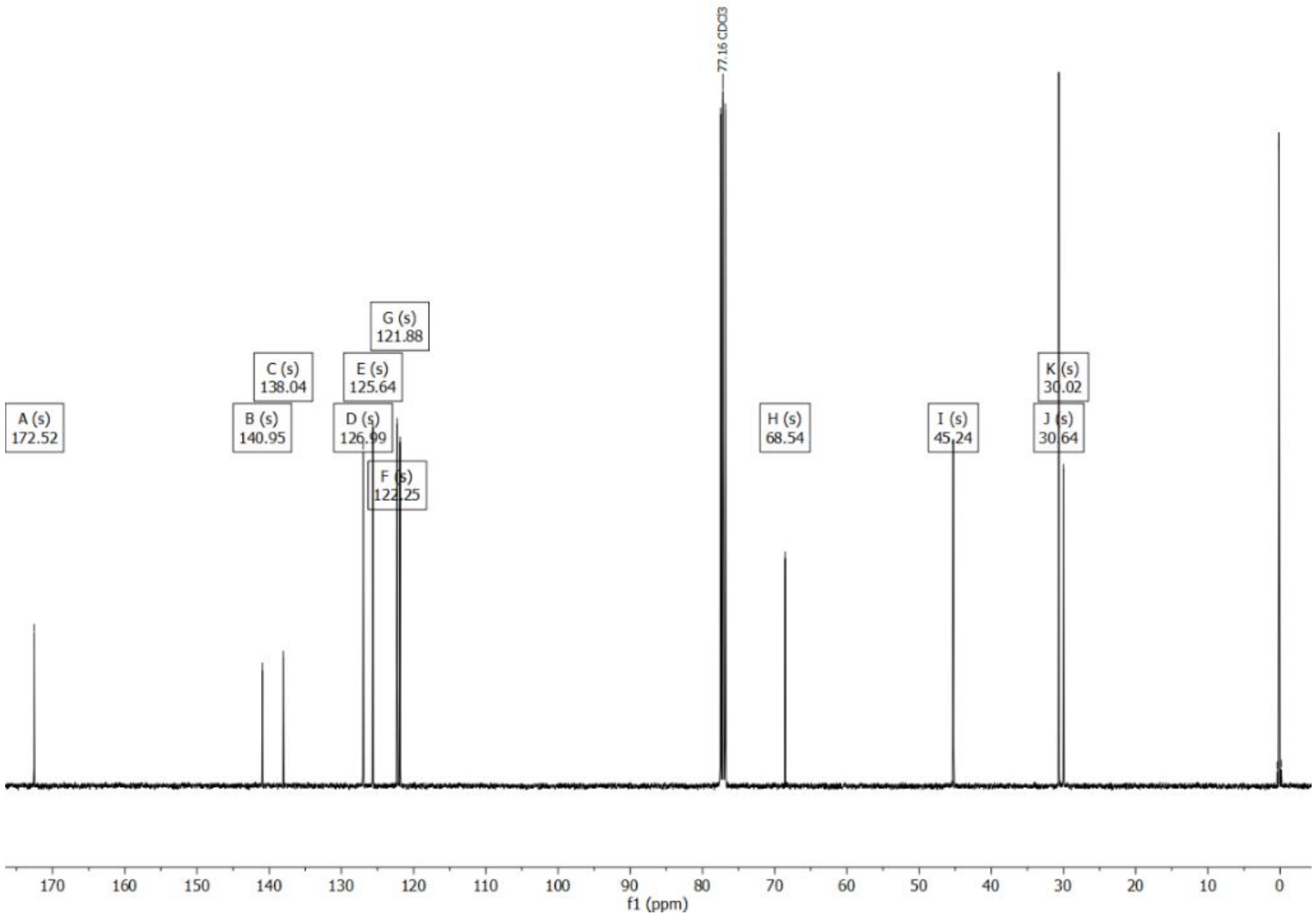


**Figure S4.** MS spectrum of 2,2,4-trimethyl-2,3-dihydro-1H-1,5-benzodiazepine:

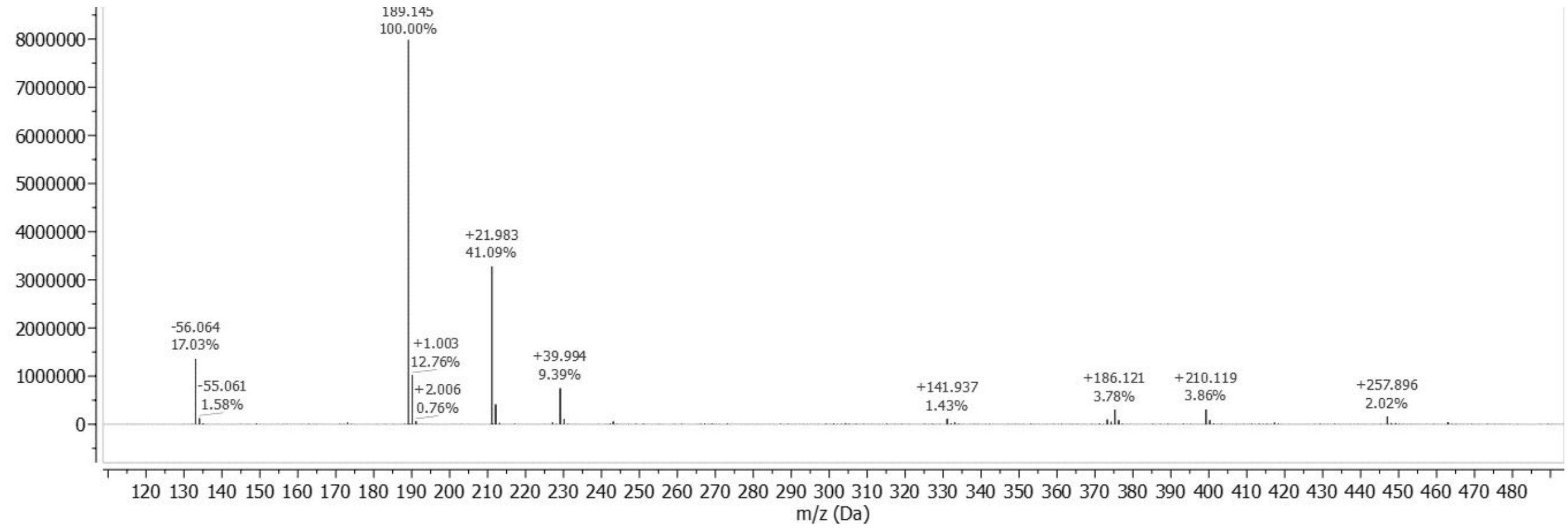


MS (ESI+): m/z 189.15 ($[M+H]^+$, 100%), 211.13 ($[M+Na]^+$, 41%), 133.08 ($[M–C_4H_8+H]^+$, 17%).

### 1.2. 2,4-Dimethyl-3H-1,5-benzodiazepine

**Figure S5.** *Ex situ* FTIR spectrum of 2,4-Dimethyl-3H-1,5-benzodiazepine:

ATR-FTIR $cm^{-1}$: 3058 (w), 2978 (w), 2938 (w), 1632 (m), 1456 (m), 1373 (m), 778 (s).

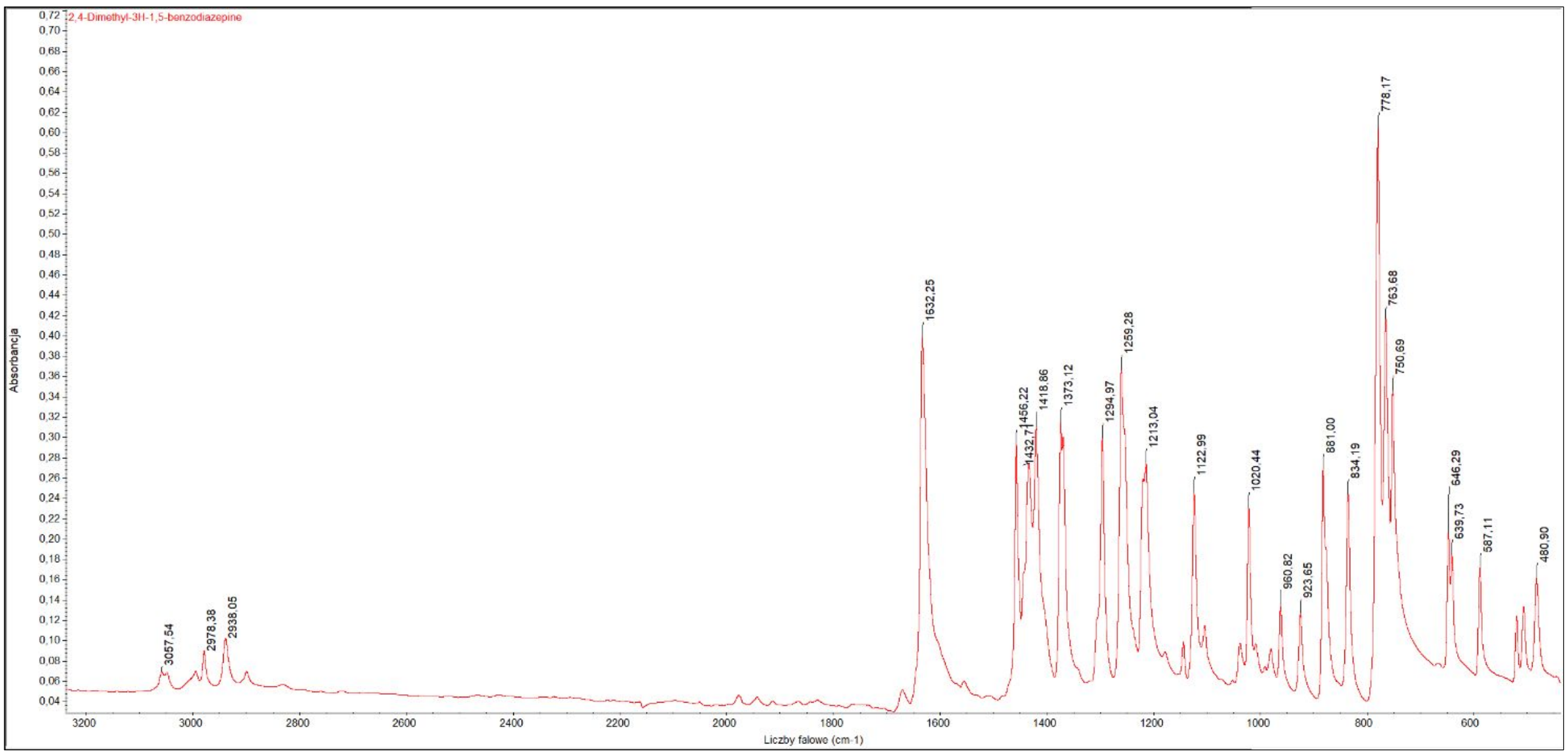

**Table S2**. 2,4-Dimethyl-3H-1,5-benzodiazepine *ex situ* FTIR spectrum peaks description

| Wavenumber ($cm^{-1}$) | Intensity / Shape | Assignment | Remarks / Interpretation |
|---|---|---|---|
| 3057 | weak | aromatic C-H stretch | Indicates $sp^2$ hybridized carbons attached to hydrogen (hydrogens on benzene ring). |
| 2978, 2938 | weak | aliphatic C-H stretches | Corresponds to $sp^3$ hybridized carbons. Asymmetric and symmetric stretching of the hydrogens on two CH3 groups and the single -CH2- group at the 3-position of the ring. |
| 1632 | sharp, strong | C=N (imine/Schiff-type) stretching expected in 1,5-γγ systems | |
| 1456, 1432, 1419 | sharp, strong | aromatic C=C stretching | Aromatic rings typically show a series of peaks in the 1600–1400 $cm^{-1}$ range due to the complex stretching of the carbon skeleton. Also, 1456 $cm^{-1}$ could correspond to asymmetric C-H bending (scissoring) of methyl and methylene groups. |
| 1295, 1259 | sharp, strong | C-N single bond stretching | |
| 1213, 1123, 1020, and 881, 834 | sharp, medium | in-plane and out-of-plane C-H bending vibrations on the aromatic ring | |
| 778, 763, 750 | sharp, strong | *ortho*-disubstituted benzene ring | It represents the out-of-plane bending of the four adjacent aromatic hydrogens. |

**Figure S6-7**. NMR spectrum of 2,4-Dimethyl-3H-1,5-benzodiazepine:

$^{1}$H-NMR ($CDCl_3$, 400 MHz): $\delta$ 7.41 – 7.32 (m, 2H), 7.22 (dd, J = 6.1, 3.5 Hz, 2H), 2.82 (br s, 2H), 2.35 (s, 6H).

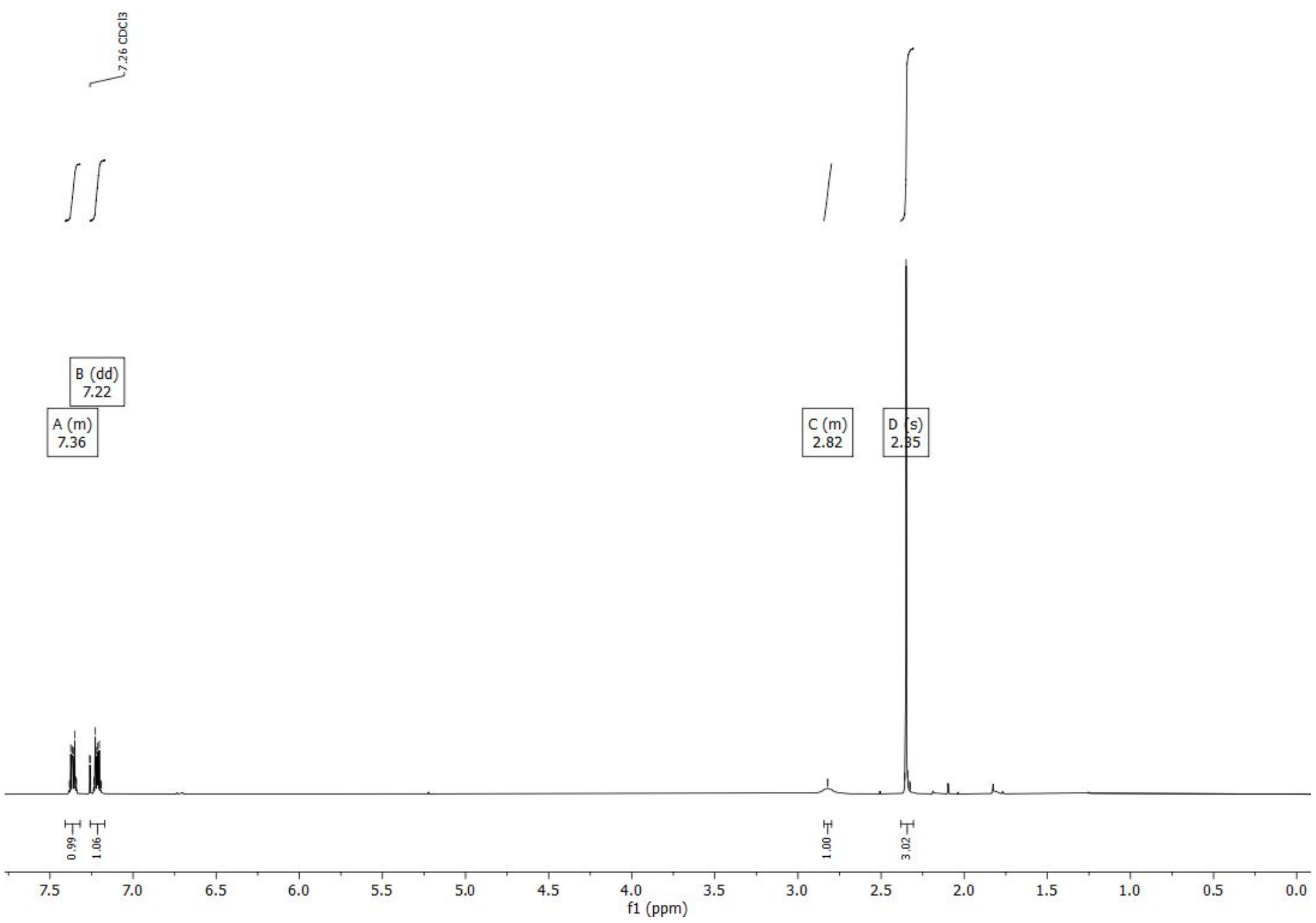


$^{13}$C-NMR ($CDCl_3$, 101 MHz): $\delta$ 157.88 (s, 2C), 140.39 (s, 2C), 127.64 (s, 2C), 125.04 (s, 2C), 43.35 (s, 1C), 27.81 (s, 2C).

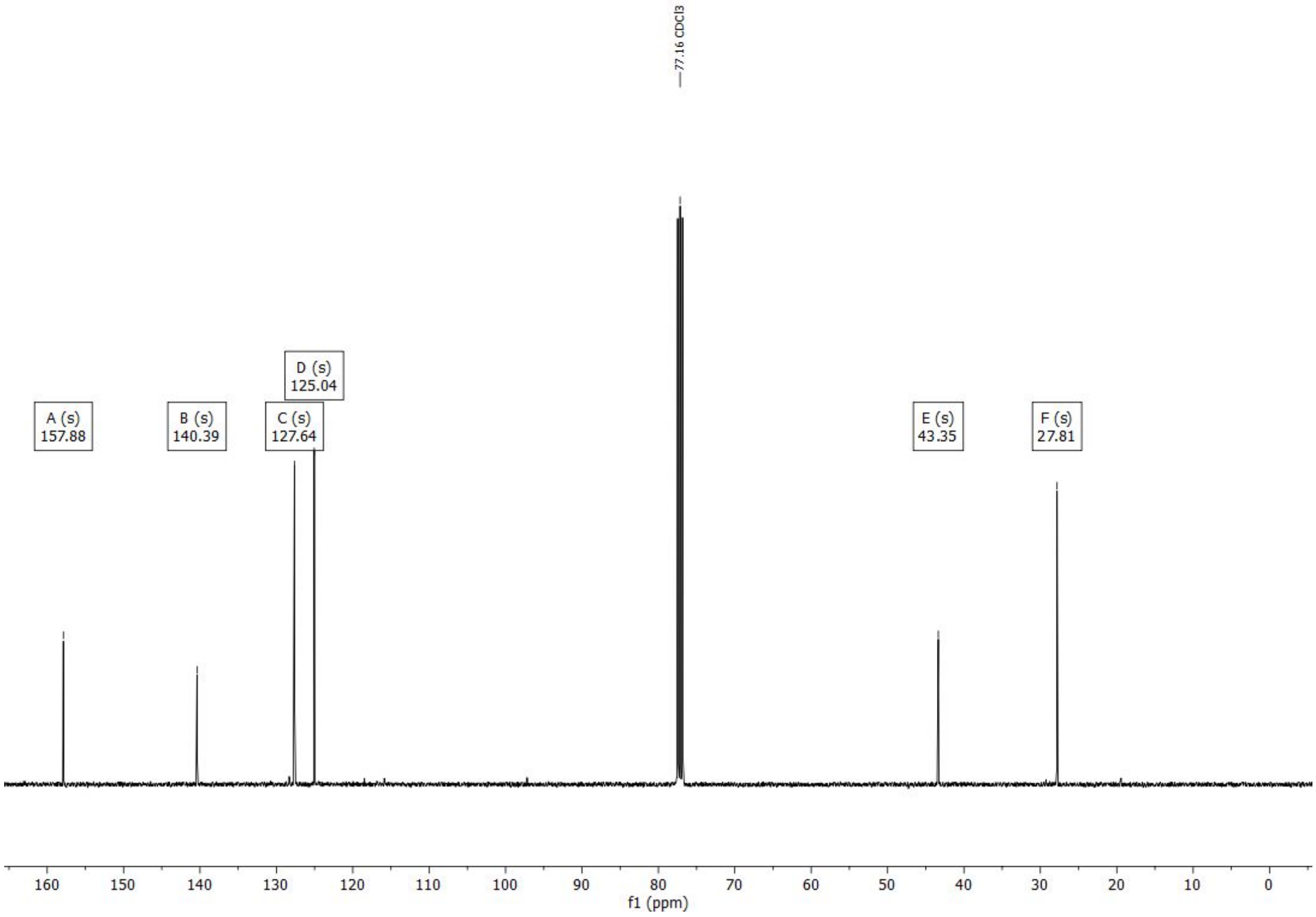


**Figure S8.** MS spectrum of 2,4-Dimethyl-3H-1,5-benzodiazepine

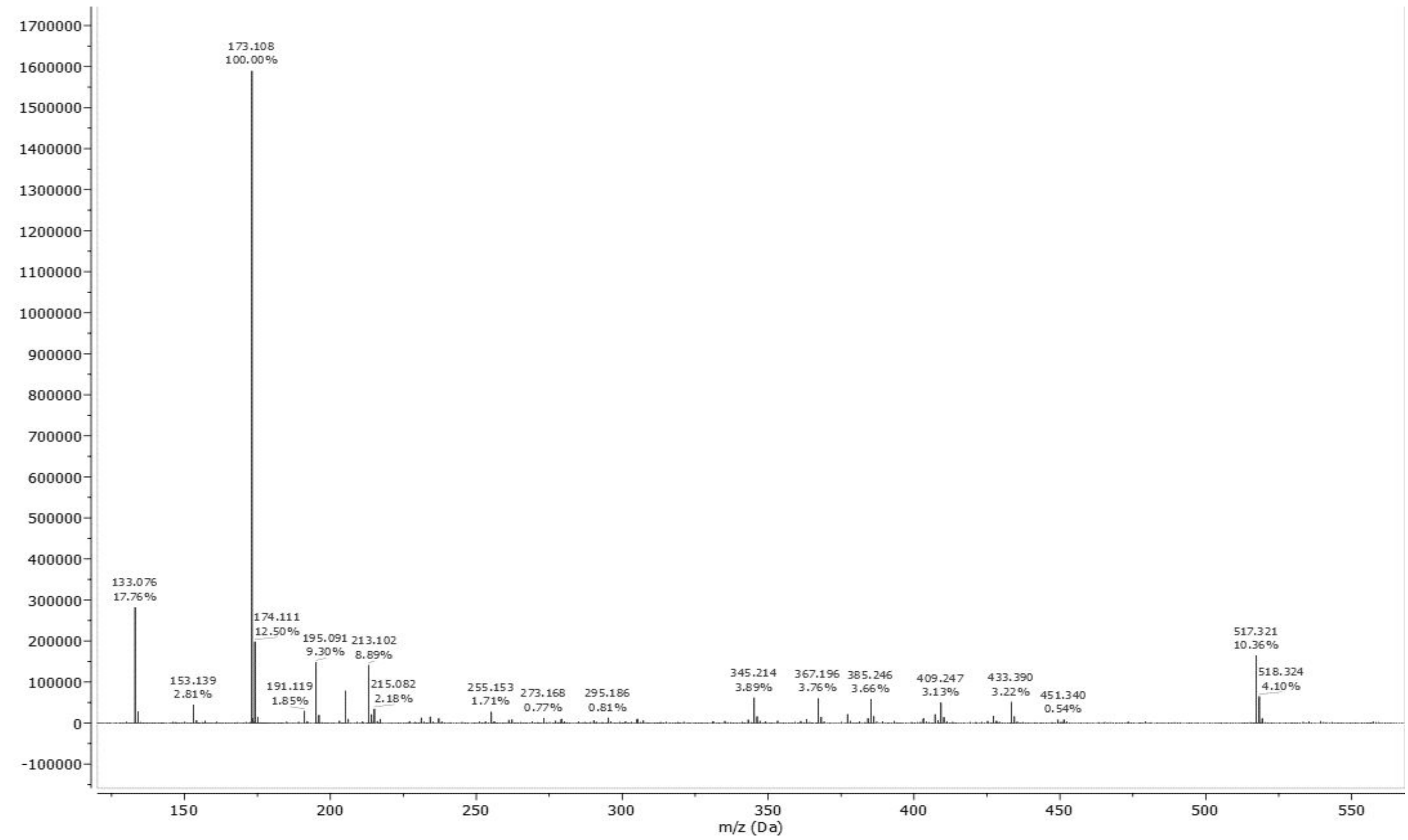


MS (ESI+): m/z 173.108 ($[M+H]^+$, 100%), 195.09 ($[M+Na]^+$, 9%), 345.22 ($[2M+H]^+$, 3.9%), 367.2 ($[2M+Na]^+$, 3.8%), 517.321 ($[3M+H]^+$, 10.36%).

### 1.3. 2,4-diethyl-2-methyl-2,3-dihydro-1H-1,5-benzodiazepine

**Figure S9.** *Ex situ* FTIR spectrum of 2,4-diethyl-2-methyl-2,3-dihydro-1H-1,5-benzodiazepine:

ATR-FTIR $cm^{-1}$: 3330 (w), 2965 (m), 1637 (m), 1471 (m), 1375 (w), 746 (s).

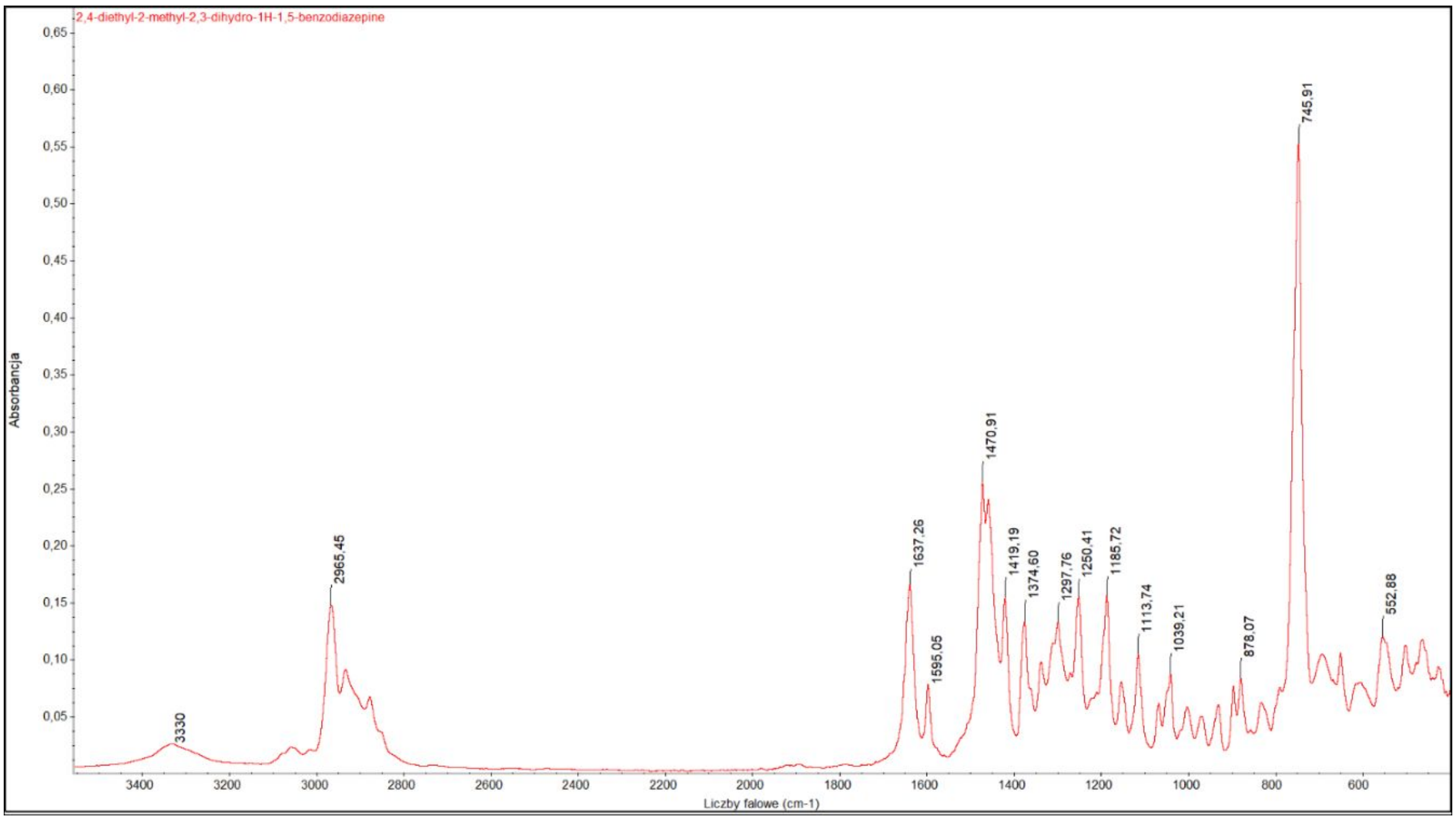


**Table S3.** 2,4-diethyl-2-methyl-2,3-dihydro-1H-1,5-benzodiazepine *ex situ* FTIR spectrum peaks description

| Wavenumber ($cm^{-1}$) | Intensity / Shape | Assignment | Remarks / Interpretation |
|---|---|---|---|
| 3330 | weak | N–H stretching | |
| 2965, 2935, 2877 | sharp, medium | aliphatic C–H stretching | Alkyl C–H stretching (asymmetric and symmetric stretches of $CH_2$ and $CH_3$) — consistent with the 2,4-diethyl and 2-methyl substituents. |
| 1637 | sharp, medium | C=N (imine/Schiff-type) stretching  expected in benzodiazepine systems | |
| 1595 | sharp, strong | aromatic C=C skeletal stretching | |
| 1470, 1458, 1419 | sharp, medium/weak | $CH_2$/$CH_3$ bending | |
| 1347, 1250, 1185, 1113 | weak/medium | C-C–N stretches / aromatic in-plane deformations consistent with an N-containing heteroaromatic ring. | |
| 745 | sharp, strong | aromatic C–H out-of-plane bending (benzene ring substitution pattern) | Bands between 770-700 $cm^{-1}$ indicate aromatic C–H out-of-plane bends. A strong feature at ~745 $cm^{-1}$ is diagnostic of ortho- or adjacent-substituted benzene rings. Given that 1,5-BD has a benzene ring fused in the heterocycle, this is consistent with the aromatic substitution pattern. |

**Figure S10-11**. NMR spectrum of 2,4-diethyl-2-methyl-2,3-dihydro-1H-1,5-benzodiazepine:

$^1$H-NMR ($CDCl_3$, 400 MHz): *δ* 7.19 – 7.10 (m, 1H), 7.02 – 6.90 (m, 2H), 6.76 – 6.66 (m, 1H), 3.10 (s, 1H), 2.68 – 2.54 (m, 2H), 2.24 (d, J = 12.9 Hz, 1H), 2.15 (d, J = 12.9 Hz, 1H), 1.74 – 1.53 (m, 2H), 1.29 – 1.17 (m, 6H), 0.95 (t, J = 7.5 Hz, 3H).

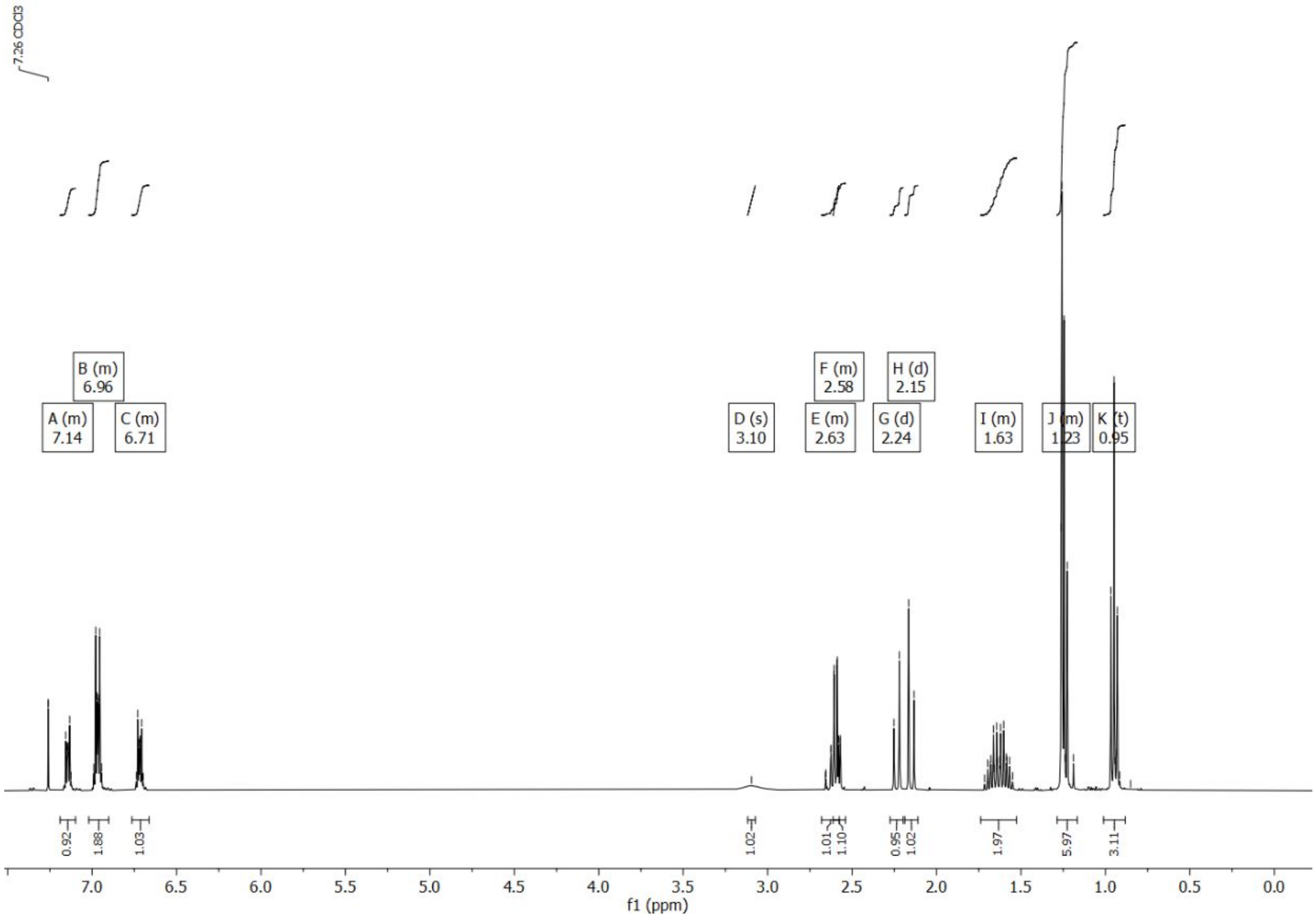


$^{13}$C-NMR ($CDCl_3$, 101 MHz): $\delta$ 176.01 (s, 1C), 140.98 (s, 1C), 138.05 (s, 1C), 127.20 (s, 1C), 125.53 (s, 1C), 121.97 (s, 1C), 121.89 (s, 1C), 70.90 (s, 1C), 42.27 (s, 1C), 35.84 (s, 1C), 35.80 (s, 1C), 27.06 (s, 1C), 10.77 (s, 1C), 8.69 (s, 1C).

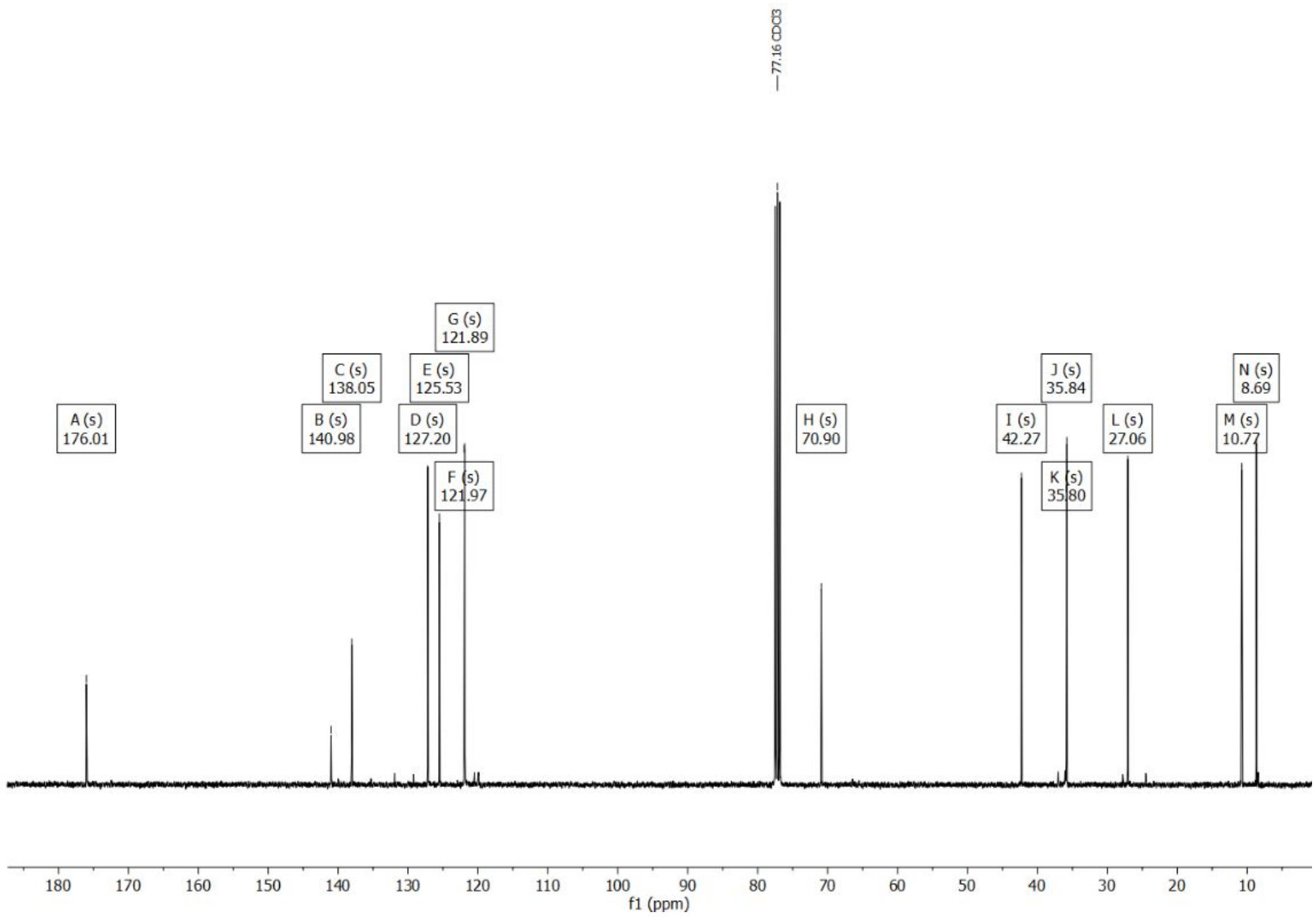


**Figure S12.** MS spectrum of 2,4-diethyl-2-methyl-2,3-dihydro-1H-1,5-benzodiazepine:

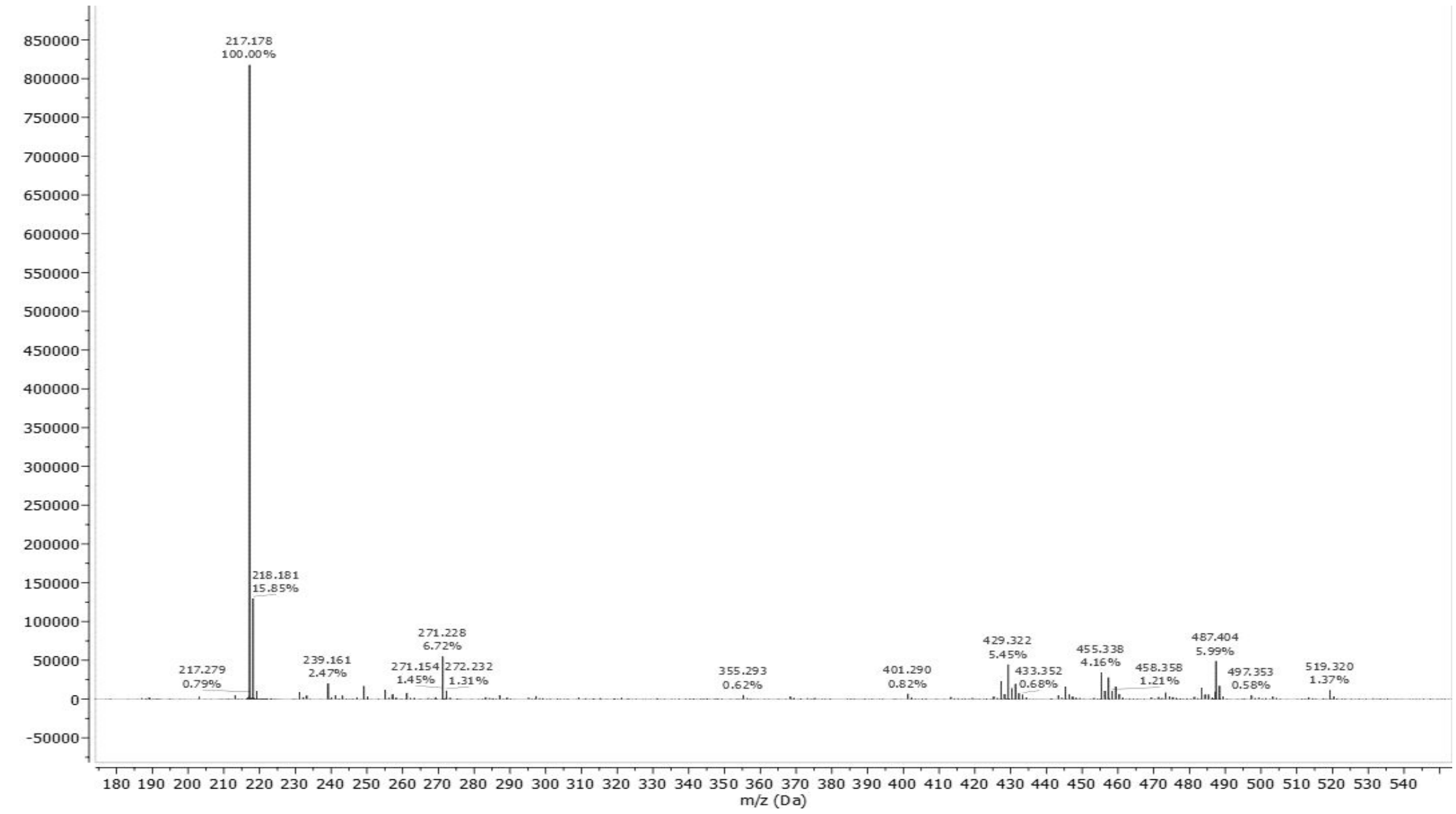

MS:(ESI+, m/z): 217.18 (100%) $[M+H]^+$, 218.18 (16%) $[M+1]^+$, 239.16 (2%) $[M+Na]^+$, 433.35 (<1%) [2M+H]+, 455.34 (4%) $[2M+Na]^+$.

### 1.4. 2,3-Dihydro-2,2,4,8-tetramethyl-(1H)-1,5-benzodiazepine

**Figure S13.** *Ex situ* FTIR spectrum of 2,3-Dihydro-2,2,4,8-tetramethyl-(1H)-1,5-benzodiazepine:

ATR-FTIR $cm^{-1}$: 3324 (w), 2960 (w), 1638 (m), 1475 (m), 1223 (m), 811 (m), 750 (s).

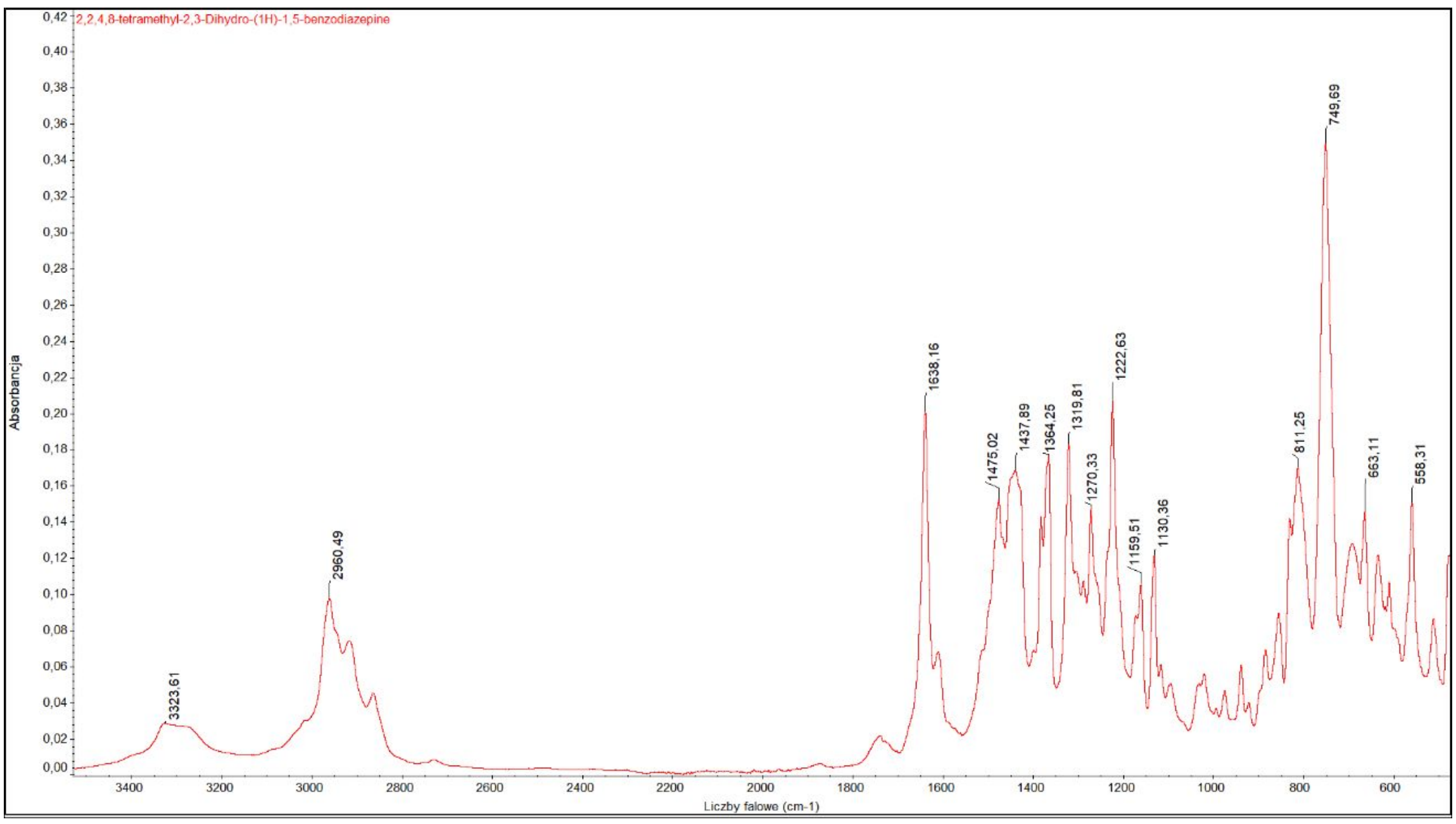

**Table S4**. 2,3-Dihydro-2,2,4,8-tetramethyl-(1H)-1,5-benzodiazepine *ex situ* FTIR spectrum peaks description

| Wavenumber ($cm^{-1}$) | Intensity / Shape | Assignment | Remarks / Interpretation |
|---|---|---|---|
| 3323 | weak, wide | N–H stretching | Characteristic of the secondary amine in the 1H-diazepine ring. |
| 2960, 2917 | medium, sharp | Aliphatic C–H stretching | Asymmetric and symmetric stretches of the $sp^3$ carbons. This perfectly reflects the four methyl groups (positions 2, 2, 4, and 8) and the single methylene (-CH2-) at position 3. |
| 1638 | strong, sharp | C=N stretching | |
| 1475, 1438 | strong, sharp, double-headed | C–H bending & Aromatic C=C stretching | Overlapping modes: asymmetric bending of methyl/methylene groups mixed with the in-plane skeletal vibrations of the aromatic ring. |
| 1364, 1382 | strong, sharp, doublet | Symmetric C–H bending (gem-dimethyl split) | 2,2-dimethyl grouping - the symmetric methyl "umbrella" mode mechanically couples and splits into this distinct doublet. |
| 1320 | strong, sharp | C–N stretching | |
| 1270 | medium, sharp | Aromatic C–N / C-H in-plane bending | |
| 1222 | strong, sharp | C–N stretching & C-C skeletal | Closely spaced bands in the 1200-1300 region are highly characteristic of multi-substituted nitrogen heterocycles. |
| 811 | wide, medium | Aromatic C–H out-of-plane (OOP) bending | Because there is a methyl at position 8, the benzene ring now has an isolated proton (at position 9). Isolated aromatic protons typically show an OOP bend right around 850–800 $cm^{-1}$. |
| 749 | strong, sharp | Aromatic C–H out-of-plane (OOP) bending | The other OOP bending mode for 2,2,4-trisubstituted benzene ring, representing the two adjacent hydrogens left on the ring. |

**Figure S14-15**. NMR spectrum of 2,3-Dihydro-2,2,4,8(7)-tetramethyl-(1H)-1,5-benzodiazepine (mixture of 7-methyl and 8-methyl isomers).

$^1$H-NMR ($CDCl_3$, 400 MHz): $\delta$ 7.03 – 6.94 (m, 1H), 6.84 – 6.74 (m, 1H), 6.66 (d, J = 8.1 Hz, 1H), 2.92 – 2.82 (m, 1H), 2.36 – 2.31 (m, 3H), 2.28 – 2.24 (m, 3H), 2.23 – 2.19 (m, 2H), 1.35 – 1.31 (m, 6H). Note: Spectrum represents an inseparable mixture of 7-methyl and 8-methyl isomers.

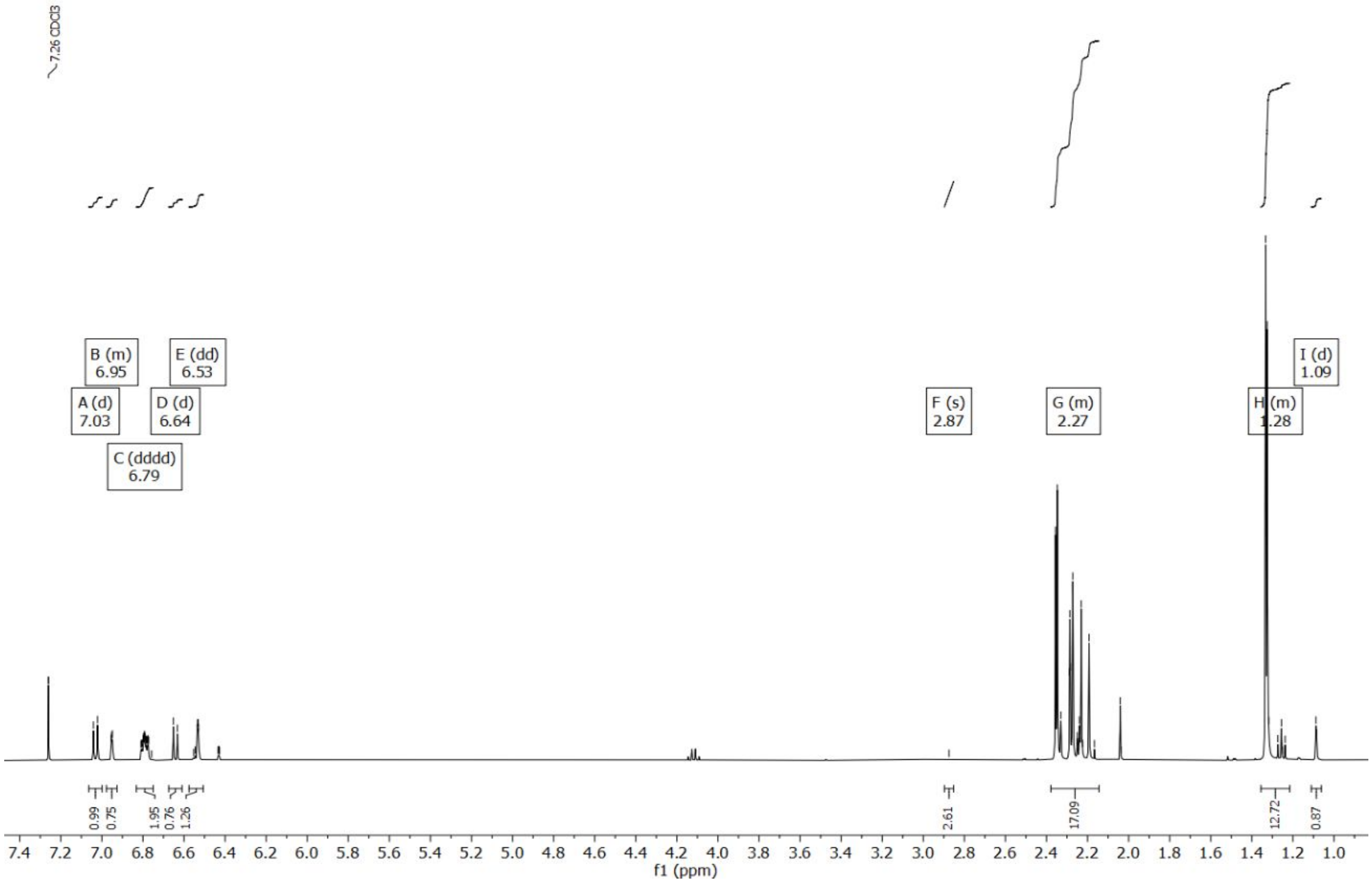


$^{13}$C-NMR ($CDCl_3$, 101 MHz): 172.78 (1C, s), 171.75 (1C, s), 141.14 (1C, s), 137.94 (1C, s), 135.52 (1C, s), 135.42 (1C, s), 131.97 (1C, s), 127.19 (1C, s), 127.10 (1C, s), 126.28 (1C, s), 124.76 (1C, s), 122.90 (1C, s), 122.17 (1C, s), 121.93 (1C, s), 68.62 (1C, s), 67.84 (1C, s), 45.44 (1C, s), 45.29 (1C, s), 30.71 (2C, s), 30.47 (2C, s), 29.97 (2C, s), 21.06 (1C, s), 20.77 (1C, s). Spectrum represents an inseparable mixture of 7-methyl and 8-methyl isomers.

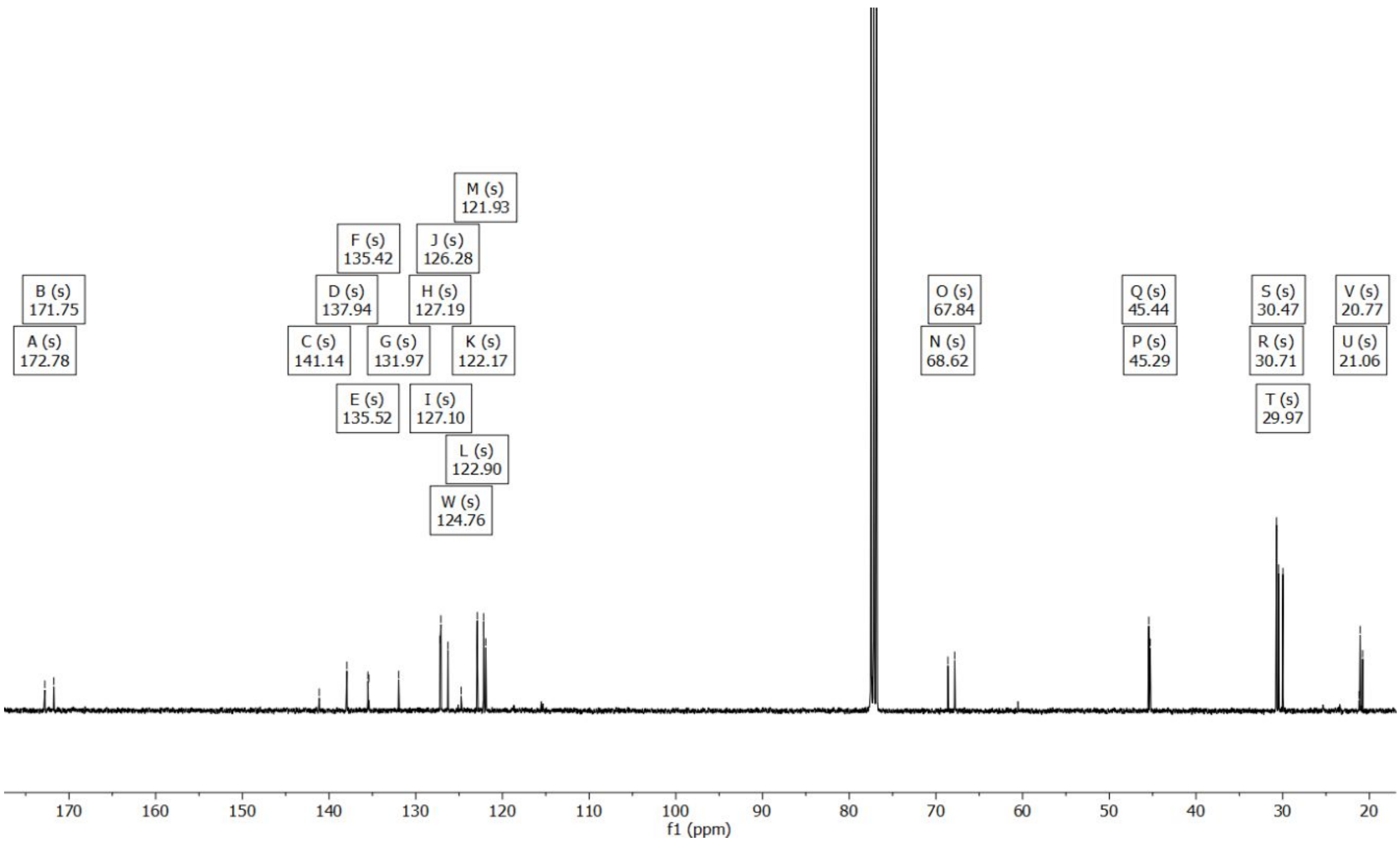


**Figure S16.** MS spectrum of 2,3-Dihydro-2,2,4,8-tetramethyl-(1H)-1,5-benzodiazepine

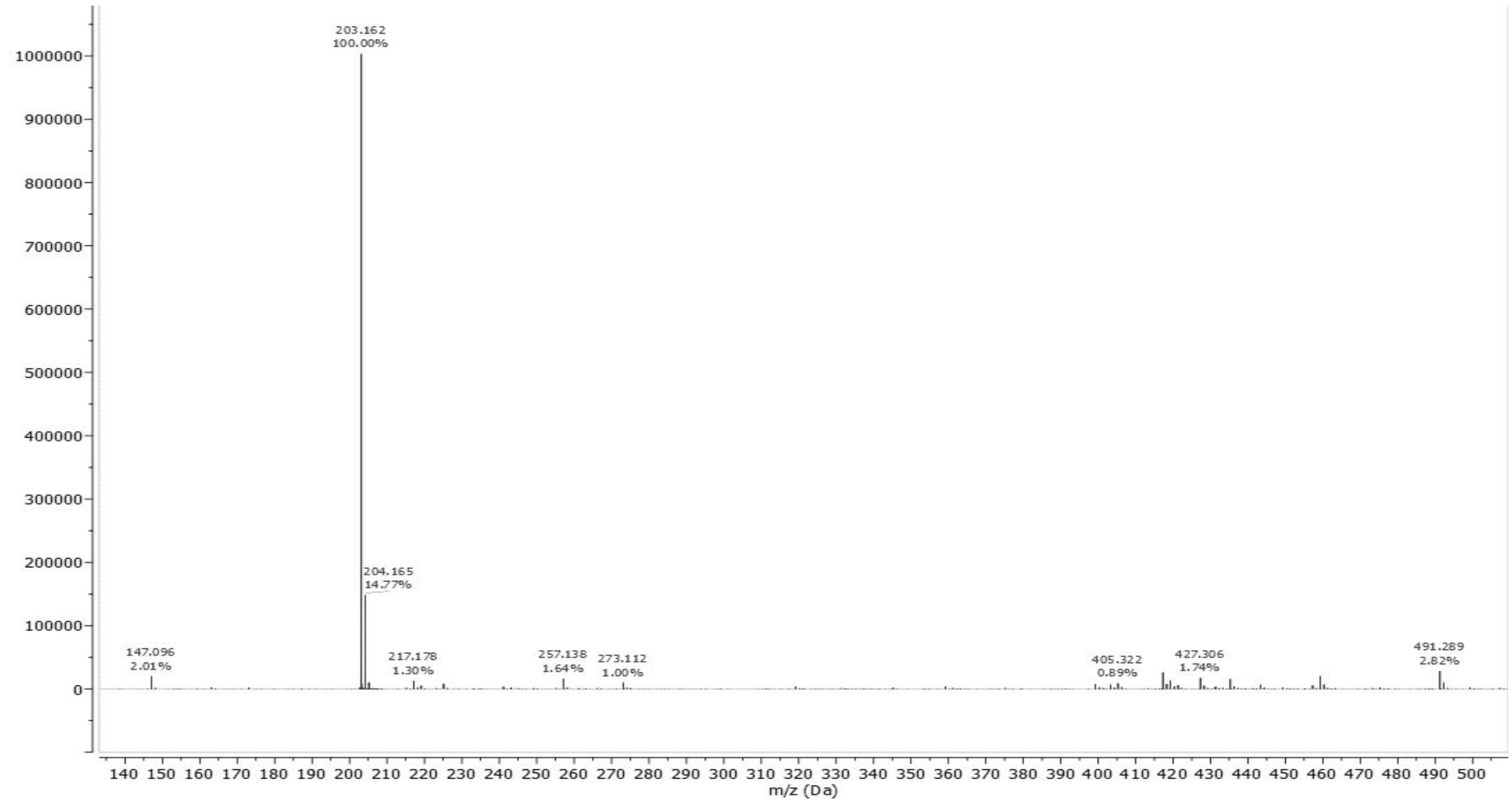


MS (ESI+): *m/z* 203.16 ($[M+H]^+$, 100%), 204.17 ($[M+1]^+$, 15%), 147.10 ($[M+H–C_4H_8]^+$, 2%), 405.32 ($[2M+H]^+$, 1%), 427.31 ($[2M+Na]^+$, 2%).

### 1.5. 2-Methyl-2,4-diphenyl-2,3-dihydro-1H-1,5-benzodiazepine

**Figure S17.** 2-methyl-2,4-diphenyl-2,3-dihydro-1H-1,5-benzodiazepine

ATR-FTIR cm$^{-1}$: 3275 (m), 3069 (w), 2974 (w), 1634 (w), 1597 (m), 1467 (m), 759 (s), 749 (s), 699 (s).

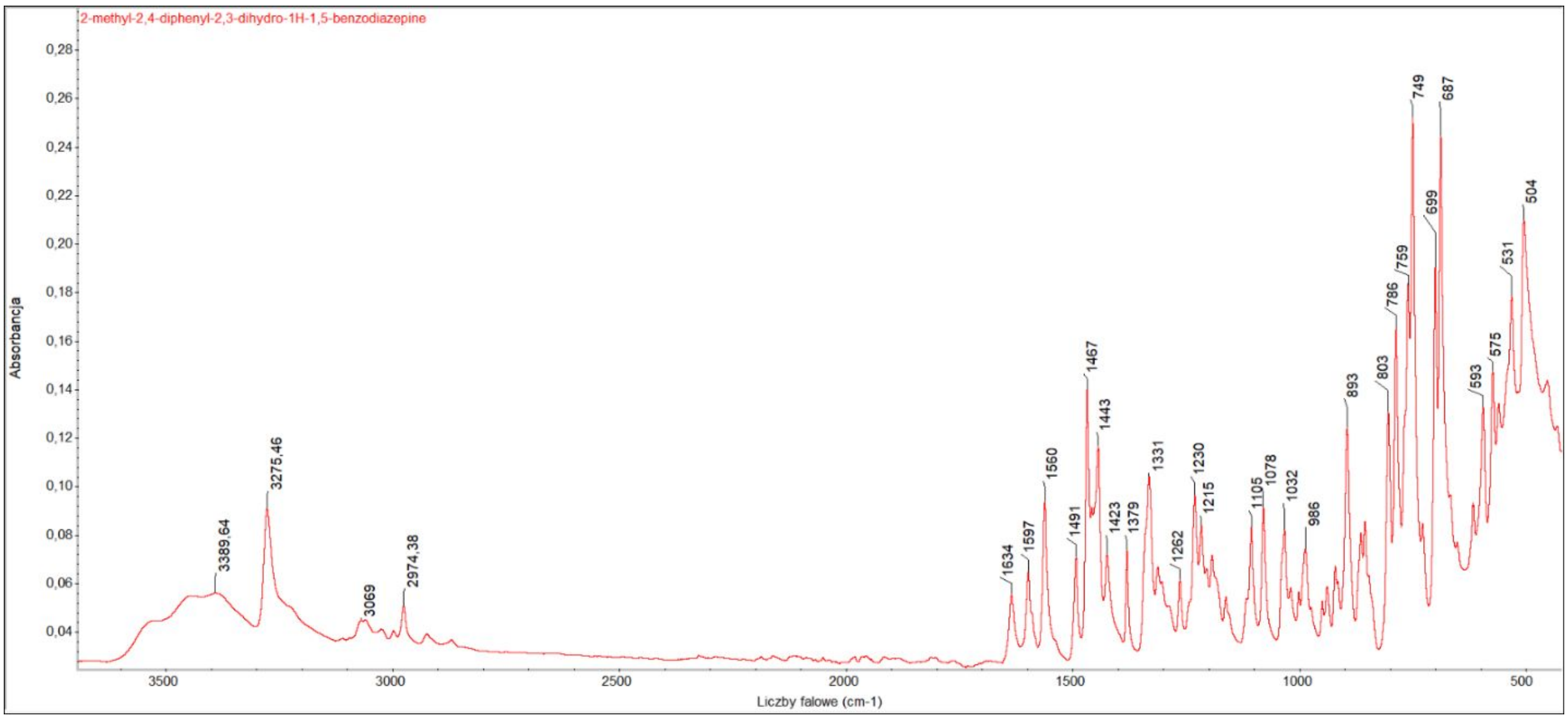


**Table S5.** 2,3-Dihydro-2,2,4,8-tetramethyl-(1H)-1,5-benzodiazepine *ex situ* FTIR spectrum peaks description

| Wavenumber (cm⁻¹) | Intensity / Shape | Assignment | Remarks / Interpretation |
|---|---|---|---|
| 3389, 3275 | Weak/medium, wide | N-H stretching | Distinctive of the secondary amine in 1,5-BD ring. The multiple peaks/breadth suggest slight hydrogen bonding. |
| 3069 | weak, sharp | Aromatic C-H stretching | Vibrations of the sp$^2$ carbons from the three phenyl rings (the fused ring and the two pendant groups). |
| 2974 | medium, sharp | Aliphatic C-H stretching | Stretching of sp$^3$ carbons. Corresponds to the single methyl group at position 2 and the $CH_2$-group at position 3. |
| 1634 | medium, sharp | C=N stretching | The "imine" stretch of the heptatomic ring; critical evidence of successful cyclization into the benzodiazepine core. |

| | | | |
|---|---|---|---|
| 1597, 1560 | medium, sharp | Aromatic C=C stretching | Skeletal vibrations of the benzene rings. The 1597 peak is often enhanced by conjugation with the C=N bond. |
| 1491, 1467, 1443 | strong, sharp | C-H bending & Aromatic C=C | Overlapping modes: methylene scissoring and methyl asymmetric bends mixed with aromatic skeletal vibrations. |
| 1379, 1331 | strong, sharp | Methyl C-H bending / C-N stretch | The 1379 $cm^{-1}$ peak is the symmetric "umbrella" mode of the methyl group. |
| 1262, 1230, 1215 | Medium/strong, sharp | C-N stretching | Multiple bands representing the various C-N bonds (both aromatic and aliphatic) in the heterocyclic system. |
| 893 | medium, sharp | Aromatic C-H OOP | Out-of-plane bending is typical for substituted aromatics. |
| 759, 749 | strong, sharp | Aromatic C-H OOP | Ortho-disubstituted pattern for the fused benzene ring and vibrations from the mono-substituted phenyl rings. |
| 699, 687 | strong, sharp | Aromatic C-H / Ring deformation | Classic "mono-substituted benzene" signature, confirming the presence of the two pendant phenyl groups. |

**Figure S18-19**. NMR spectrum of 2-methyl-2,4-diphenyl-2,3-dihydro-1H-1,5-benzodiazepine.

$^{1}$H-NMR ($CDCl_3$, 400 MHz): $\delta$ 7.67 – 7.58 (m, 4H), 7.40 – 7.16 (m, 7H), 7.16 – 7.03 (m, 2H), 6.91 – 6.84 (m, 1H), 3.56 (s, 1H), 3.18 (d, J = 13.2 Hz, 1H), 3.01 (d, J = 13.2 Hz, 1H), 1.79 (s, 3H).

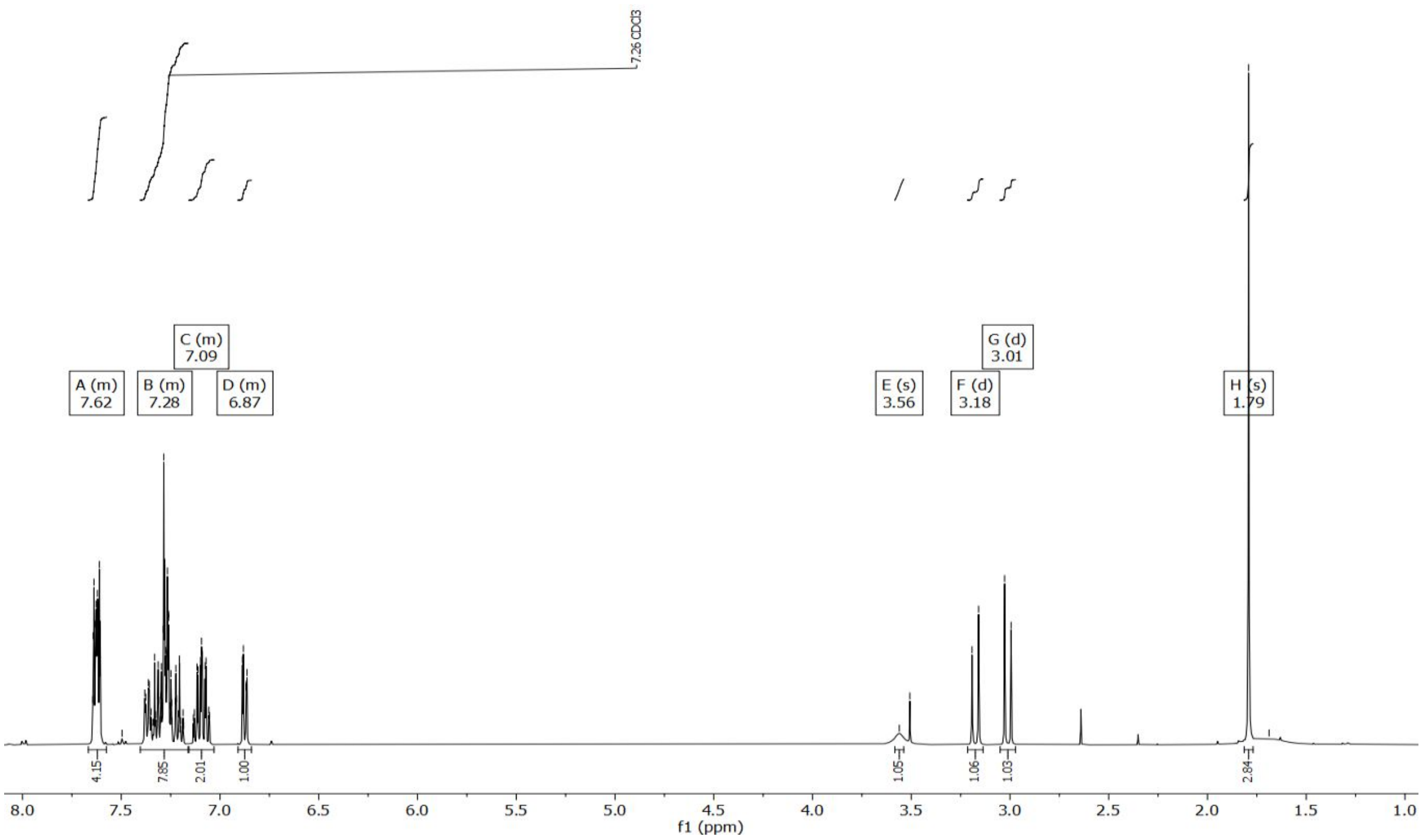


$^{13}$C-NMR ($CDCl_3$, 101 MHz): $\delta$ 167.88, 147.72, 140.11, 139.65, 138.23, 129.92, 128.76, 128.71, 128.45, 128.17, 127.24, 127.22, 126.52, 125.56, 121.79, 121.56, 73.84, 43.23, 30.02.

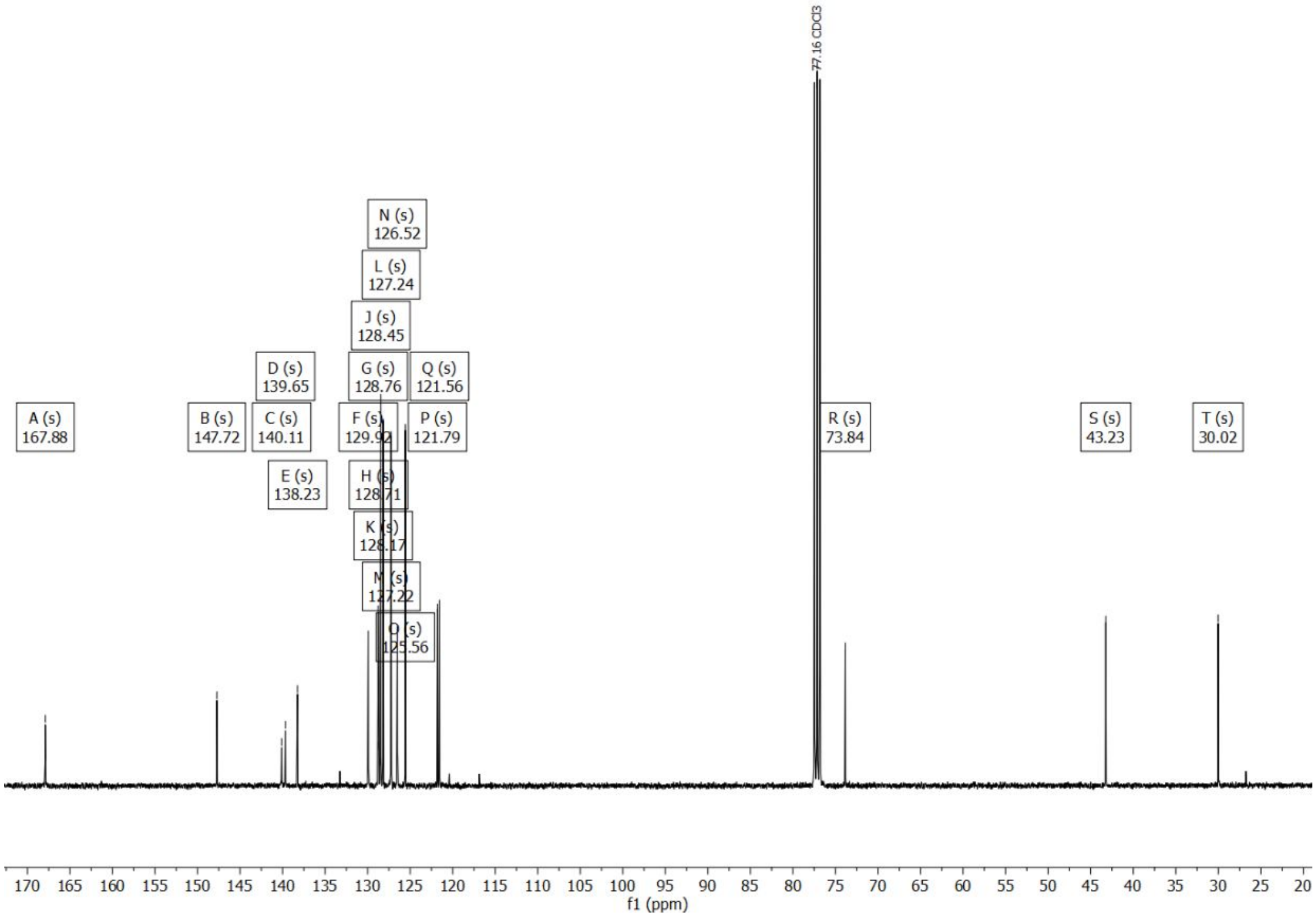


**Figure S20.** MS spectrum of 2-methyl-2,4-diphenyl-2,3-dihydro-1H-1,5-benzodiazepine.

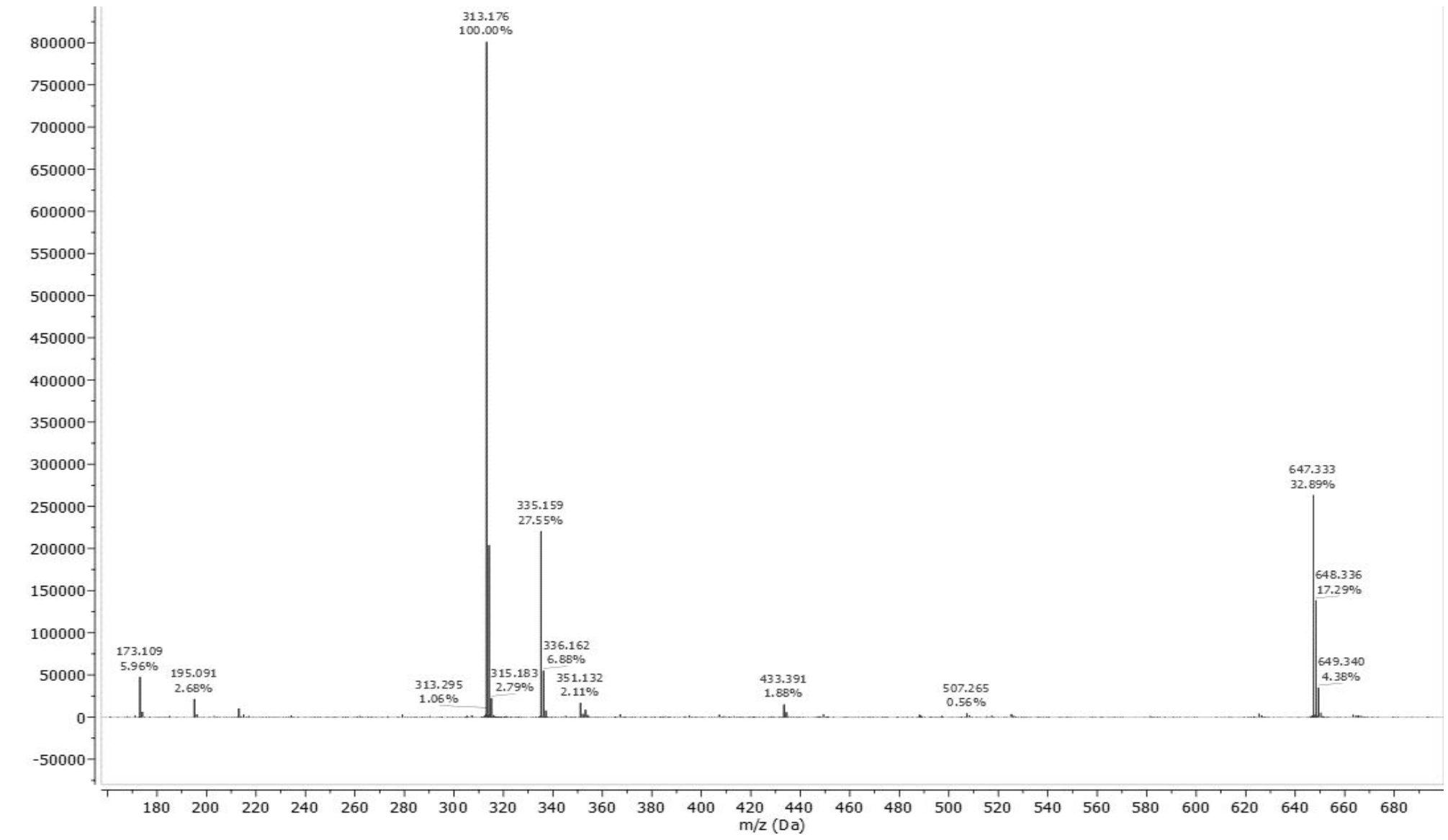


MS (ESI+): m/z 313.18 ($[M+H]^+$, 100%), 173.11 (Fragment, 6%), 335.16 ($[M+Na]^+$, 28%), 351.13 ($[M+K]^+$, 2%), 647.33 ($[2M+Na]^+$, 33%).

## 2. *In situ* FTIR

2.1. **2,4-Dimethyl-1H-1,5-benzodiazepine** (**2,4-Me-1,5-BD**):

**Figure S21-24.** *In situ* spectral comparison of peaks of substrates and product in MeOH and MeCN:

a) MeOH( full and partial IR spectra):

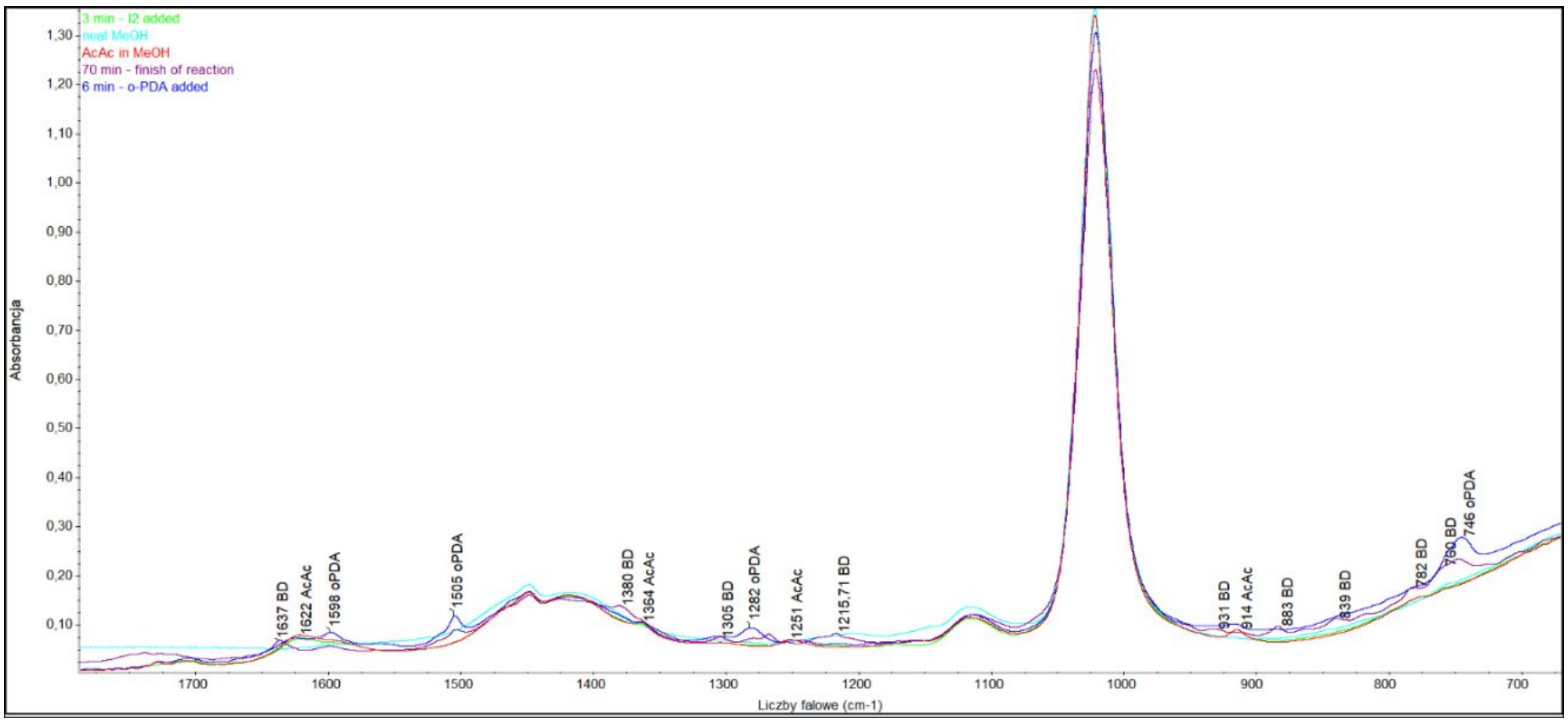


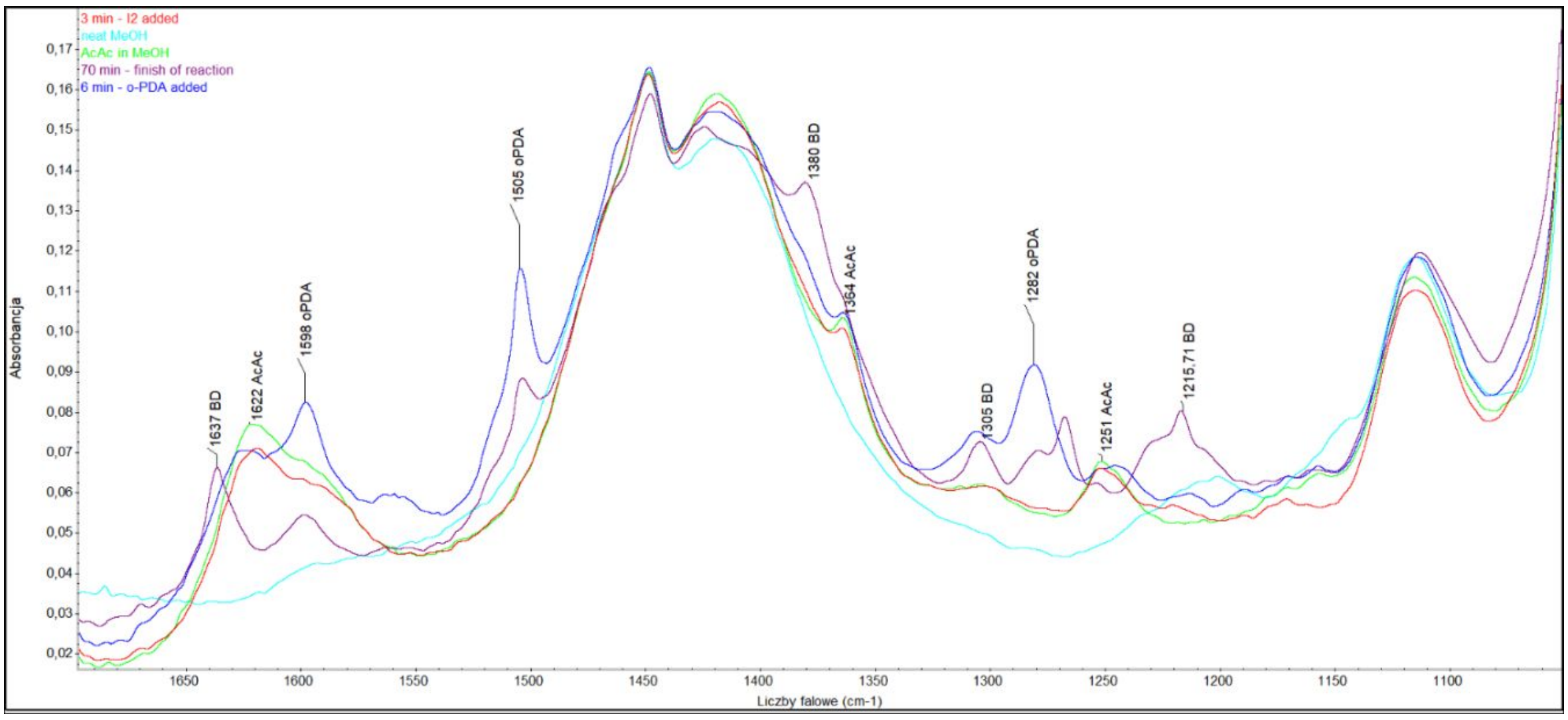


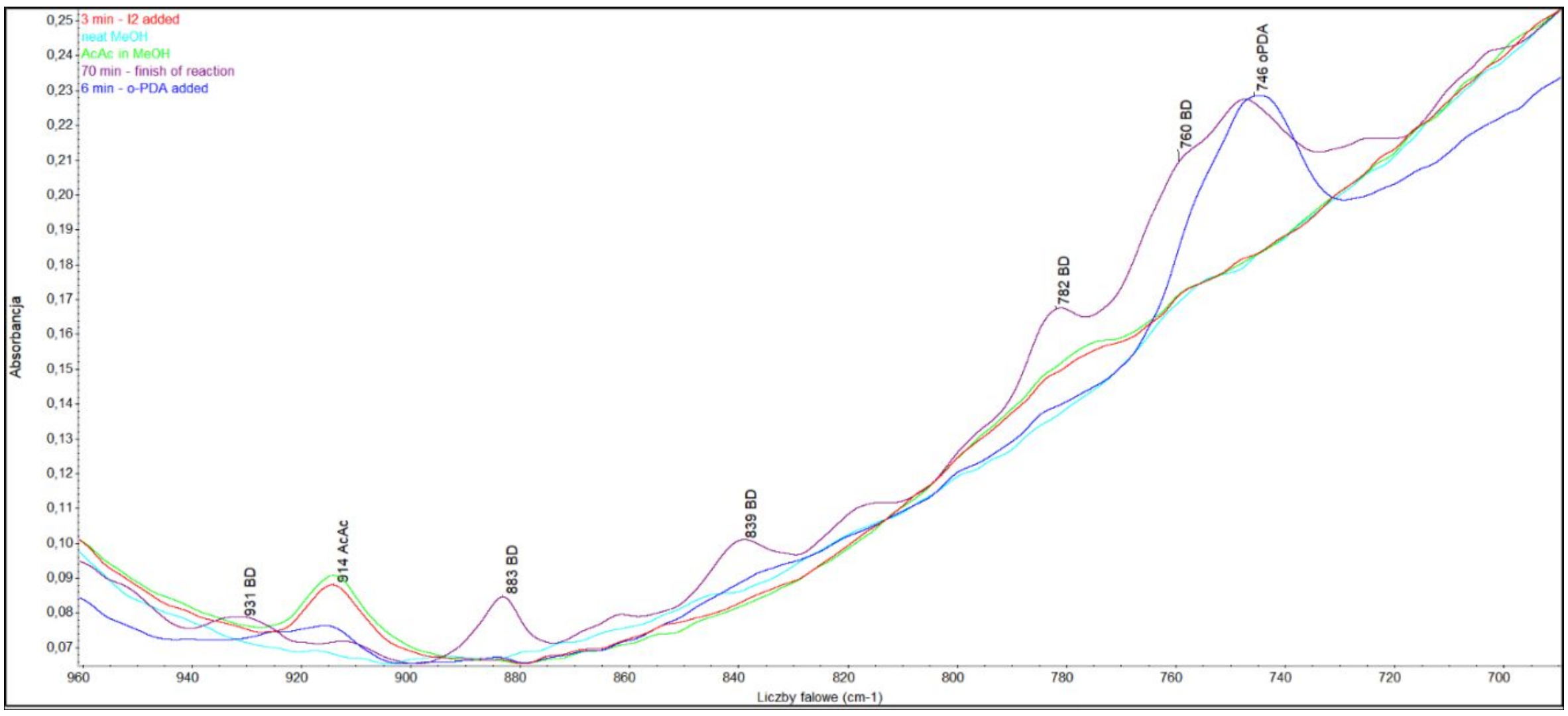


b) MeCN:

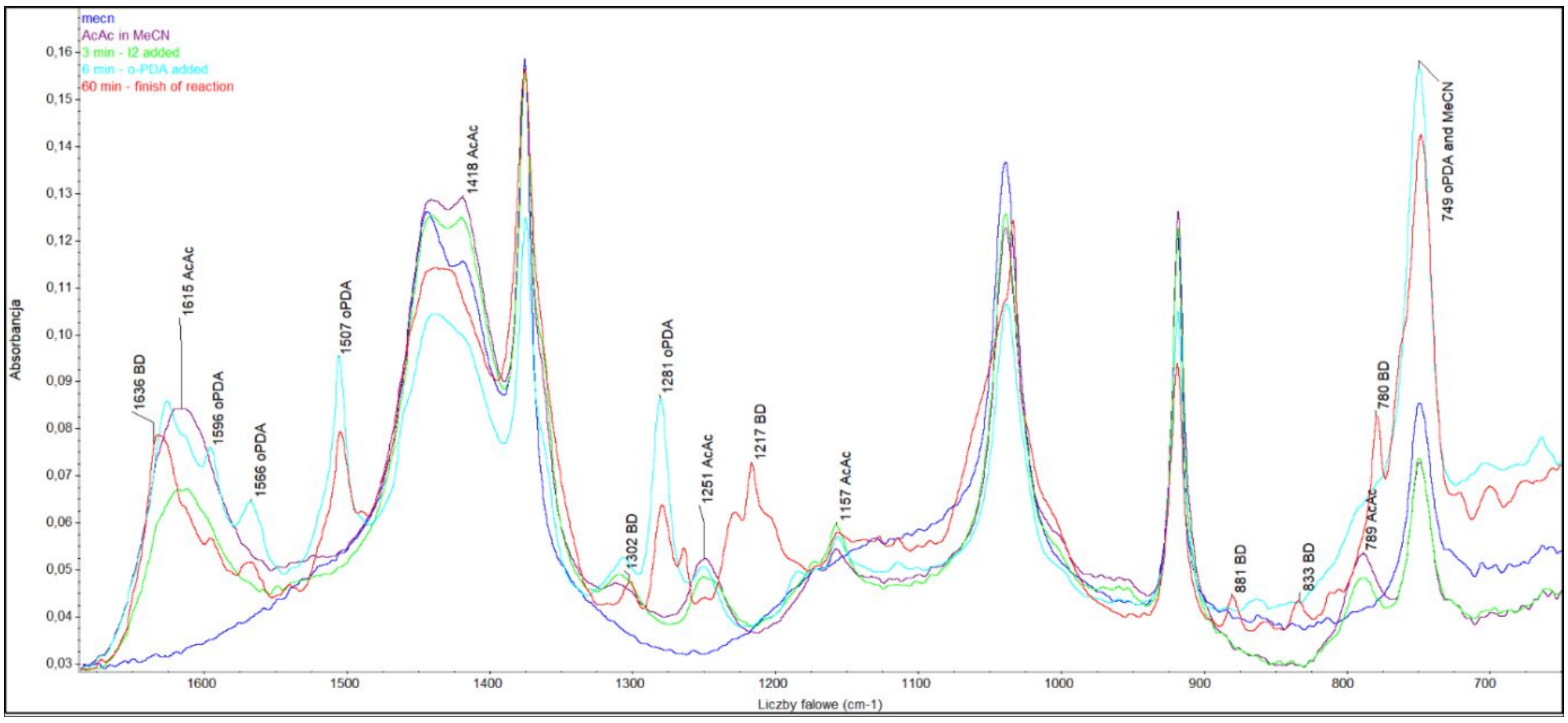


2.2. **2,4-Diethyl-2-methyl-2,3-dihydro-1H-1,5-benzodiazepine (2,4-Et-2-Me-1,5-BD**)

**Figure S25**. Synthesis of 2,4-Diethyl-2-methyl-2,3-dihydro-1H-1,5-benzodiazepine: *in situ* FTIR spectra of start and finish of the reaction.

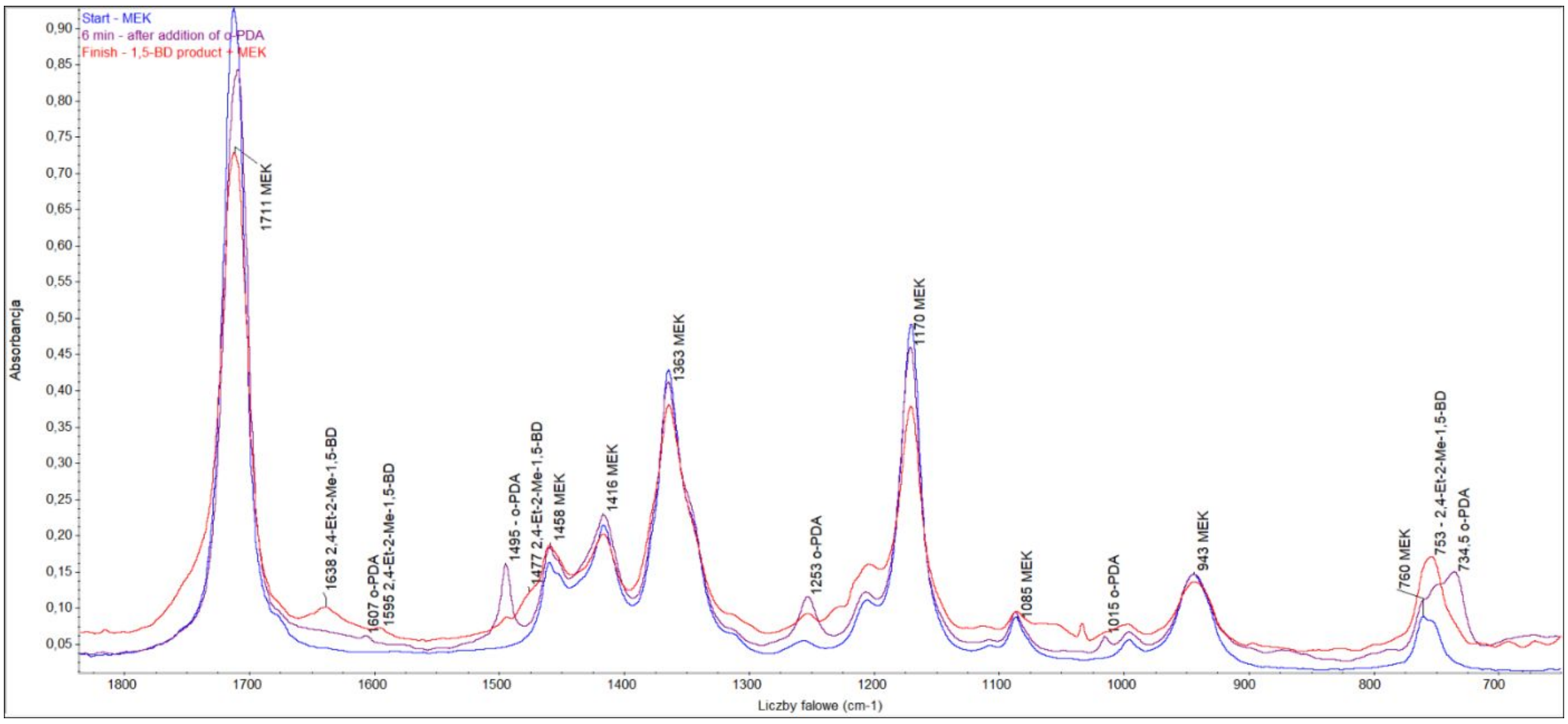


**Figure S26.** Synthesis of 2,2,4,8-tetramethyl-2,3-dihydro-1H-1,5-benzodiazepine: *in situ* FTIR spectra of start and finish of the reaction.

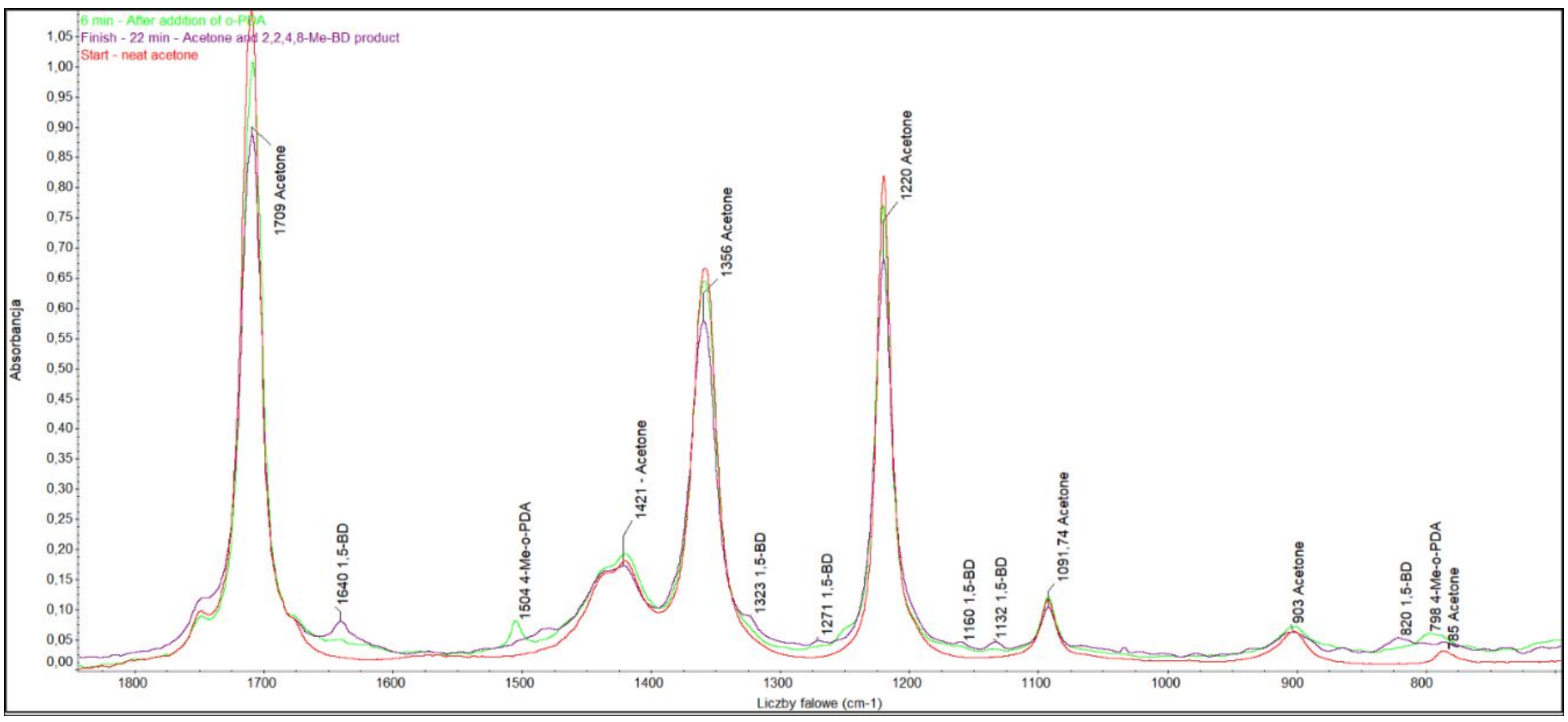


**Figure S27-29.** Synthesis of 2-Methyl-2,4-diphenyl-2,3-dihydro-1H-1,5-benzodiazepine in a) MeCN with $Sc(OTf)_3$ catalyst; b) in MeOH with $I_2$ as catalyst:

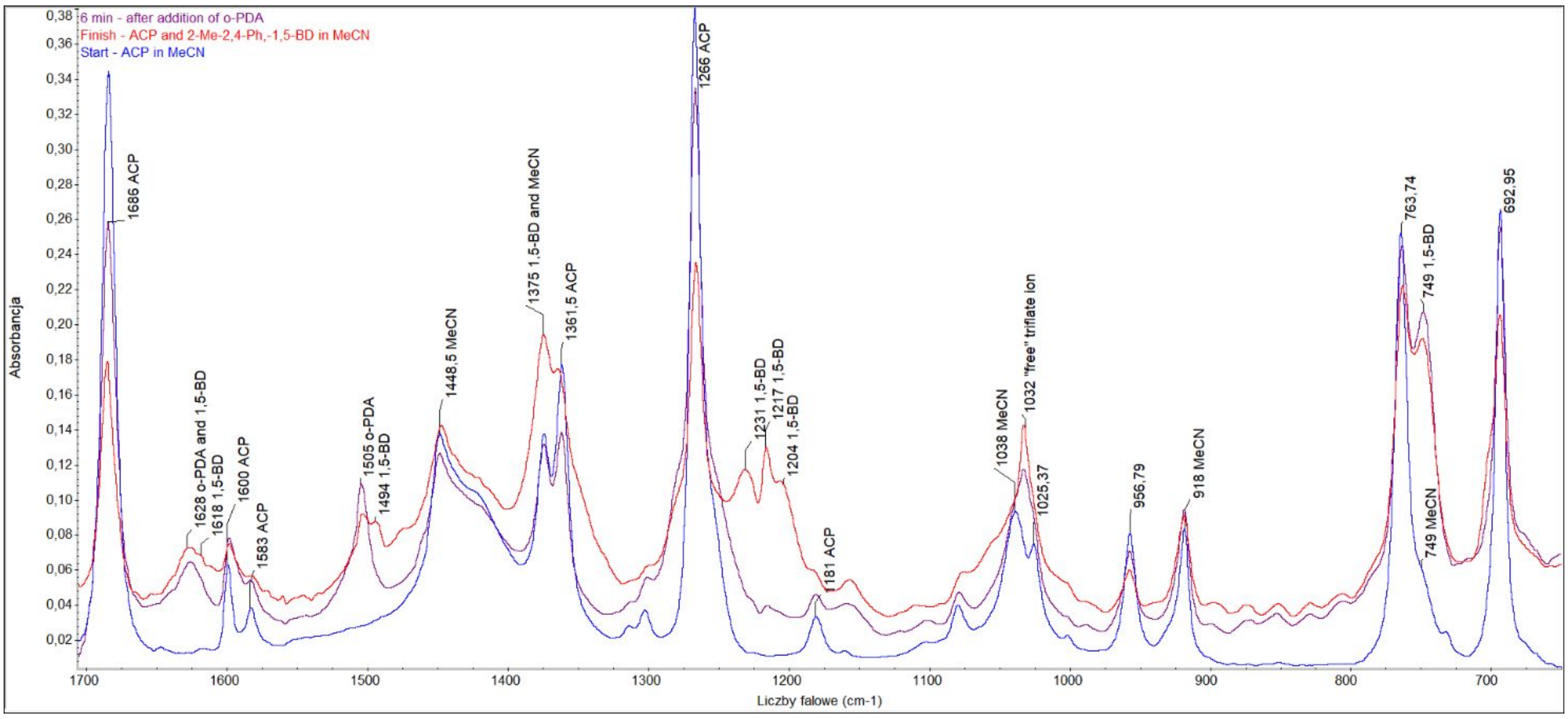

a)

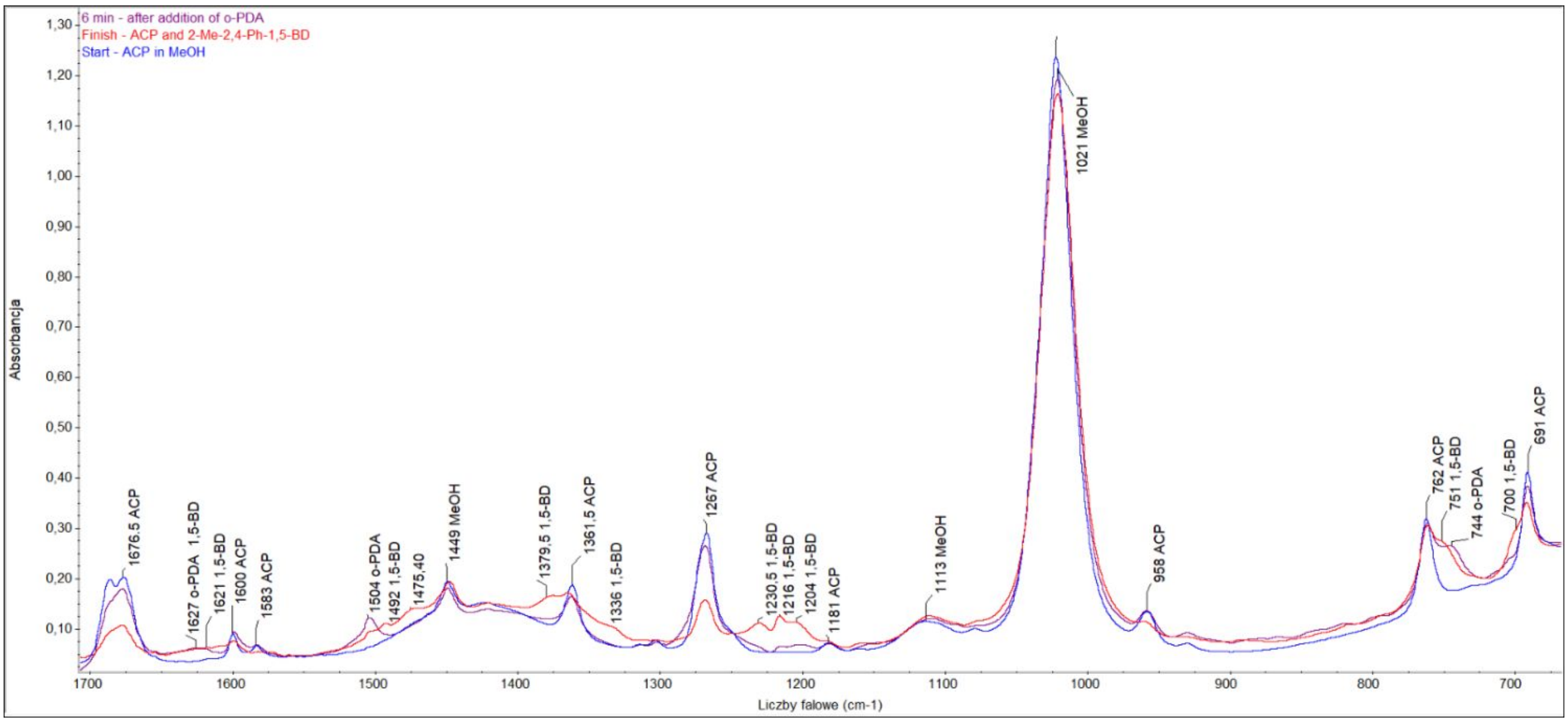

b)

## 3. Kinetic profiles

**Figure S30.** Synthesis of 2,4-diethyl-2-methyl-2,3-dihydro-1H-1,5-benzodiazepine: reaction profiles. The synthesis was conducted in methyl ethyl ketone as solvent and substrate, using o-phenylenediamine and the most effective catalysts—molecular iodine (5 mol%) and scandium triflate (3 mol%)—under microwave irradiation (200 W) and under reflux conditions.

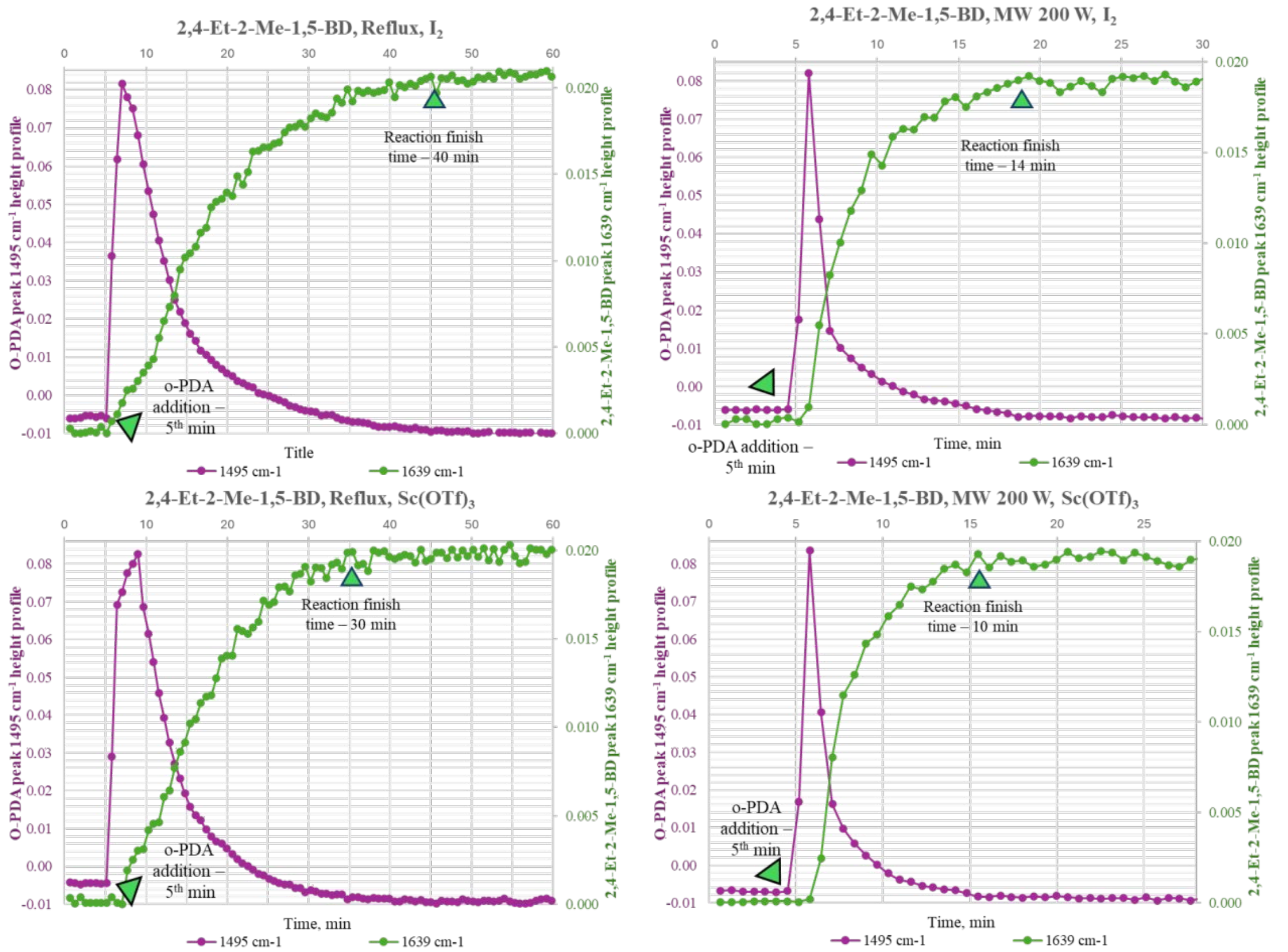


**Figure S31.** Synthesis of 2,2,4,8-tetramethyl-2,3-dihydro-1H-1,5-benzodiazepine: reaction profiles.

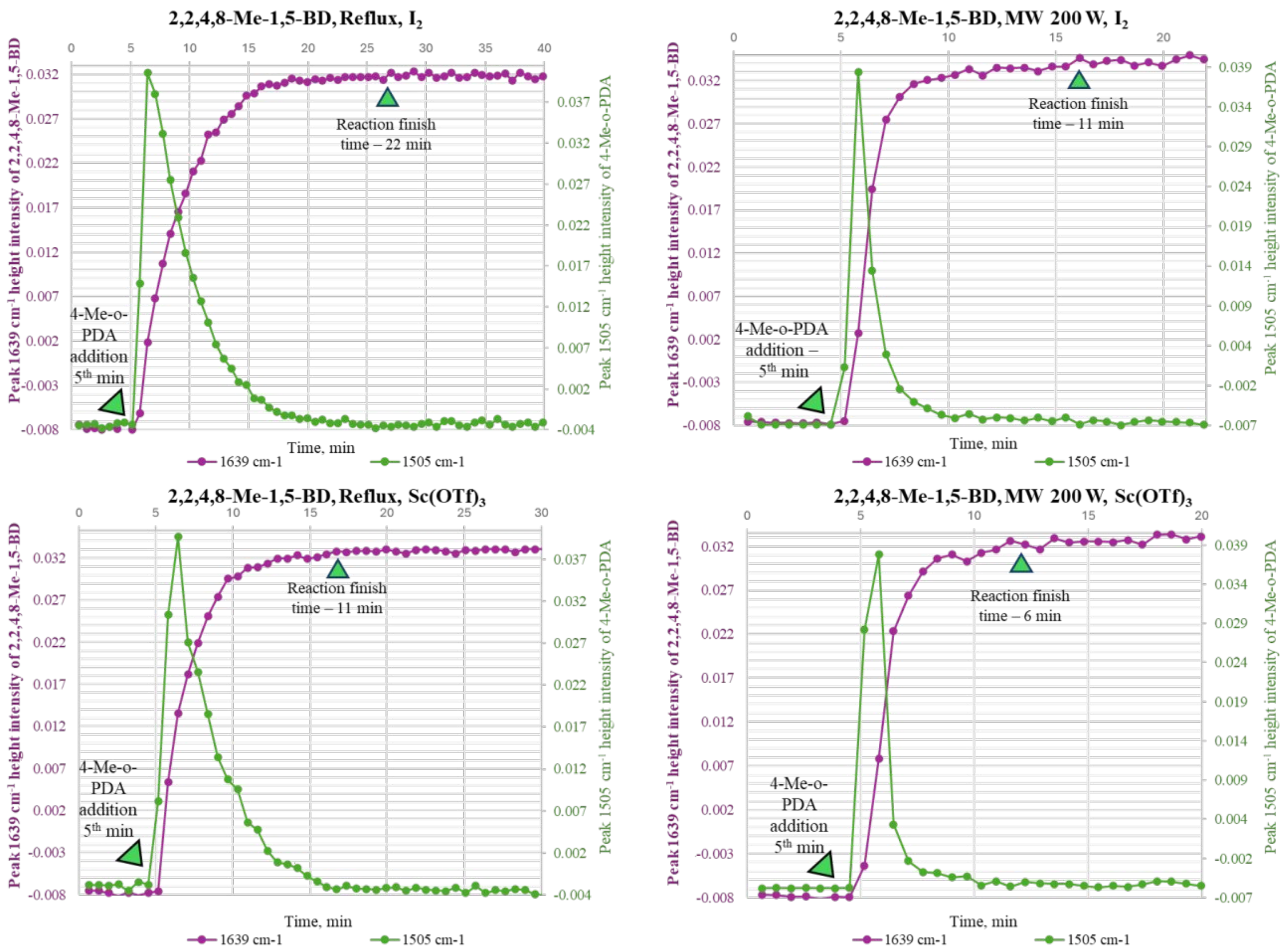

**Figure S32.** Synthesis of 2-methyl-2,4-diphenyl-2,3-dihydro-1H-1,5-benzodiazepine: reaction profiles.

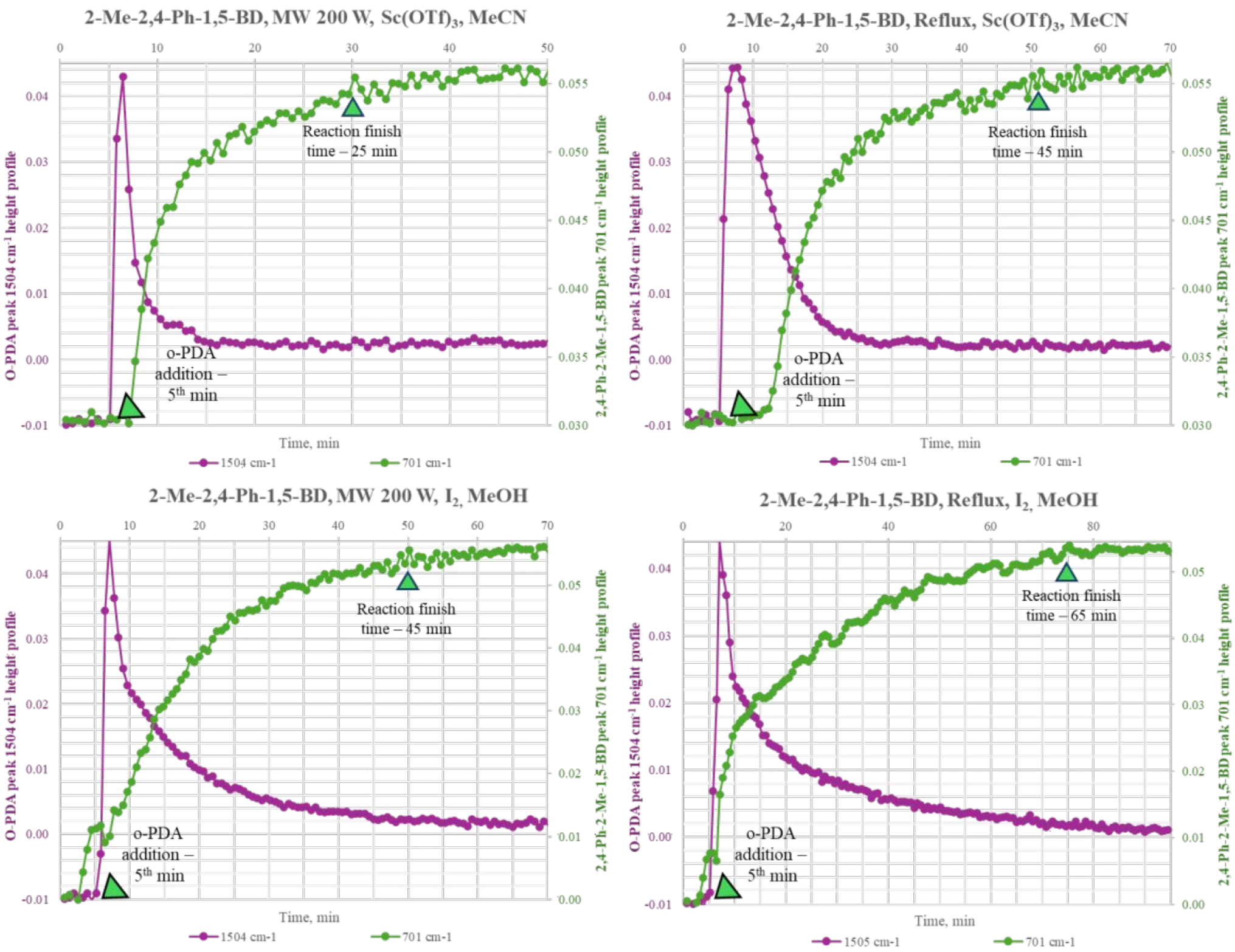


## 4. Experimental setup: details, Montmorillonite K10 fouling FTIR imaging.

**Figure S33.** Photo of experimental setup: 1- ATR-FTIR spectrometer connected by 2 - fibre optic probe with 3 - multifunctional chemical reactor, equipped with 4 -condenser and 5 temperature probe.

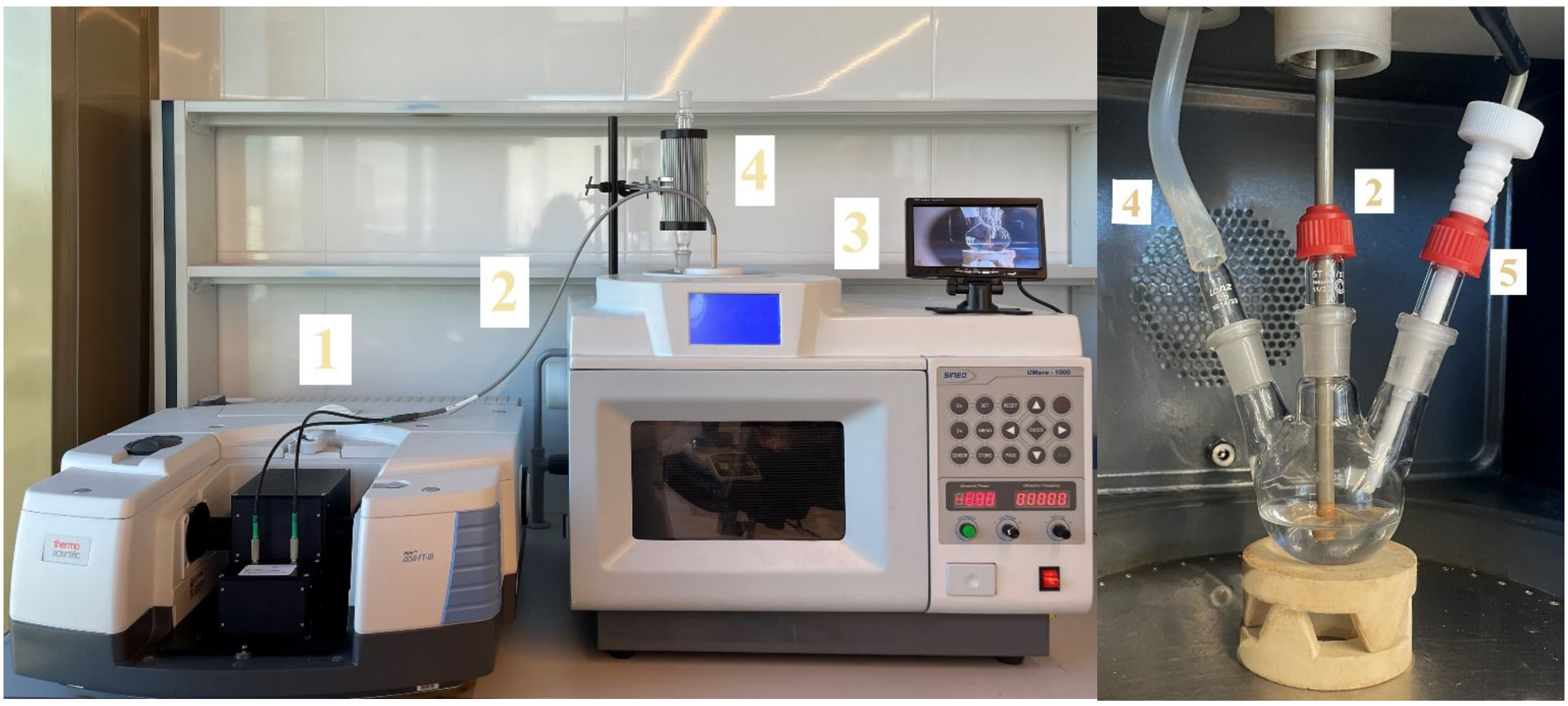

**Figure S34.** Time-resolved *in situ* ATR-FTIR spectral overlay demonstrating the progressive accumulation of Montmorillonite K10 clay on the diamond sensor in MeCN at 50 °C.

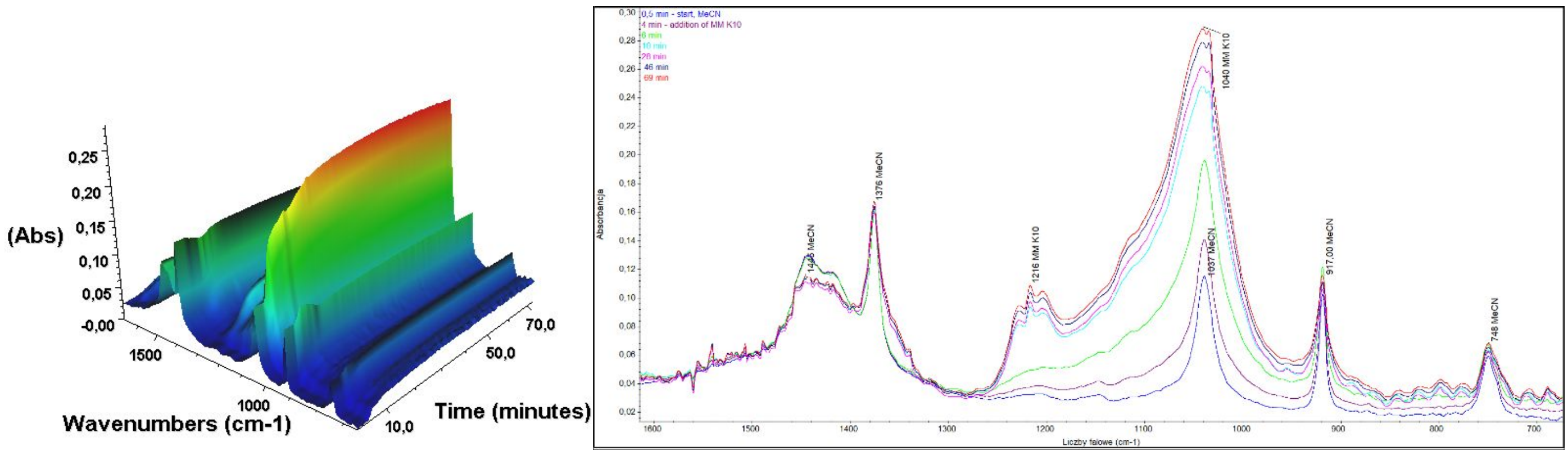


**Figure S35.** Kinetic height profile of the 1041 $cm^{-1}$ Si–O stretching band, illustrating the rapid initial adhesion and continuous accumulation of the solid clay layer within the evanescent sensing field.

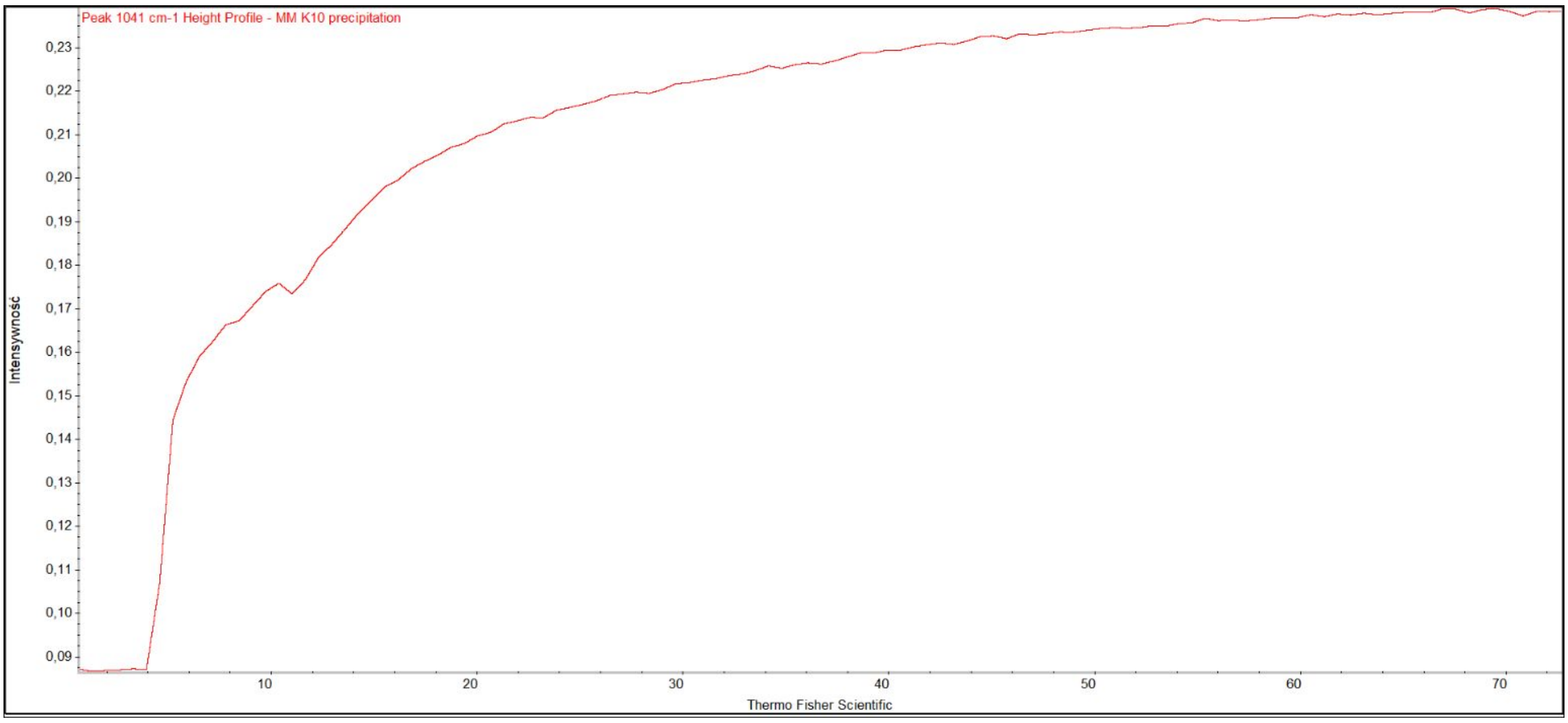

Supplement: Supplementary file 1 [file ao6c07314_si_001.pdf]
